# Bioinformatics and system biology approach to identify potential common pathogenesis for COVID-19 infection and sarcopenia

**DOI:** 10.3389/fmed.2024.1378846

**Published:** 2024-06-24

**Authors:** Jun Zhong, Hui Yuan, Jinghong Yang, Yimin Du, Zheng Li, Xu Liu, Haibo Yang, Zhaojun Wang, Zi Wang, Lujun Jiang, Zhiqiang Ren, Hongliang Li, Zhong Li, Yanshi Liu

**Affiliations:** ^1^School of Clinical Medicine, Southwest Medical University, Luzhou, Sichuan, China; ^2^Department of Orthopedics, Affiliated Hospital of Southwest Medical University, Luzhou, Sichuan, China

**Keywords:** sarcopenia, COVID-19, bioinformatics, pathogenesis, biomarkers

## Abstract

Sarcopenia is a condition characterized by age-related loss of muscle mass and strength. Increasing evidence suggests that patients with sarcopenia have higher rates of coronavirus 2019 (COVID-19) infection and poorer post-infection outcomes. However, the exact mechanism and connections between the two is unknown. In this study, we used high-throughput data from the GEO database for sarcopenia (GSE111016) and COVID-19 (GSE171110) to identify common differentially expressed genes (DEGs). We conducted GO and KEGG pathway analyses, as well as PPI network analysis on these DEGs. Using seven algorithms from the Cytoscape plug-in cytoHubba, we identified 15 common hub genes. Further analyses included enrichment, PPI interaction, TF-gene and miRNA-gene regulatory networks, gene-disease associations, and drug prediction. Additionally, we evaluated immune cell infiltration with CIBERSORT and assessed the diagnostic accuracy of hub genes for sarcopenia and COVID-19 using ROC curves. In total, we identified 66 DEGs (34 up-regulated and 32 down-regulated) and 15 hub genes associated with sarcopenia and COVID-19. GO and KEGG analyses revealed functions and pathways between the two diseases. TF-genes and TF-miRNA regulatory network suggest that FOXOC1 and hsa-mir-155-5p may be identified as key regulators, while gene-disease analysis showed strong correlations with hub genes in schizophrenia and bipolar disorder. Immune infiltration showed a correlation between the degree of immune infiltration and the level of infiltration of different immune cell subpopulations of hub genes in different datasets. The ROC curves for ALDH1L2 and KLF5 genes demonstrated their potential as diagnostic markers for both sarcopenia and COVID-19. This study suggests that sarcopenia and COVID-19 may share pathogenic pathways, and these pathways and hub genes offer new targets and strategies for early diagnosis, effective treatment, and tailored therapies for sarcopenia patients with COVID-19.

## Introduction

Sarcopenia is a progressive and systemic disease of extreme skeletal muscle dysfunction (1) characterized by reduced muscle mass/quantity and muscle strength that is observed in both physiological and pathological processes (2, 3). According to the most recent guidelines from the European Working Group on Sarcopenia in the Elderly (EWGSOP), the primary diagnostic criteria for sarcopenia have shifted to include diminished muscle strength and function, rather than solely the loss of muscle mass. Additionally, impaired physical performance is now recognized as a key marker of advanced sarcopenia (4, 5). Studies have shown that sarcopenia occurs with age and the effects of many long-term conditions (2, 5), and is associated with decreased mobility, increased morbidity and increased mortality (6). Currently, sarcopenia affects over 50 million people and this number is expected to reach 500 million by 2050 (7, 8). Sarcopenia is estimated to affect 10–16% of older adults worldwide (9). One study reported that the prevalence of sarcopenia patients (EWGSOP definition) ranged from 8 to 36% in those aged <60 years and from 10 to 27% in those aged ≥60 years (10). The pathogenesis of sarcopenia is complex, with high morbidity and mortality, and it is currently believed that the pathogenesis of sarcopenia may be related to factors such as reduced satellite cell numbers and aging (11, 12), mitochondrial dysfunction (13), loss of motor neurons, decreased activity of neuromuscular junctions (14), endocrine alterations (15), and weight loss with decreased appetite (16), or a combination of these factors (17).

COVID-19 is a multi-organ infectious disease caused by Severe Acute Respiratory Syndrome Coronavirus 2 (SARSCoV-2), particularly severe pneumonia and acute respiratory distress syndrome (18). As of September 7, 2023, the World Health Organization reported 770,437,327 confirmed cases, including 6,956,900 deaths.^1^ COVID-19 can pose a serious health burden individuals, especially the elderly and those with underlying medical conditions such as advanced age (19), chronic kidney disease, diabetes mellitus, hypertension (20), and cancer (21, 22) are risk factors that have been widely documented to be responsible for COVID-19 infections and deaths. COVID-19 is characterized by severe inflammation and a highly muscle catabolic state, which affects the body’s metabolic stress and profound changes in body composition. Researchers have attempted to prevent and treat COVID-19 by investigating drugs and developing vaccines, but its intervention in adverse body states (e.g., sarcopenia) may facilitate the treatment of COVID-19 (23, 24).

A study indicated that patients with sarcopenia experienced a higher prevalence of infection and poorer prognosis during the COVID-19 pandemic (23). Patients with sarcopenia have impaired immune cells (e.g., peripheral monocytes, neutrophils, and natural killer lymphocytes) (25), which result in the production of aberrant myofactors such as IL-6, IL-7, IL-15, or LIF (26), which ultimately lead to muscle catabolism and immune senescence (24, 25). However, the onset and progression of sarcopenia is accelerated during COVID-19 infection due to increased muscle atrophy and inhibition of muscle synthesis caused by severe inflammatory response and metabolic stress (27), decreased physical activity and inadequate nutrient intake (28). Hospitalization, protein deficiency, and corticosteroid therapy during COVID-19 infection have been reported in several studies that often lead to the rapid progression of sarcopenia in patients with severe COVID-19 infection (29, 30). Skeletal muscle regulates immune system function through myokine signaling and expression of immunoregulatory surface molecules. Immune cells in turn severely affect muscle mass and function (25). This indicates that the interaction between sarcopenia and COVID-19 may be bidirectional, potentially creating a vicious cycle.

An increasing number of studies indicate a strong relationship between sarcopenia and COVID-19 infection; however, the mechanisms have not been fully elucidated. This study utilizes bioinformatics, R software, and several large databases to analyze the common DEGs and hub genes of sarcopenia and COVID-19 in terms of expression differences, functional enrichment, regulatory networks, disease drug prediction, and immune infiltration. This analysis will help further understand the potential co-pathogenesis of sarcopenia and COVID-19 and to screen for biomarkers and drug candidates.

## Materials and methods

### Data collection

RNA-seq data for patients with sarcopenia (GSE111016) and COVID-19 infections (GSE171110) were obtained from the GEO database.^2^ GEO is one of the largest public database that includes microarray data and high-throughput gene expression data submitted by research institutions around the world (31). Both datasets used the GPL 16791 (Illumina NextSeq 500) high-throughput sequencing platform to extract RNA sequences. The GSE111016 dataset includes 20 muscle biopsies from healthy testers and 20 muscle biopsies from patients with sarcopenia (32). The GSE171110 dataset includes 44 COVID- 19 patients and 10 healthy donors with whole blood gene expression profiling data (33).

### Identification of common DEGs between sarcopenia and COVID-19

The “limma” package (version 4.3.1) of the R software (version 4.3.1) was used to select DEGs between COVID-19 and non-COVID-19 and between sarcopenia and non-sarcopenia. Because of the differences in sample size and data quality, different difference multiples criteria were selected to ensure statistical significance and biological relevance of the results. In the sarcopenia dataset, genes with *p* < 0.05 and a fold change >1.2 were identified as DEGs; for COVID-19, genes with *p* < 0.05 and a fold change >2 were identified as DEGs. In the sarcopenia dataset, the DEG of log_2_FC<−0.263 was considered down-regulated, whereas log_2_FC>0.263 was considered up-regulated. For COVID-19, the DEG of log_2_FC<−1 was considered down-regulated, whereas log_2_FC>1 was considered up-regulated. The “Pheatmap” (version 1.0.12), “EnhancedVolcano” and “ggplot2” packages of the R software were applied to generate the heatmaps and volcano maps. Common DEGs for GSE111016 and GSE171110 were then obtained using the online VENN analysis tool.^3^

### Enrichment analysis of gene ontology and pathways

EnrichrR^4^ is a comprehensive resource for analyzing gene sets generated from genome-wide experiments, containing a total of 180,184 annotated gene sets from 102 gene set libraries (34). GO and pathway enrichment analyses were performed using EnrichR online tools [the Kyoto Encyclopedia of Genes and Genomes (KEGG)] to specify shared functions and pathways between sarcopenia and COVID-19. The GO terminology consists of three categories: biological process (BP), cellular component (CC), and molecular function (MF). A *p*-values <0.05 was considered significantly enriched.

### PPI network construction

STRING^5^ (version 12.0) is a database for studying protein–protein association networks, with an expanded information coverage of more than 12,535 species, 59.3 million proteins, and 20 billion interactions, integrating experimental interaction evidence and computational interaction prediction information, with the goal of realizing a comprehensive and objective global network (35). We performed PPI network analysis of the common DEG using the STRING database to construct differentially expressed and potential interactions of genes with interaction scores >0.15. The protein–protein interaction networks constructed in the String database were then imported into Cytoscape (version: 3.9.1) software for visualization (36, 37).

### Identification and analysis of hub genes

In a PPI consisting of nodes, edges and their connections, the most entangled nodes were considered hub gene. Cytohubba^6^ is a novel plugin for Cytoscape that provides 11 topological analysis methods to rank nodes in a network (38). We applied seven algorithms (Closeness, MCC, Degree, MNC, Radality, Stress and EPC) to finally intersect them to select hub gene.

GeneMANIA^7^ is a flexible and user-friendly website that uses large amounts of genomics and proteomics data to generate hypotheses about gene function, analyze gene lists and prioritize genes for functional analysis (39). We used it to construct co-expression networks of identified central genes.

### Construction of TF-gene and miRNA-gene regulatory network

TFs are proteins that control the transcription of DNA into RNA by attaching to specific DNA sequences. miRNAs are mainly involved in the regulation of protein expression by binding to target sites on mRNA transcripts and inhibiting their translation, making them essential for regulating biomolecules (40, 41). We visualized the co-regulatory network of hub genes through the NetworkAnalyst platform, which has been widely used as a bioinformatics tool (42). We used NetworkAnalyst to extract microRNAs interacting with hub genes from the miRTarBase database (43) and construct a DEG-microRNA (miRNA) interaction network, and then localized TFs binding to hub genes through the JASPAR database (44) and constructed a DEG-transcription factor interaction network. We performed GRN analysis using hub-DEG to reveal transcription elements and miRNAs that regulate DEG at the post-transcriptional level.

### Gene-disease association analysis

DisGeNET is a comprehensive knowledge management platform that integrates and normalizes data on disease-associated genes and variants from multiple sources, including the scientific literature, and can be used to study the molecular basis of specific human diseases and their complications, to analyze the characterization of disease genes and to validate the performance of computationally predicted disease genes (45). It currently covers more than 24,000 diseases and traits, 17,000 genes and 117,000 genomic variants (46). We examined gene-disease relationships using the DisGeNET database through NetworkAnalyst to reveal diseases and their complications associated with central genes.

### Correlation analysis of hub gene expression with immune infiltration

To explore the immune infiltration of multiple immune cells including T cells, B cells, NK cells, monocytes, macrophages, neutrophils, and dendritic cells in GSE111016 and GSE171110 peripheral blood (47), single-sample gene set enrichment analysis (ssGSEA) (48) of 28 immune gene sets was performed using the “GSVA” R package, assessing the immunological characteristics of the samples. The Vioplot and pheatmap R packages were used for visualization. Finally, the Pearson correlation coefficient determined the correlation between hub genes and different immune infiltrating cells, visualized through the ggplot2 package.

### Target drugs analysis

The DSigDB database is a new gene set resource that links drugs/compounds to their target genes. It currently has 22,527 gene sets consisting of 17,389 unique compounds, covering 19,531 genes (49). We detected 15 drug molecules identified based on hub genes from the DSigDB database on the Enrichr^8^ platform. These drugs represent possible common drugs used for sarcopenia and COVID-19.

### ROC curves of hub genes

The receiver operating characteristic (ROC) curve is a useful tool for evaluating classifiers in biomedical and bioinformatics applications. In this study, R was established by “pROC” based on the expression profile data of hub genes (50). The area under the ROC curve (AUC) was used to evaluate the diagnostic value of candidate hub genes separately.

## Results

### Identification of DEGs and shared genes between sarcopenia and diabetes

**Figure 1:**
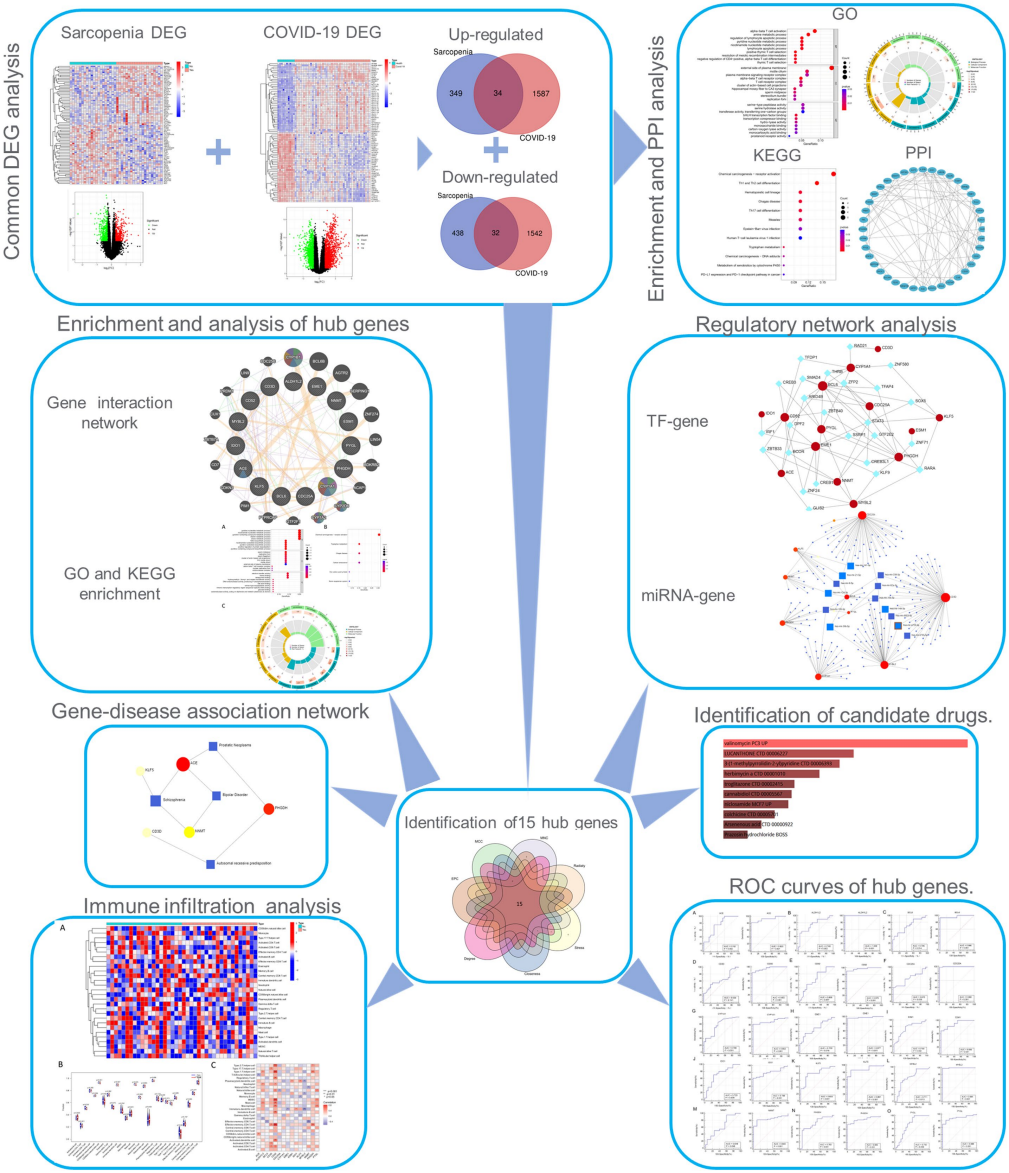
Workflow of the whole study. DEGs, differentially expressed genes; GO, Gene Ontology; PPI, protein–protein interaction; TF, transcription factor.

**Figure 2:**
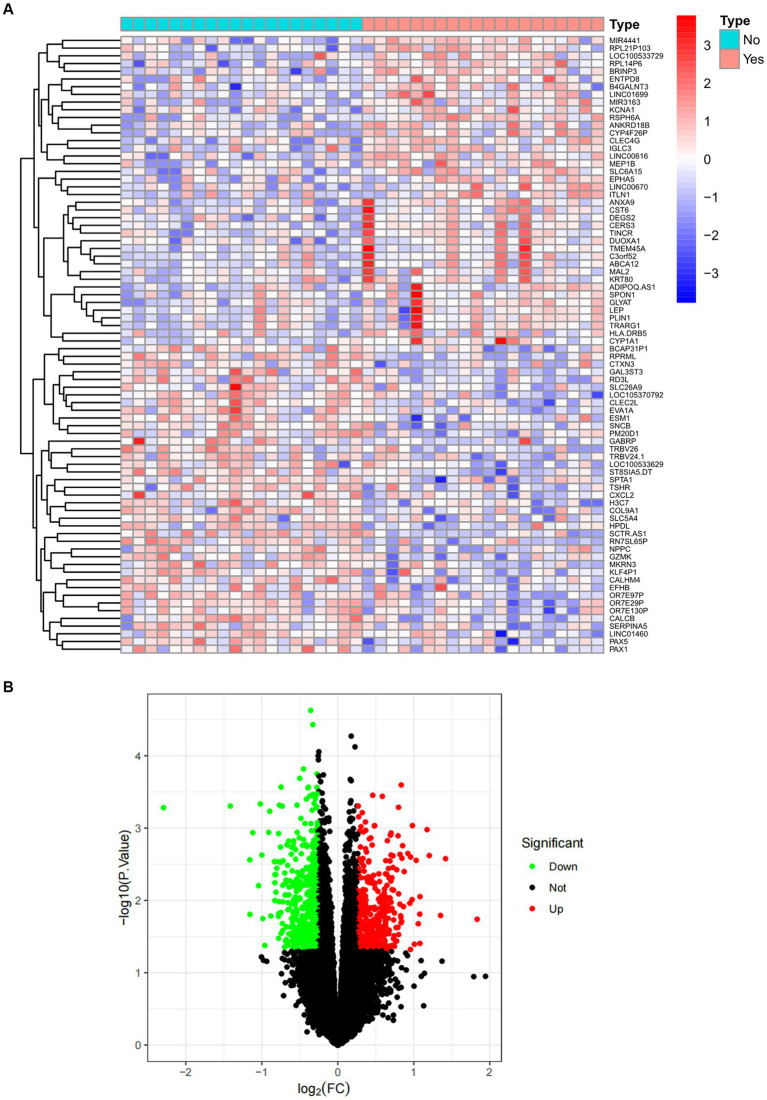
Expression characteristics of DEG in sarcopenia. **(A)** Heat map and **(B)** volcano plot present the DEGs identified between sarcopenia patients and normal controls (|log_2_FC|>0.263 defined as the screening criterion to obtain DEGs in sarcopenia). Blue color indicates low expression values and red color indicates high expression values.

**Figure 3:**
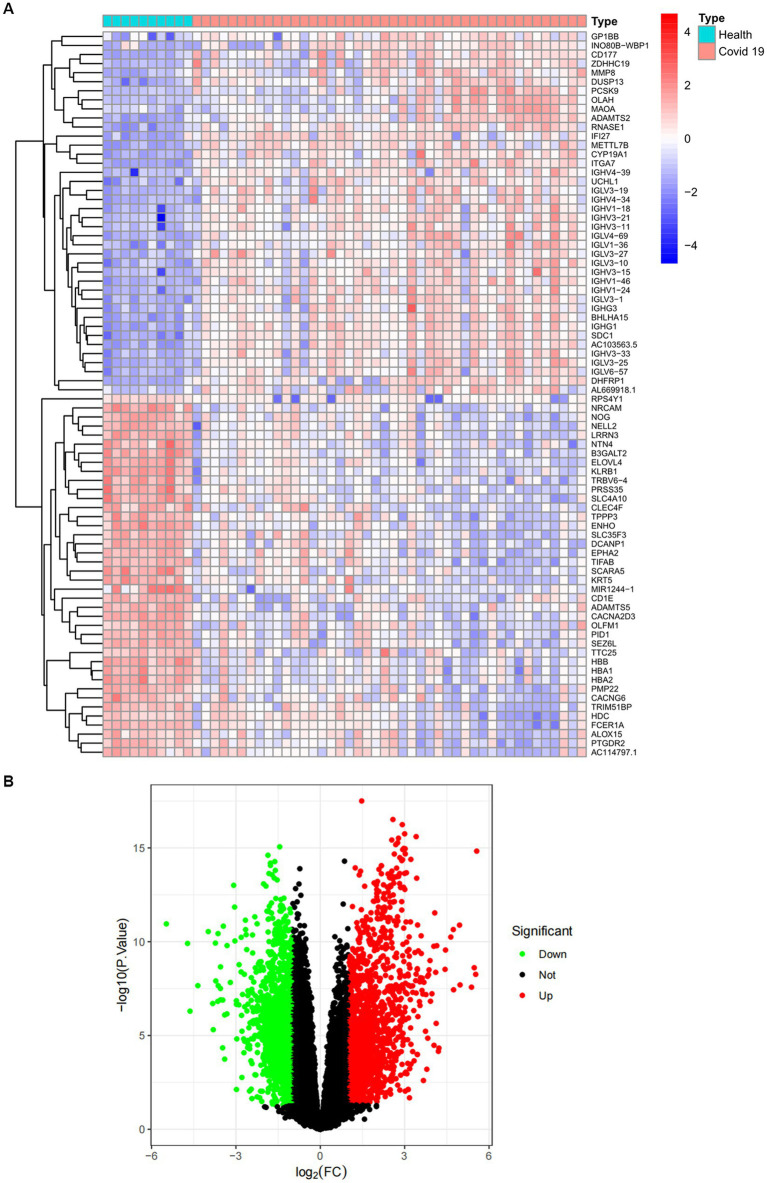
Expression characteristics of DEGs in COVID-19 patients. **(A)** Heat map and **(B)** volcano plot present the DEGs identified between COVID-19 patients and normal controls (|log_2_FC|>1.0 is defined as a screening criterion to obtain a DEG for COVID-19). Blue indicates a low expression value and red indicates high expression value.

**Figure 4:**
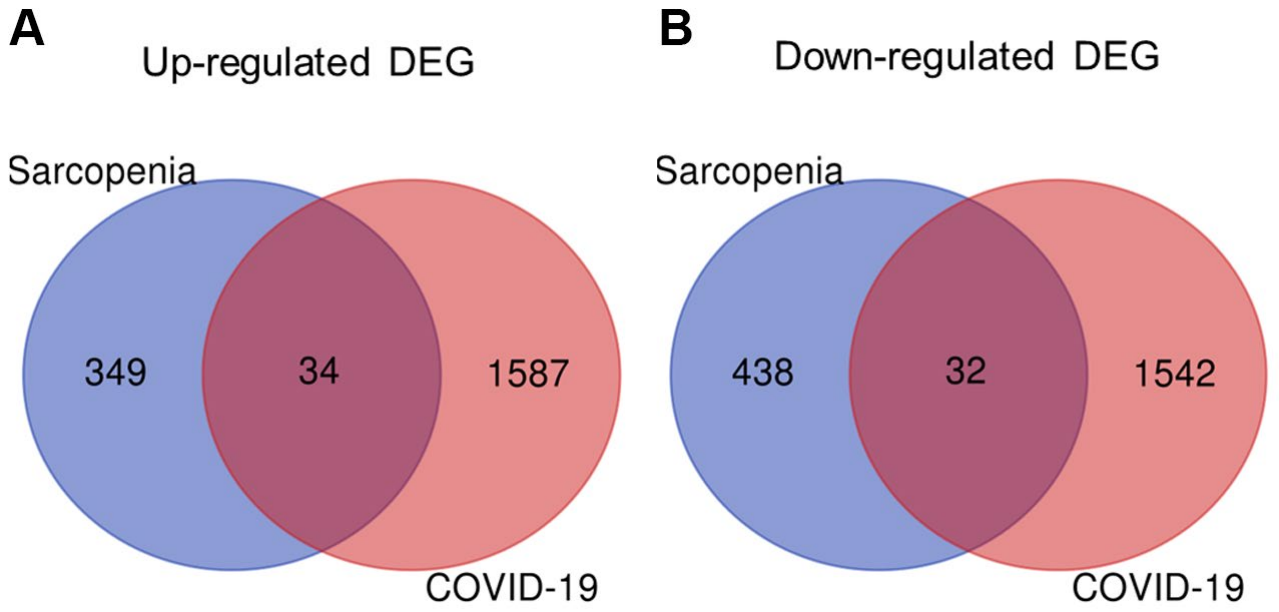
Identification of shared DEGs between sarcopenia and COVID-19. Venn diagram showing two datasets owning **(A)** 34 common up-regulated DEGs and **(B)** 32 common down-regulated DEGs.

The overall flowchart of this study is shown in Figure 1A. A total of 853 differential genes (DEGs) were identified based on the sarcopenia dataset GSE111016 using the limma R software package, of which 383 were upregulated and 470 were downregulated. The heatmap plot shows the identified DEGs (Figure 2A), and the heat map shows the distribution of the top 25 DEGs for up- and down-regulation in sarcopenia patients and non-sarcopenia patients, respectively (Figure 2B). In addition, a total of 3,195 DEGs were obtained from the COVID-19 dataset GSE171110, of which 1,621 genes were up-regulated and 1,574 genes were down-regulated. The top 25 DEG heat maps of up- and down-regulation and the volcano map of DEG are shown in Figures 3A,B. The intersection of DEGs from the GSE111016 and GSE171110 datasets was visualized by a Wayne’s diagram. In total, 34 common up-regulated DEGs and 32 common down-regulated DEGs are shown (Figures 4A,B).

### Gene ontology and pathway enrichment analysis

**Figure 5:**
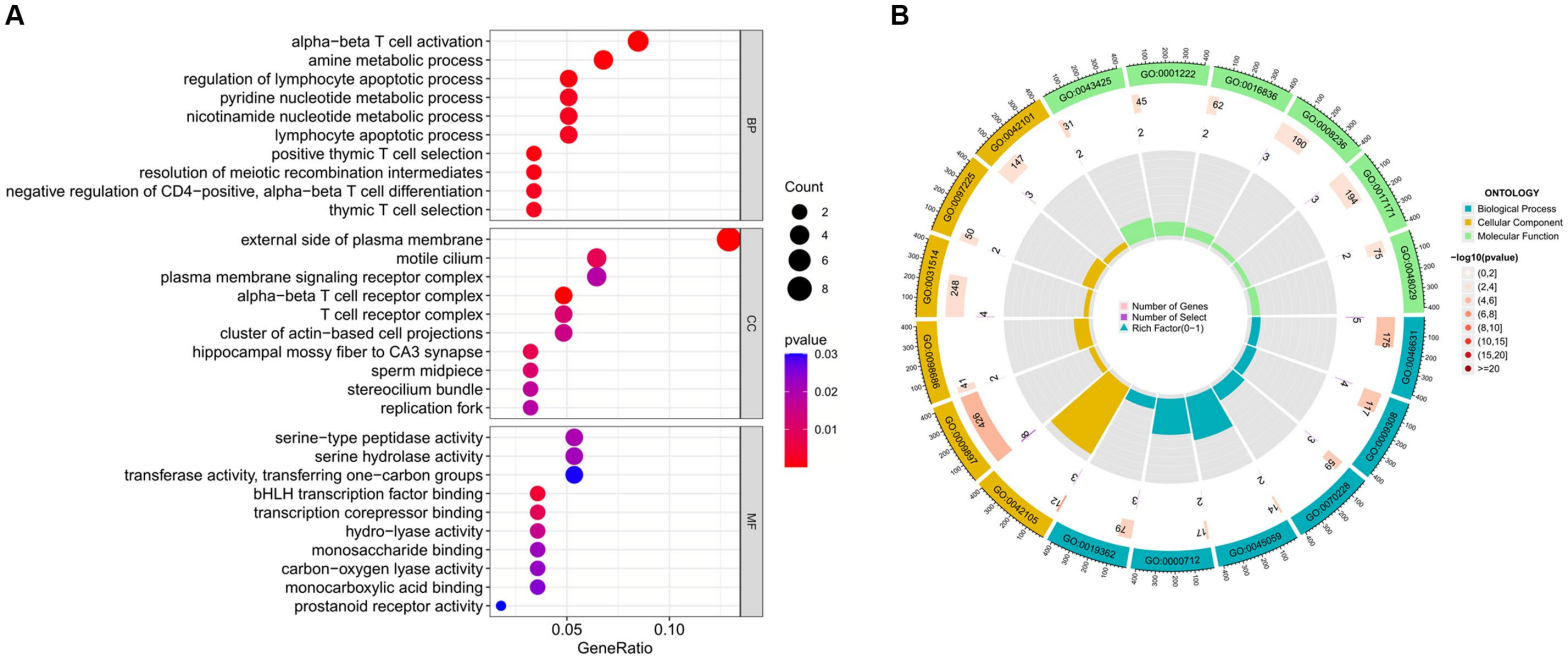
GO functional enrichment analysis of common genes between sarcopenia and COVID-19. **(A)** Bubble plot showing enriched GO terms. **(B)** Circle plots showing enriched GO terms. Results are shown by −log 10 (*p*-value).

Enrichment analysis aids in further understanding the biological functions of genes shared between COVID-19 and sarcopenia patients. By analyzing Gene Ontology (GO) and the Kyoto Encyclopedia of Genes and Genomes (KEGG), we predicted the functions of DEGs and their common differential genes in COVID-19 and sarcopenia. GO analysis predicted gene functions in three categories: biological processes, cellular components, and molecular functions. Results indicated that key biological processes include alpha-beta T cell activation, amine metabolic processes, and the regulation of lymphocyte apoptotic processes. Primary cellular components involved are the alpha-beta T cell receptor complex, the external side of the plasma membrane, and the hippocampal mossy fiber to CA3 synapse. Predominant molecular functions include binding of bHLH transcription factors, transcription corepressor binding, and hydro-lyase activity (Figure 5; Supplementary Figure S2).

**Figure 6:**
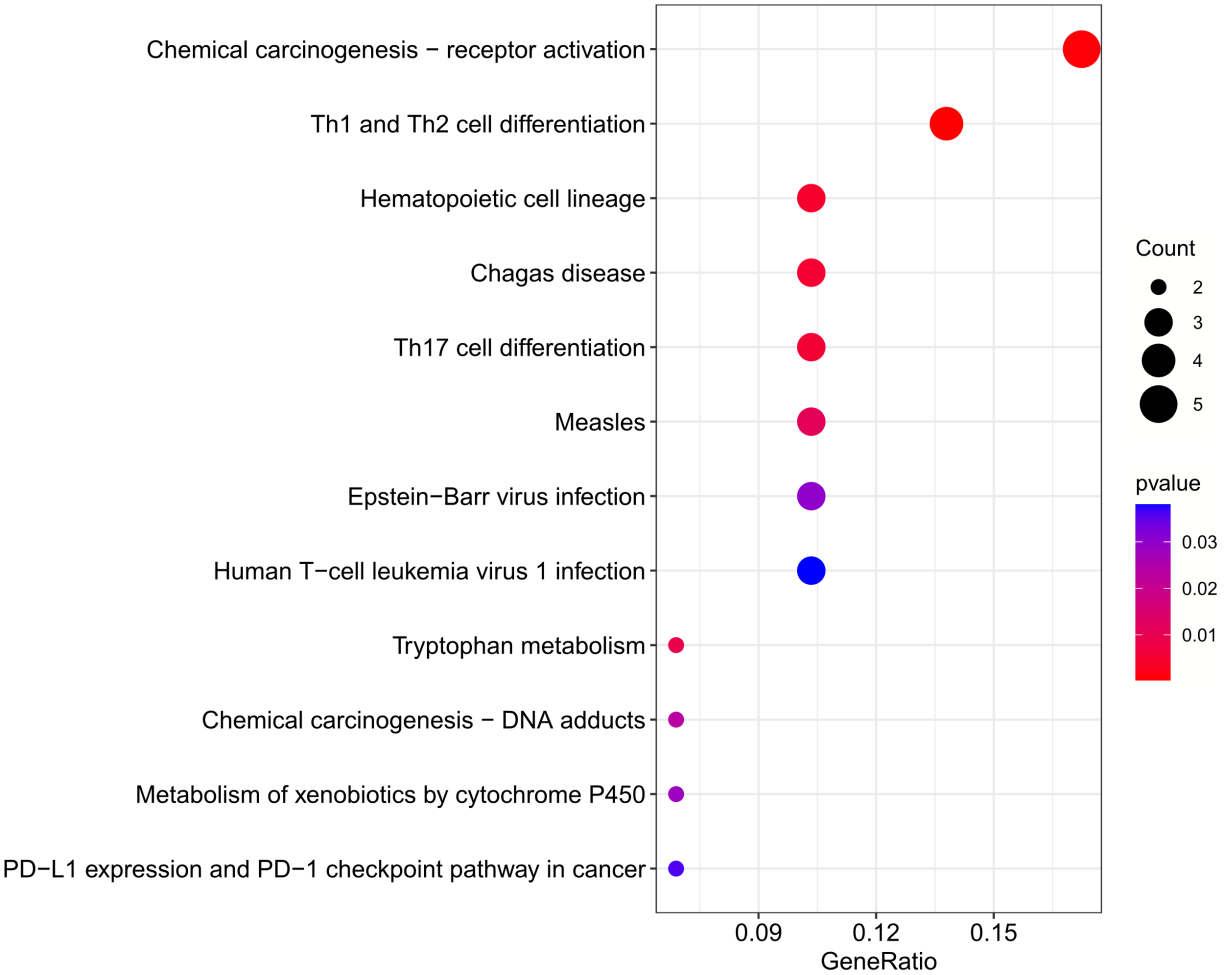
Functional enrichment analysis of the common gene KEGG between sarcopenia and COVID-19. Results are shown as −log 10 (*p* value).

The enrichment pathways of common DEGs between COVID-19 and sarcopenia were collected from the KEGG database and visualized in Figure 6. KEGG enrichment analysis showed that common genes were mostly enriched in the Th1 and Th2 cell differentiation, chemical carcinogenesis – receptor activation, and hematopoietic cell lineage pathways (Figure 6).

### PPI network analysis

We utilized the STRING database to construct a Protein–Protein Interaction (PPI) network analysis of the shared genes, aiming to predict interactions and adhesion pathways among common DEGs. The network was then imported into Cytoscape for visualization to explore their potential interactions. As expected (Supplementary Figure S3), the PPI network of shared DEGs comprises 40 nodes and 82 edges, and it was subsequently used in subsequent steps for identifying hub genes and detecting drug molecules for both COVID-19 and sarcopenia.

### Identification of hub genes

**Figure 7:**
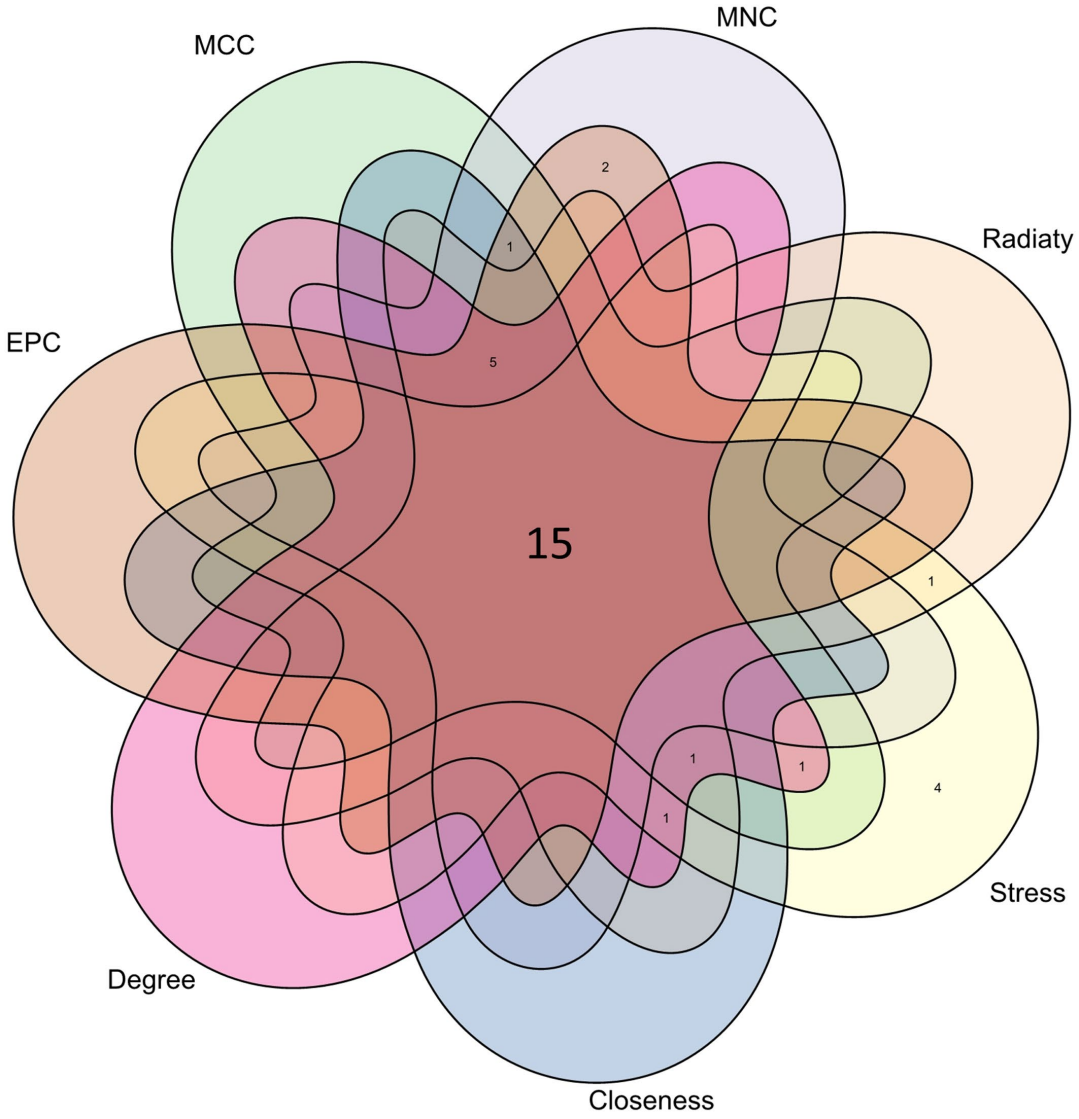
The Venn diagram shows that 7 algorithms screen out 15 overlapping hub genes.

From the PPI network in CytoHubba, a plugin for Cytoscape software, we selected the top 23 hub genes using seven algorithms. Through intersection using a Venn diagram, we ultimately identified 15 common hub genes, including ACE, ALDH1L, CYP1A1, PYGL, KLF5, NNMT, PHGDH, IDO1, EME1, CD52, MYBL2, CDC25A, BCL6, CD3D, and ESM1 (Figure 7). These hub genes may be potential biomarkers and common molecular mechanisms of pathogenesis in patients with sarcopenia and COVID-19, which may guide new therapeutic strategies for disease research.

### Functional enrichment analysis of hub genes

**Figure 8:**
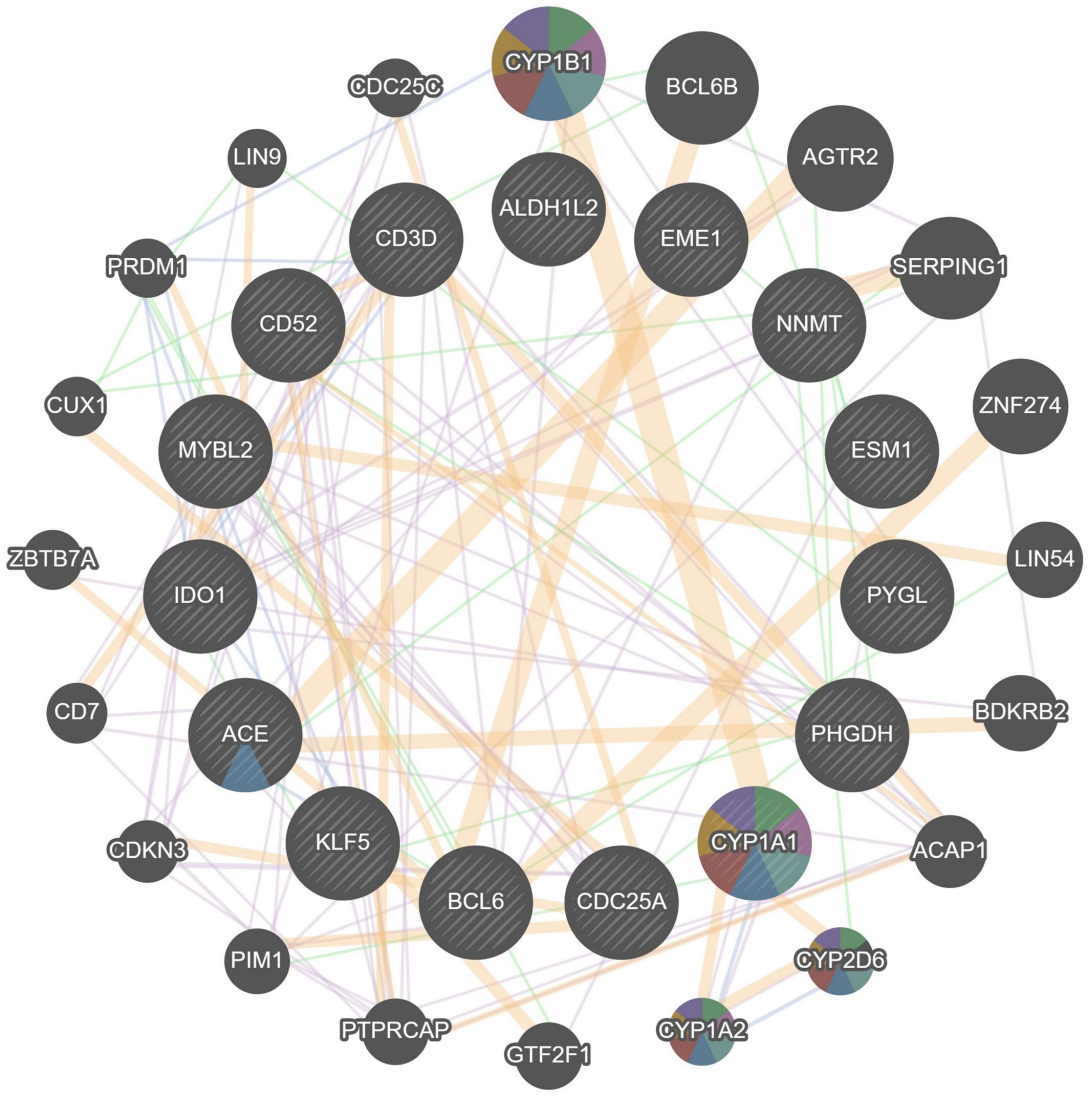
Analyze hub genes and their co-expressed genes by GeneMANIA.

Based on GeneMANIA database, we constructed an interaction network of common hub genes and their related genes to decipher the biological functions and predictive values of these hub genes, with Co-expression of 65.97%, Co-localization of 19.71%, Predicted of 12.57%, and Genetic Interactions was 1.75%. The GeneMANIA results also indicated that the functions of common hub genes and their related genes (CYP1B1, BCL6B, AGTR2, SERPING1, ZNF274 LIN54, BDKRB2, ACAP1, CYP2D6, etc.) were mainly related to the metabolic process of retinoids, steroid hydroxylase activity, protol metabolic processes, hormone metabolic processes, long-chain fatty acid metabolic processes, cytokinetic hormone metabolic processes, and monooxygenase activity (Figure 8).

**Figure 9:**
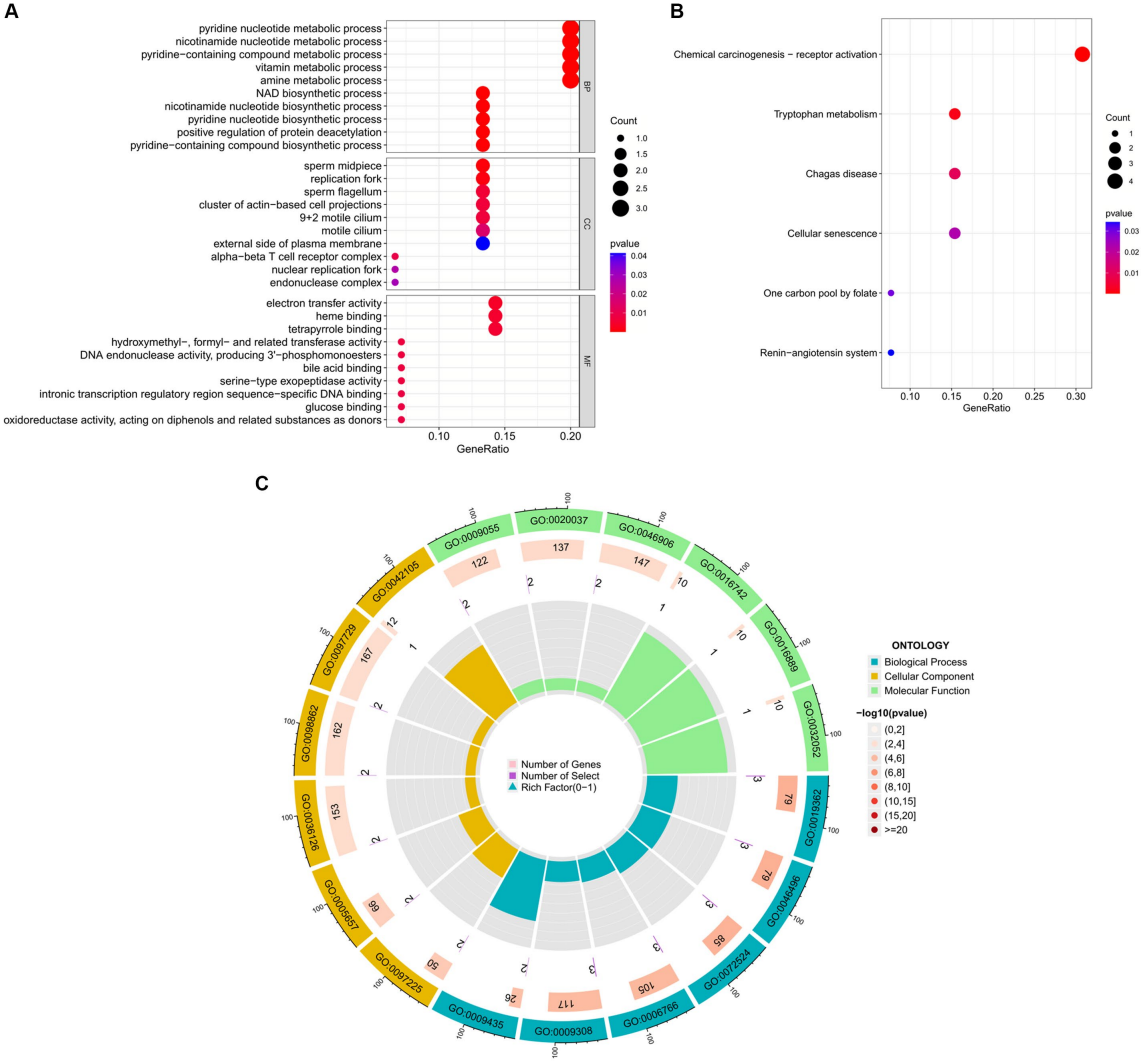
Functional enrichment analysis of Hub gene. **(A)** Bubble plot of gene ontology (GO) enrichment analysis of the Hub gene, including biological process (BP), cellular component (CC), and molecular function (MF). **(B)** Bubble plot of enrichment analysis of hub genes by the Kyoto Encyclopedia of Genes and Genomics (KEGG). **(C)** Circle plot of GO enrichment analysis.

To further explore the biological functions and signaling pathways associated with the hub genes involved in sarcopenia and COVID-19, we performed gene ontology (GO) and Kyoto Encyclopedia of Genes and Genomes (KEGG) analyses. GO analysis predicted the functional roles of the genes in terms of biological processes, cellular components and molecular functions, and the results showed (Figures 9A,C) that hub genes were enriched in several biological processes (BP), including pyridine nucleotide metabolism, nicotinamide nucleotide metabolism, pyridine compounds metabolism, vitamins metabolism, amine metabolism, and NAD biosynthesis; with regard to cellular component (CC), hub genes are mainly associated with sperm midpiece, replication fork, sperm flagellum, actin-based cell protrusion, active cilia, and T cell receptor complex. In MF it mainly includes electron transfer activity, heme binding, tetrapyrrole binding, hydroxymethyl-formyl- and related transferase activity, DNA endonuclease activity and bile acid binding. As shown in Figures 6, 9B KEGG pathways were significantly associated with sarcopenia and COVID-19 common hub genes: chemical oncogenic-receptor activation of tryptophan metabolism, Chagas disease, cellular senescence, One carbon pool by folate, and renin-angiotensin system.

### Determination of regulatory signatures

**Figure 10:**
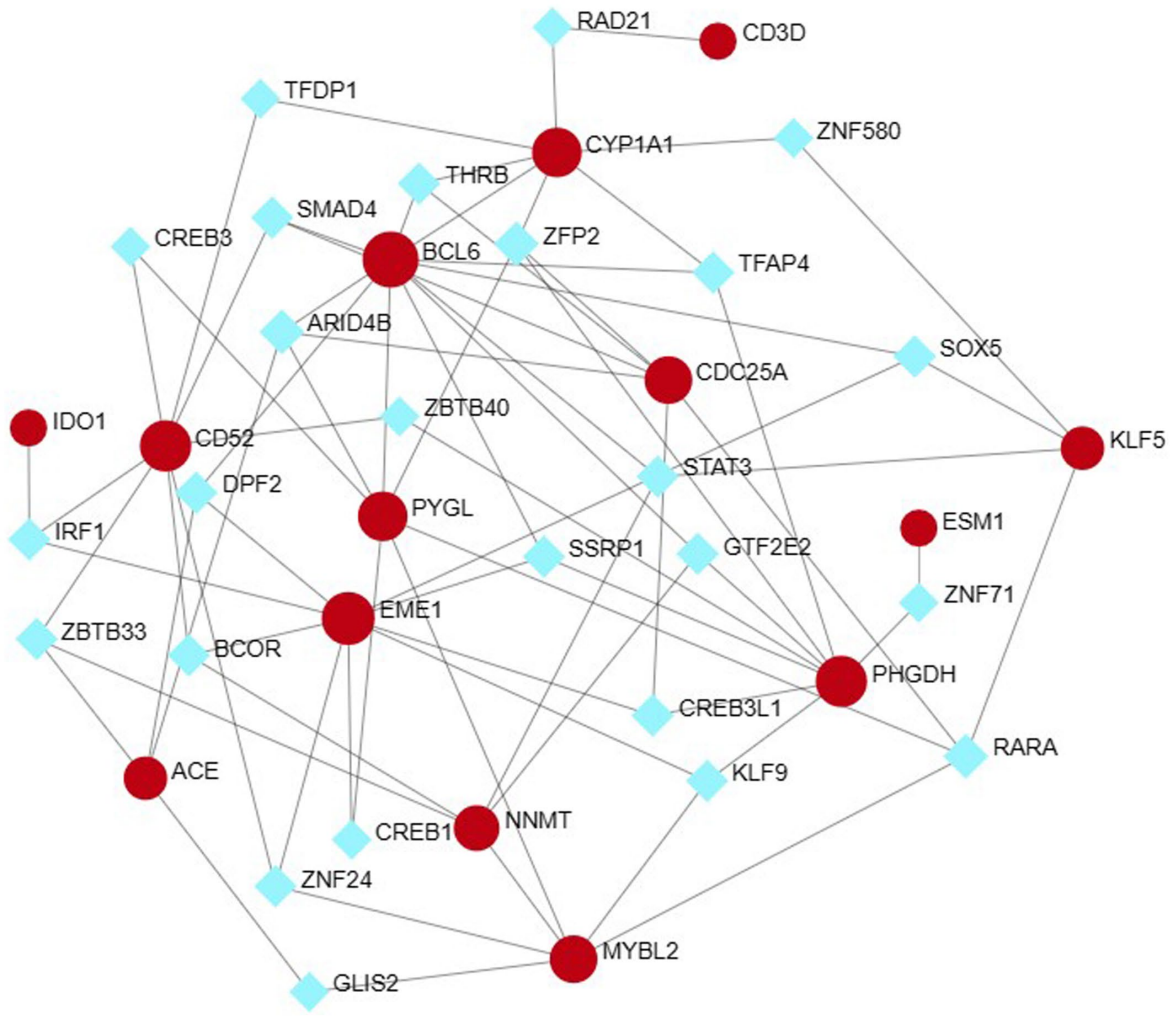
Hub-TF gene regulatory interaction network. Square nodes represent TF and round nodes represent genes.

The NetworkAnalyst network tool was used to predict and generate TF and miRNAs separately for 15 hub genes, and to construct TF-gene and miRNA-gene interaction network. The TF-gene network (Figure 10) contained 63 nodes, 140 edges and 15 genes. Among them, BCL6, ACE and EME1 genes were regulated by 17, 16 and 14 TF genes, respectively, and the transcription factor FOXC1 was closely associated with 10 genes (CYP1A1, KLF5, NNMT, PHGDH, EME1, CD52, CDC25A, BCL6, CD3D, ESM1), and these transcription factors may be important molecules that regulate the expression levels of related genes at the same time.

**Figure 11:**
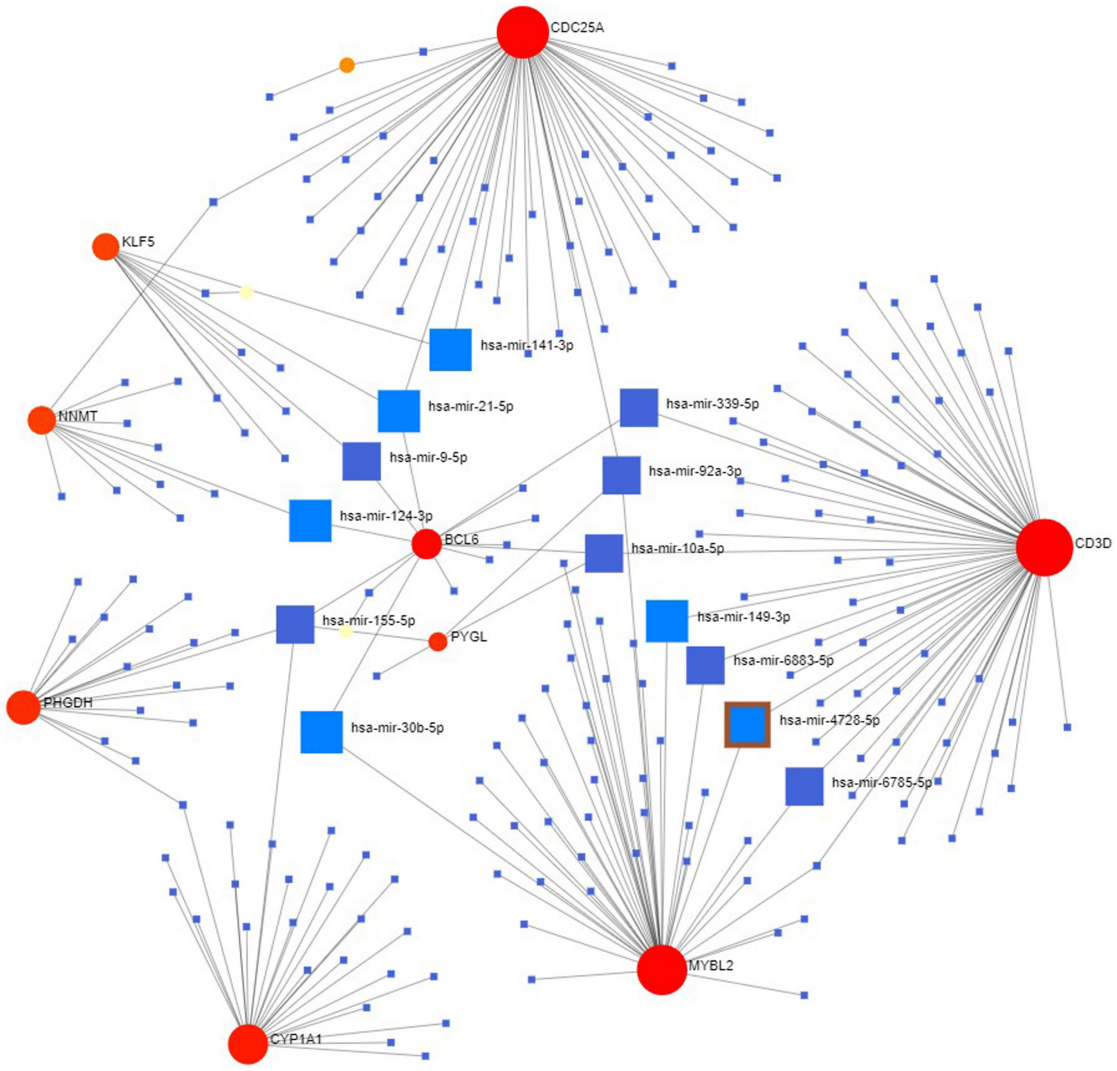
miRNA-hub gene regulatory interaction network. Square nodes represent miRNAs and round nodes represent genes.

Finally, we explored the upstream miRNAs that may regulate the expression levels of these genes and constructed the regulatory network of these 15 genes and all miRNAs (Figure 11), with a total of 231 nodes, 242 edges and 12 genes. The results showed that the miRNA (hsa-mir-155-5p) was associated with four genes (CYP1A1, PHGDH, BCL6, PYGL) at the same time, while has-mir-21-5p, has-mir-92a-3p and has-mir-10a-5p were associated with three genes at the same time. In the future, we can intervene in the expression of these upstream to affect their downstream genes to further study and control the disease progression.

### Identification of disease association

**Figure 12:**
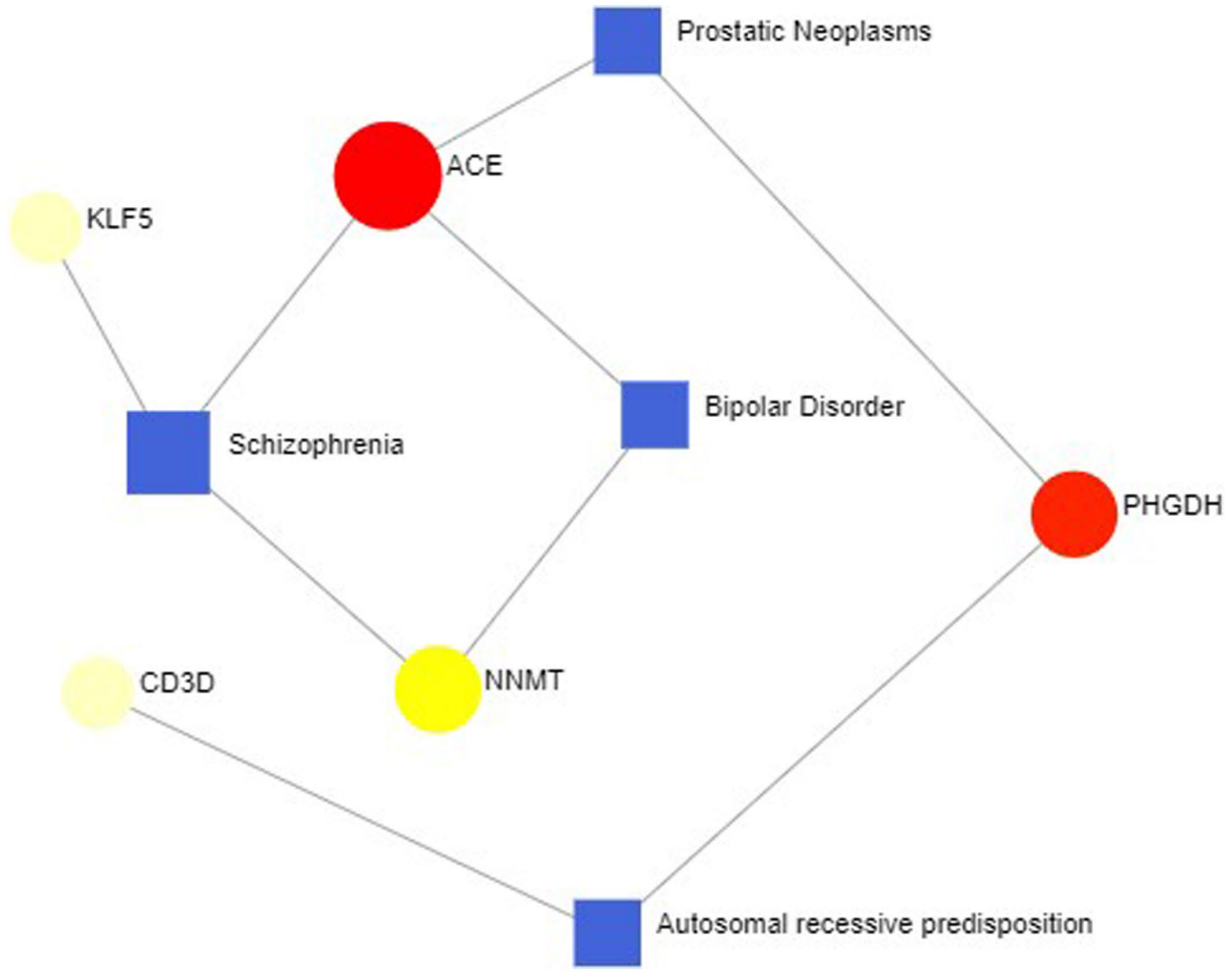
Gene-disease association network represent diseases associated with hub genes. Square nodes represent diseases, and round nodes represent genes.

In some cases, different diseases can be linked or related, such as when they share one or more similar genes. Therapeutic design strategies for diseases open the door to revealing the relationship between genes and diseases. Through Networkanalyst’s analysis of gene-disease associations, we found that schizophrenia, bipolar disorder, prostate tumors, and autosomal recessive susceptibility were most associated with our hub genes. The gene-disease associations are shown in Figure 12.

### Immune cell infiltration and correlation analysis

**Figure 13:**
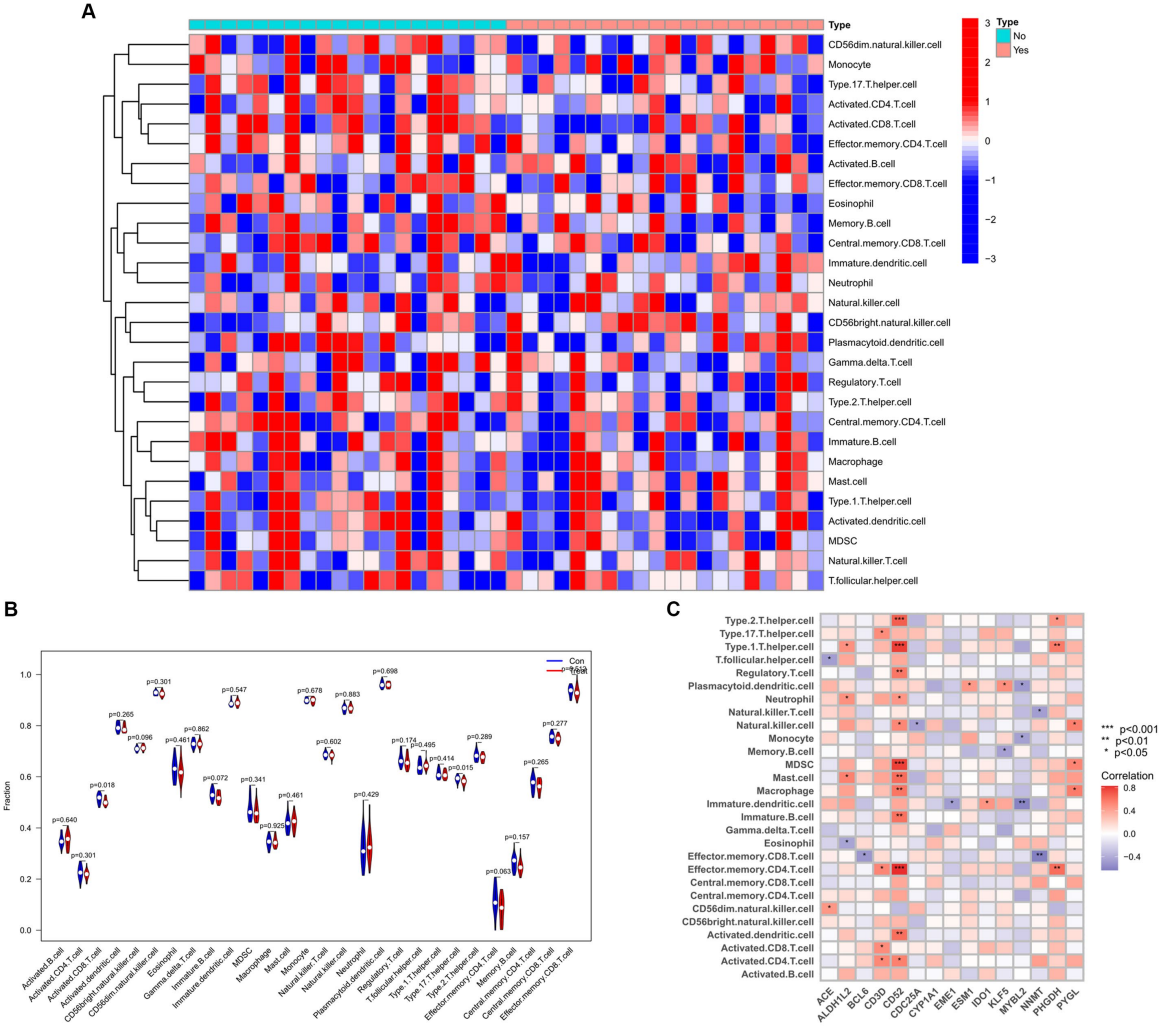
Infiltration analysis and correlation analysis of immune cells in the sarcopenia group and healthy controls group. **(A)** Heat map of immune cell subsets in the sarcopenia dataset. **(B)** Violin diagram of immune cell subsets in the sarcopenia dataset. **(C)** Correlation of immune cell subsets with common key genes. *p* < 0.05 indicates statistical difference.

To explore the relationship between the immune system and the co-occurrence of sarcopenia and COVID-19, immune infiltration analysis was performed on the sarcopenia and COVID-19 datasets. Figures A and B show the degree of infiltration of different immune cells in the sarcopenia dataset. The relationship between common key genes and immune cells was analyzed and visualized in Figure 13C. The sarcopenia group had lower expression scores of Activated.CD8.T.cell (*p* = 0.018) and Type.17.T.helper.cell (*p* = 0.015) compared to healthy controls, however, the proportion of other immune cell subsets did not differ significantly between the two groups. Subsequently, we evaluated the correlation between the expression of common hub genes and the level of infiltration of different cell subpopulations, and showed that CD52 had a strong positive correlation with Type.2.T.helper.cell, Type.1.T.helper.cell, MDSC, and Effector.memory.CD4.T.cell cells (*p* < 0.0001), whereas PHGDH had a strong correlation with Effector.memory.CD4.T.cell and Type.1.T.helper.cell cells (*p* < 0.001). In contrast, MYBL2 was negatively correlated with Plasmacytoid.dendritic.cell, Monocyte and Immature.dendritic.cell (*p* < 0.01). Similarly, the immunoinfiltration results of the COVID-19 dataset are shown in Supplementary Figure S1.

### Identification of candidate drugs

**Figure 14:**
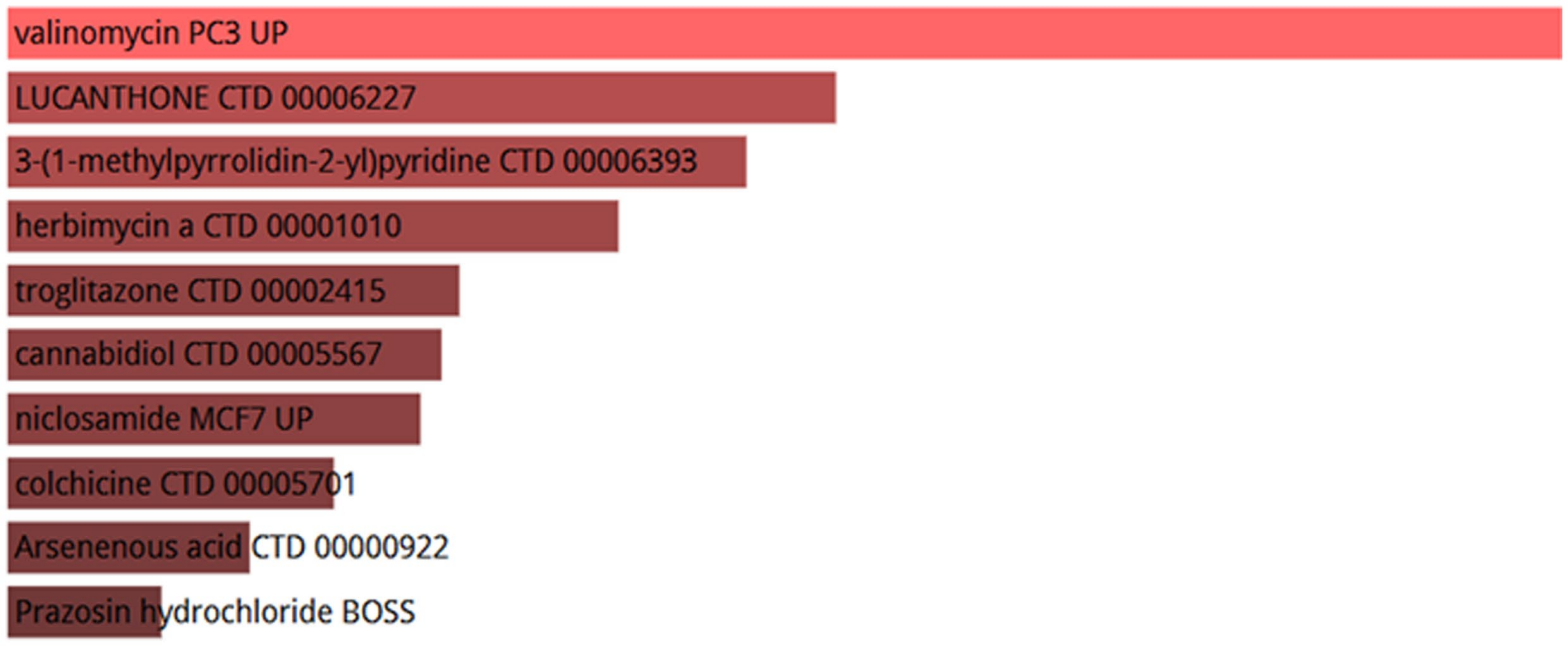
List of top 10 drugs recommended for COVID-19 and sarcopenia patients.

Ten potential therapeutic small molecule drugs were identified using Enrichr based on transcriptional characterization of the DSigDB database, which represent possible common drugs used for sarcopenia and COVID-19. The results of potential small molecules were generated based on their *p*-values to indicate the proximity between the small molecule and the gene. Figure 14 show the top 10 enriched drugs (valinomycin PC3 UP, LUCANTHONE CTD 00006227, 3-(1-methylpyrrolidin-2-yl) pyridine CTD 00006393, herbimycin a CTD 00001010, troglitazone CTD 00002415, cannabidiol CTD 00005567, niclosamide MCF7 UP, colchicine CTD 00005701, Arsenenous acid CTD 00000922, Prazosin hydrochloride BOSS).

### ROC curves of hub genes

**Figure 15:**
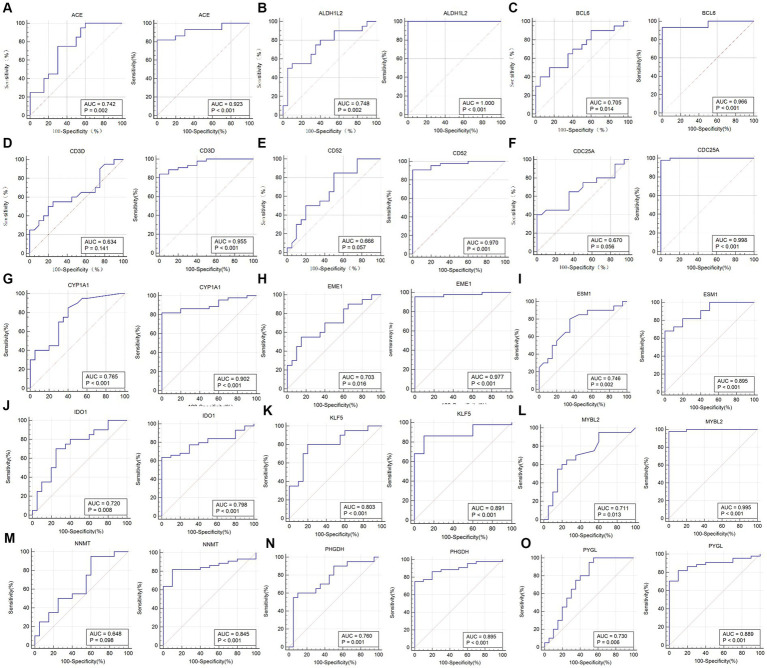
Validation of the common hub gene for diagnosis in the sarcopenia (GSE111016) dataset and the COVID-19 infected patient (GSE171110) dataset. **(A)** ACE, **(B)** ALDHIL2, **(C)** BCL6, **(D)** CD3D, **(E)** CD52, **(F)** CDC25A, **(G)** CYP1A1, **(H)** EME1, **(I)** ESM1, **(J)** IDO1, **(K)** KLF5, **(L)** MYBL2, **(M)** NNMT, **(N)** PHGDH, and **(O)** PYGL.

ROC curves were plotted to assess the diagnostic efficacy of 15 key genes (Figure 15). ACE (AUC: 0.923), CYP1A1 (AUC: 0.902), EME1 (AUC: 0.977), CD52 (AUC: 0.970), MYBL2 (AUC: 0.995), CDC25A (AUC: 0.998), BCL6 (AUC: 0.966) and CD3D (AUC: 0.955) showed relatively good diagnostic efficiency in distinguishing COVID-19 patients from healthy controls. While in the sarcopenia dataset, ACE (AUC: 0.742), ALDH1L2 (AUC: 0.748), CYP1A1 (AUC: 0.765), PYGL (AUC: 0.73), KLF5 (AUC: 0.803), PHGDH (AUC: 0.76), IDO1 (AUC: 0.72), EME1 (AUC: 0.703), MYBL2 (AUC: 0.711), BCL6 (AUC: 0.705), and ESM1 (AUC: 0.746) demonstrated better diagnostic performance for distinguishing sarcopenia from healthy individuals. Specifically, ALDH1L2 (AUC: 1) showed the best diagnostic efficiency for differentiation in the COVID-19 dataset, whereas KLF5 (AUC: 0.803) demonstrated the best discriminatory ability in the sarcopenia dataset.

## Discussion

There is evidence that patients with sarcopenia have a higher prevalence and worse prognosis after COVID-19 infection (23). COVID-19 infection can cause pathologic changes in multiple organs, including the musculoskeletal system (51), which may be associated with certain mechanisms of inflammation, immune response, and metabolic stress (52). Therefore, we sought to explore the common functions and pathways between COVID-19 and sarcopenia and to determine the interrelationship between COVID-19 and sarcopenaia. In this study, 66 common DEGs and 15 key genes (ACE, ALDH1L, CYP1A1, PYGL, KLF5, NNMT, PHGDH, IDO1, EME1, CD52, MYBL2, CDC25A, BCL6, CD3D, and ESM1) have been identified.

Four of these genes (ACE, KLF5, IDO1, and CDC25A) have been reported to be associated with the pathological mechanisms of COVID-19 and sarcopenia. Angiotensin-converting enzyme (ACE) is a chloride- and zinc-dependent peptidyl-carboxypeptidase that hydrolyzes AngI (angiotensin I) to AngII and serves as a biologically active component of the renin-angiotensin system (RAS) and the kinin-releasing enzyme-kinin system (KKS) (53, 54). ACE has been a drug target for screening against cardiovascular diseases such as hypertension and heart failure (55), and inhibition of ACE activity can prevents mitochondrial decline, improves endothelial function and muscle metabolism, and thus plays an important role in water-electrolyte homeostasis, blood pressure regulation, cardiovascular system development and vascular remodeling (53, 56). Meanwhile, it has been reported that SARS-CoV-2 virus has a strong affinity for angiotensin-converting enzyme-2 (ACE2) receptor (57). The coronavirus type 2 spiking proteins bind to cells via angiotensin-converting enzyme 2 (ACE2) receptors, leading to fusion of the viral envelope with the cell membrane and allowing viral genetic material to enter the cell where ACE2 receptors are prevalent throughout the body, leading to a wide range of tissue damage (58). KLF5 is a key zinc finger transcriptional regulator mediating muscle atrophy and is upregulated in atrophied myotubes (59, 60). It can play a key role in the development of muscle atrophy *in vitro* and *in vivo* by controlling lipid metabolism in mature skeletal muscle (61) and regulating muscle differentiation in adult myoblasts (62). It has been reported that KLF5 can also physically interacts with the transcription factor Foxo1 and cooperates with it to control the transcription of Fbxo32 (63). IDO1 (indoleamine-2,3-dioxygenase) is a cofactor-binding, redox-sensitive protein that converts tryptophan to kynurenine (Kyn) (64). Some studies have reported that inflammatory cytokines such as interferon-gamma induce IDO1 production, which leads to catabolism to produce kynurenine. Kyn levels increase with age, which can lead to muscle atrophy and bone marrow stem cells aging, and are closely associated with diseases such as sarcopenia and osteoporosis (65). Meanwhile, IDO1, as an immunomodulatory enzyme that enhances cellular immune escape, has also been significantly associated with inflammatory neointima formation (66). Coronaviruses (CoV) can activate AhR and establish infection through the IDO1-kynurenine-AhR signaling pathway (67). Recent histologic studies have shown that indoleamine 2,3-dioxygenase (IDO) is differentially expressed in the pulmonary vasculature in patients with COVID-19, and that IDO1 is predominantly present in lung tissues of patients with early/mild pneumonitis and those suffering from prolonged pneumonia (68). CDC25A (Cell Division Cycle-25A) plays a crucial role in the cell cycle and apoptosis by dephosphorylating its substrates (69). mRNA expression of CDC25A has been reported to be down-regulated in aging skeletal muscle (70) and up-regulated in COVID-19 (71). In COVID-19, CDC25A has been found to be closely associated with immune cell infiltration such as plasma cells, macrophages, T cells, dendritic cells and NK cells, and plays an important role in disease progression as a biomarker for COVID-19 diagnosis (71, 72). It has been demonstrated that MYBL2 and BCL6 are significantly upregulated in SARS-CoV-2 infected patients (73, 74). CYP1A1 is a key enzyme mediating the metabolism of broad-spectrum xenobiotics and endogenous elements, and is expressed predominantly in the peripheral airway epithelium (75). CYP1A1 has been extensively studied in pneumonia, and an association between CYP1A1 polymorphisms and the risk of pneumonia has been reported (76). The role of the remaining eight key genes (ALDH1L, EME1, PYGL, NNMT, PHGDH, CD52, CD3D, and ESM1) in COVID-19 and sarcopenia has been less studied, emphasizing their importance in future research.

In our study, GO enrichment analysis revealed that these hub genes are mainly associated with biological processes involved in energy and nucleotide metabolism. This is consistent with earlier studies that dysfunctional mitochondria play a key role in the progression of sarcopenia (77), associated with decreased respiration and increased oxidative stress (78). KEGG analysis suggests that chemical oncogenic-receptor activation and tryptophan metabolic signaling pathways are common pathogenic mechanisms in COVID-19 and sarcopenia. Tryptophan uses two metabolic pathways in humans, kynurenine and serotonin, and the imbalance in the synthesis of itself and its metabolites can lead to the occurrence of various neuropsychiatric disorders (79). In our study, disease-gene association analysis confirmed that these 15 hub genes were most associated with schizophrenia, bipolar disorder, prostate tumors, and autosomal recessive susceptibility. This finding is consistent with previous evidence that dementia and depression have been significantly associated with sarcopenia (80).

TFs and miRNAs regulate gene expression in transcription and post-transcription, respectively, and the results suggest that the transcription factor FOXC1 and miRNA (hsa-mir-155-5p) may be common molecules that simultaneously regulate the expression of these hub genes. FOXC1 is an important member of the FOX family of transcription factors, and several studies have reported that it is an important TF for COVID-19 (81, 82). And microRNA-155-5p is significantly upregulated in the acute phase of COVID-19, which promotes its immune-inflammatory response (83, 84), thus establishing its association with disease prognosis and playing an important role as a useful biomarker for monitoring and diagnosing COVID-19 disease (85). It suggests that in the future, we can intervene in its expression to regulate the gene and further study and control the disease.

In this study, we further analyzed the infiltration of immune cells in different diseases and the correlation between hub genes and immune factors. The results revealed that the sarcopenia group had lower expression scores of Activated.CD8.T.cells (*p* = 0.018) and Type.17.T.helper.cells (*p* = 0.015) compared with the healthy controls group. CD52 had a strong positive correlation (*p* < 0.0001) with Type.2.T.helper.cells, Type.1.T.helper. Cells and MDSC cells, whereas MYBL2 was negatively correlated with Plasmacytoid.dendritic.cell, Monocyte and Immature.dendritic.cell (*p* < 0.01). It was previously proposed that the immune system regulates muscle regeneration and growth and plays an important role in the progression of sarcopenia (86). These immune cells, including lymphocytes, macrophages, neutrophils and other immune cells, work together to alter the condition of muscle fibers, leading to loss of muscle strength and muscle mass (87). It has also been found that aging of the immune system leads to a reduction in muscle stem cell populations, promoting their transition to a fibrotic phenotype, which regulates sarcopenia (88). This suggests the importance of different levels of immune cell infiltration for COVID-19 and sarcopenia. Several chemical agents and drugs have been used as potential therapeutic targets against COVID-19 or sarcopenia. However, to date, no drugs have been identified to treat individuals with both COVID-19 and sarcopenia. In our study, we explored 10 drugs that could be used as possible targets. The results showed that valinomycin PC3 UP is the best candidate for the treatment of sarcopenia and COVID-19.

Although some previous studies have reported the relationship between COVID-19 or sarcopenia and the hub gene, but the common molecular mechanisms between the two have not been explored by bioinformatics approaches. In this study, we explored and identified the common DEG and hub genes of COVID-19 and sarcopenia for the first time, which may help to further elucidate the common pathogenesis of both. However, there are some limitations of our study. First, the data were downloaded from public databases, and the amount of data and information was limited and unbalanced. In addition, even though differential and enrichment analyses were performed for sarcopenia and COVID-19, key genes driving disease progression may still be missed. Finally, the pathological causal mechanism of diseases caused by HUB gene and immune infiltration require external experiments to further validate our findings.

Overall, we explored the link between sarcopenia and COVID-19 using transcriptomic data analysis, further identified the common DEG and hub genes for sarcopenia and COVID-19, and performed several bioinformatics analyses based on them. It was found sarcopenia and COVID-19 share some common pathogenic mechanisms, which may be mediated by specific key genes. This study provides new biological targets and ideas for further investigation of molecular mechanisms, search for new drugs, and early diagnosis and effective treatment for patients with sarcopenia and COVID-19. However, the biological significance of these results needs to be further explored through *in vitro* and *in vivo* experiments.

## Data availability statement

Publicly available datasets were analyzed in this study. This data can be found at: https://www.ncbi.nlm.nih.gov/geo/, GSE111016 and GSE171110.

## Author contributions

JZ: Conceptualization, Data curation, Formal analysis, Funding acquisition, Investigation, Methodology, Project administration, Resources, Software, Supervision, Validation, Visualization, Writing – original draft, Writing – review & editing. HuY: Conceptualization, Data curation, Formal analysis, Funding acquisition, Investigation, Methodology, Project administration, Resources, Software, Supervision, Validation, Visualization, Writing – original draft, Writing – review & editing. JY: Conceptualization, Data curation, Formal analysis, Funding acquisition, Investigation, Methodology, Project administration, Resources, Software, Supervision, Validation, Visualization, Writing – original draft, Writing – review & editing. YD: Conceptualization, Data curation, Formal analysis, Investigation, Methodology, Project administration, Validation, Writing – original draft. ZheL: Conceptualization, Data curation, Formal analysis, Methodology, Project administration, Resources, Supervision, Validation, Visualization, Writing – review & editing. XL: Conceptualization, Data curation, Formal analysis, Methodology, Project administration, Validation, Writing – original draft. HaY: Data curation, Formal analysis, Methodology, Project administration, Supervision, Validation, Writing – original draft. ZhW: Conceptualization, Data curation, Investigation, Methodology, Writing – original draft. ZiW: Conceptualization, Data curation, Investigation, Methodology, Writing – original draft. LJ: Conceptualization, Data curation, Software, Supervision, Writing – original draft. ZR: Conceptualization, Data curation, Formal analysis, Investigation, Writing – original draft. HL: Data curation, Methodology, Project administration, Resources, Writing – original draft. ZhoL: Conceptualization, Data curation, Formal analysis, Funding acquisition, Investigation, Methodology, Project administration, Resources, Software, Supervision, Validation, Visualization, Writing – review & editing. YL: Conceptualization, Data curation, Formal analysis, Funding acquisition, Investigation, Methodology, Project administration, Resources, Software, Supervision, Validation, Visualization, Writing – review & editing.

## Footnotes

^1^ https://covid19.who.int/

^2^ https://www.ncbi.nlm.nih.gov/geo/

^3^ http://jvenn.toulouse.inra.fr/app/example.html

^4^ https://maayanlab.cloud/Enrichr/

^5^ http://string-db.org

^6^ http://apps.cytoscape.org/apps/cytohubba

^7^ https://genemania.org

^8^ https://amp.Pharm.mssm.Edu/Enrichr./

## References

[1] Cruz-Jentoft AJ. Sarcopenia, the last organ insufficiency. European Geriatric Med. (2016) 7:195–6. doi: 10.1016/j.eurger.2016.01.003

[2] Cruz-Jentoft AJ Sayer AA. Sarcopenia. Lancet. (2019) 393:2636–46. doi: 10.1016/s0140-6736(19)31138-9 31171417

[3] Damluji AA Alfaraidhy M AlHajri N Rohant NN Kumar M Al Malouf C. Sarcopenia and cardiovascular diseases. Circulation. (2023) 147:1534–53. doi: 10.1161/CIRCULATIONAHA.123.064071, PMID: 37186680 PMC10180053

[4] Cruz-Jentoft AJ Bahat G Bauer J Boirie Y Bruyère O Cederholm T. Sarcopenia: revised European consensus on definition and diagnosis. Age Ageing. (2019) 48:16–31. doi: 10.1093/ageing/afy169, PMID: 30312372 PMC6322506

[5] Sayer AA Cruz-Jentoft A. Sarcopenia definition, diagnosis and treatment: consensus is growing. Age Ageing. (2022) 51:10. doi: 10.1093/ageing/afac220, PMID: 36273495 PMC9588427

[6] Rosenberg IH. Sarcopenia: origins and clinical relevance. J Nutr. (1997) 127:990S–1S. doi: 10.1093/jn/127.5.990S, PMID: 9164280

[7] Dhillon RJS Hasni S. Pathogenesis and Management of Sarcopenia. Clin Geriatr Med. (2017) 33:17–26. doi: 10.1016/j.cger.2016.08.002, PMID: 27886695 PMC5127276

[8] Chen LK Liu LK Woo J Assantachai P Auyeung TW Bahyah KS. Sarcopenia in Asia: consensus report of the Asian working Group for Sarcopenia. J Am Med Dir Assoc. (2014) 15:95–101. doi: 10.1016/j.jamda.2013.11.025 24461239

[9] Yuan S Larsson SC. Epidemiology of sarcopenia: prevalence, risk factors, and consequences. Metabolism. (2023) 144:155533. doi: 10.1016/j.metabol.2023.155533, PMID: 36907247

[10] Petermann-Rocha F Balntzi V Gray SR Lara J Ho FK Pell JP. Global prevalence of sarcopenia and severe sarcopenia: a systematic review and meta-analysis. J Cachexia Sarcopenia Muscle. (2021) 13:86–99. doi: 10.1002/jcsm.12783, PMID: 34816624 PMC8818604

[11] Verdijk LB Snijders T Drost M Delhaas T Kadi F van Loon LJC. Satellite cells in human skeletal muscle; from birth to old age. Age. (2013) 36:545–57. doi: 10.1007/s11357-013-9583-2, PMID: 24122288 PMC4039250

[12] Ciciliot S Rossi AC Dyar KA Blaauw B Schiaffino S. Muscle type and fiber type specificity in muscle wasting. Int J Biochem Cell Biol. (2013) 45:2191–9. doi: 10.1016/j.biocel.2013.05.016, PMID: 23702032

[13] Ferri E Marzetti E Calvani R Picca A Cesari M Arosio B. Role of age-related mitochondrial dysfunction in sarcopenia. Int J Mol Sci. (2020) 21:15. doi: 10.3390/ijms21155236, PMID: 32718064 PMC7432902

[14] Manini TM Hong SL Clark BC. Aging and muscle. Curr Opin Clin Nutr Metab Care. (2013) 16:21–6. doi: 10.1097/MCO.0b013e32835b5880, PMID: 23222705 PMC3868452

[15] Fielding RA Vellas B Evans WJ Bhasin S Morley JE Newman AB. Sarcopenia: an undiagnosed condition in older adults. Current consensus definition: prevalence, etiology, and consequences. International working group on sarcopenia. J Am Med Dir Assoc. (2011) 12:249–56. doi: 10.1016/j.jamda.2011.01.003, PMID: 21527165 PMC3377163

[16] Hunter GR Singh H Carter SJ Bryan DR Fisher G. Sarcopenia and its implications for metabolic health. J Obes. (2019) 2019:1–10. doi: 10.1155/2019/8031705, PMID: 30956817 PMC6431367

[17] Chen L-K Woo J Assantachai P Auyeung TW Chou MY Iijima K. Asian working Group for Sarcopenia: 2019 consensus update on sarcopenia diagnosis and treatment. J Am Med Dir Assoc. (2020) 21:300–7.e2. doi: 10.1016/j.jamda.2019.12.012, PMID: 32033882

[18] Rothan HA Byrareddy SN. The epidemiology and pathogenesis of coronavirus disease (COVID-19) outbreak. J Autoimmun. (2020) 109:102433. doi: 10.1016/j.jaut.2020.102433, PMID: 32113704 PMC7127067

[19] Zhou F Yu T du R Fan G Liu Y Liu Z. Clinical course and risk factors for mortality of adult inpatients with COVID-19 in Wuhan, China: a retrospective cohort study. Lancet. (2020) 395:1054–62. doi: 10.1016/s0140-6736(20)30566-3, PMID: 32171076 PMC7270627

[20] de Lusignan S Dorward J Correa A Jones N Akinyemi O Amirthalingam G. Risk factors for SARS-CoV-2 among patients in the Oxford Royal College of general practitioners research and surveillance Centre primary care network: a cross-sectional study. Lancet Infect Dis. (2020) 20:1034–42. doi: 10.1016/s1473-3099(20)30371-6, PMID: 32422204 PMC7228715

[21] Bailey C Black JRM Swanton C. Cancer research: the lessons to learn from COVID-19. Cancer Discov. (2020) 10:1263–6. doi: 10.1158/2159-8290.Cd-20-0823, PMID: 32669285

[22] Jordan RE Adab P. Who is most likely to be infected with SARS-CoV-2? Lancet Infect Dis. (2020) 20:995–6. doi: 10.1016/s1473-3099(20)30395-9, PMID: 32422197 PMC7228712

[23] Wang P-y Li Y Wang Q. Sarcopenia: an underlying treatment target during the COVID-19 pandemic. Nutrition. (2021) 84:111104. doi: 10.1016/j.nut.2020.111104, PMID: 33421827 PMC7833321

[24] Meftahi GH Jangravi Z Sahraei H Bahari Z. The possible pathophysiology mechanism of cytokine storm in elderly adults with COVID-19 infection: the contribution of “inflame-aging”. Inflamm Res. (2020) 69:825–39. doi: 10.1007/s00011-020-01372-8, PMID: 32529477 PMC7289226

[25] Nelke C Dziewas R Minnerup J Meuth SG Ruck T. Skeletal muscle as potential central link between sarcopenia and immune senescence. EBioMedicine. (2019) 49:381–8. doi: 10.1016/j.ebiom.2019.10.034, PMID: 31662290 PMC6945275

[26] Giudice J Taylor JM. Muscle as a paracrine and endocrine organ. Curr Opin Pharmacol. (2017) 34:49–55. doi: 10.1016/j.coph.2017.05.005 28605657 PMC5808999

[27] Bano G Trevisan C Carraro S Solmi M Luchini C Stubbs B. Inflammation and sarcopenia: a systematic review and meta-analysis. Maturitas. (2017) 96:10–5. doi: 10.1016/j.maturitas.2016.11.006 28041587

[28] Akseer N Kandru G Keats EC Bhutta ZA. COVID-19 pandemic and mitigation strategies: implications for maternal and child health and nutrition. Am J Clin Nutr. (2020) 112:251–6. doi: 10.1093/ajcn/nqaa171, PMID: 32559276 PMC7337702

[29] Jackson TA Wilson D Masud T Greig C Welch C. COVID-19 and acute sarcopenia. Aging Dis. (2020) 11:1345–51. doi: 10.14336/ad.2020.1014, PMID: 33269092 PMC7673845

[30] Raveendran AV Misra A. Post COVID-19 syndrome (“long COVID”) and diabetes: challenges in diagnosis and management. Diabetes Metab Syndr Clin Res Rev. (2021) 15:102235. doi: 10.1016/j.dsx.2021.102235, PMID: 34384972 PMC8317446

[31] Patra BG Maroufy V Soltanalizadeh B Deng N Zheng WJ Roberts K. A content-based literature recommendation system for datasets to improve data reusability – a case study on gene expression omnibus (GEO) datasets. J Biomed Inform. (2020) 104:103399. doi: 10.1016/j.jbi.2020.103399, PMID: 32151769

[32] Migliavacca E Tay SKH Patel HP Sonntag T Civiletto G McFarlane C. Mitochondrial oxidative capacity and NAD+ biosynthesis are reduced in human sarcopenia across ethnicities. Nat Commun. (2019) 10:5808. doi: 10.1038/s41467-019-13694-1, PMID: 31862890 PMC6925228

[33] Lévy Y Wiedemann A Hejblum BP Durand M Lefebvre C Surénaud M. CD177, a specific marker of neutrophil activation, is associated with coronavirus disease 2019 severity and death. iScience. (2021) 24:102711. doi: 10.1016/j.isci.2021.102711, PMID: 34127958 PMC8189740

[34] Kuleshov MV Jones MR Rouillard AD Fernandez NF Duan Q Wang Z. Enrichr: a comprehensive gene set enrichment analysis web server 2016 update. Nucleic Acids Res. (2016) 44:W90–7. doi: 10.1093/nar/gkw377, PMID: 27141961 PMC4987924

[35] Szklarczyk D Kirsch R Koutrouli M Nastou K Mehryary F Hachilif R. The STRING database in 2023: protein–protein association networks and functional enrichment analyses for any sequenced genome of interest. Nucleic Acids Res. (2023) 51:D638–46. doi: 10.1093/nar/gkac1000, PMID: 36370105 PMC9825434

[36] Doncheva NT Morris JH Gorodkin J Jensen LJ. Cytoscape StringApp: network analysis and visualization of proteomics data. J Proteome Res. (2018) 18:623–32. doi: 10.1021/acs.jproteome.8b00702, PMID: 30450911 PMC6800166

[37] Shannon P Markiel A Ozier O Baliga NS Wang JT Ramage D. Cytoscape: a software environment for integrated models of biomolecular interaction networks. Genome Res. (2003) 13:2498–504. doi: 10.1101/gr.1239303, PMID: 14597658 PMC403769

[38] Chin C-H Chen S-H Wu H-H Ho C-W Ko M-T Lin C-Y. cytoHubba: identifying hub objects and sub-networks from complex interactome. BMC Syst Biol. (2014) 8:S11. doi: 10.1186/1752-0509-8-S4-S11 25521941 PMC4290687

[39] Franz M Rodriguez H Lopes C Zuberi K Montojo J Bader GD. GeneMANIA update 2018. Nucleic Acids Res. (2018) 46:W60–4. doi: 10.1093/nar/gky311, PMID: 29912392 PMC6030815

[40] Cai Y Yu X Hu S Yu J. A brief review on the mechanisms of miRNA regulation. Genomics Proteomics Bioinformatics. (2009) 7:147–54. doi: 10.1016/s1672-0229(08)60044-3, PMID: 20172487 PMC5054406

[41] Lambert SA Jolma A Campitelli LF das PK Yin Y Albu M. The human transcription factors. Cell. (2018) 172:650–65. doi: 10.1016/j.cell.2018.01.029 29425488 PMC12908702

[42] Zhou G Soufan O Ewald J Hancock REW Basu N Xia J. NetworkAnalyst 3.0: a visual analytics platform for comprehensive gene expression profiling and meta-analysis. Nucleic Acids Res. (2019) 47:W234–41. doi: 10.1093/nar/gkz240, PMID: 30931480 PMC6602507

[43] Huang H-Y Lin Y-C-D Li J Huang KY Shrestha S Hong HC. miRTarBase 2020: updates to the experimentally validated microRNA–target interaction database. Nucleic Acids Res. (2019) 48:D148–54. doi: 10.1093/nar/gkz896, PMID: 31647101 PMC7145596

[44] Castro-Mondragon JA Riudavets-Puig R Rauluseviciute I Berhanu Lemma R Turchi L Blanc-Mathieu R. JASPAR 2022: the 9th release of the open-access database of transcription factor binding profiles. Nucleic Acids Res. (2022) 50:D165–73. doi: 10.1093/nar/gkab1113, PMID: 34850907 PMC8728201

[45] Piñero J Bravo À Queralt-Rosinach N Gutiérrez-Sacristán A Deu-Pons J Centeno E. DisGeNET: a comprehensive platform integrating information on human disease-associated genes and variants. Nucleic Acids Res. (2017) 45:D833–9. doi: 10.1093/nar/gkw943, PMID: 27924018 PMC5210640

[46] Piñero J Ramírez-Anguita JM Saüch-Pitarch J Ronzano F Centeno E Sanz F. The DisGeNET knowledge platform for disease genomics: 2019 update. Nucleic Acids Res. (2019) 48:D845–55. doi: 10.1093/nar/gkz1021, PMID: 31680165 PMC7145631

[47] Subramanian A Tamayo P Mootha VK Mukherjee S Ebert BL Gillette MA. Gene set enrichment analysis: a knowledge-based approach for interpreting genome-wide expression profiles. Proc Natl Acad Sci USA. (2005) 102:15545–50. doi: 10.1073/pnas.0506580102, PMID: 16199517 PMC1239896

[48] Chen Y Feng Y Yan F Zhao Y Zhao H Guo Y. A novel immune-related gene signature to identify the tumor microenvironment and Prognose disease among patients with Oral squamous cell carcinoma patients using ssGSEA: a bioinformatics and biological validation study. Front Immunol. (2022) 13:922195. doi: 10.3389/fimmu.2022.922195, PMID: 35935989 PMC9351622

[49] Yoo M Shin J Kim J Ryall KA Lee K Lee S. DSigDB: drug signatures database for gene set analysis. Bioinformatics. (2015) 31:3069–71. doi: 10.1093/bioinformatics/btv313, PMID: 25990557 PMC4668778

[50] Robin X Turck N Hainard A Tiberti N Lisacek F Sanchez JC. pROC: an open-source package for R and S+ to analyze and compare ROC curves. BMC Bioinformatics. (2011) 12:77. doi: 10.1186/1471-2105-12-77, PMID: 21414208 PMC3068975

[51] Veronesi F Contartese D Martini L Visani A Fini M. Speculation on the pathophysiology of musculoskeletal injury with COVID-19 infection. Front Med. (2022) 9:930789. doi: 10.3389/fmed.2022.930789, PMID: 35911401 PMC9329661

[52] Lauwers M Au M Yuan S Wen C. COVID-19 in joint ageing and osteoarthritis: current status and perspectives. Int J Mol Sci. (2022) 23:2. doi: 10.3390/ijms23020720, PMID: 35054906 PMC8775477

[53] Khurana V Goswami B. Angiotensin converting enzyme (ACE). Clin Chim Acta. (2022) 524:113–22. doi: 10.1016/j.cca.2021.10.029 34728179

[54] Zhao Y. Structure and function of angiotensin converting enzyme and its inhibitors. Chin J Biotechnol. (2008) 24:171–6. doi: 10.1016/s1872-2075(08)60007-2, PMID: 18464595 PMC7148949

[55] Pitt B. ACE inhibitors in heart failure: prospects and limitations. Cardiovasc Drugs Ther. (1997) 11:285–90. doi: 10.1023/a:1007795915009, PMID: 9211022

[56] Sumukadas D Struthers AD McMurdo MET. Sarcopenia – a potential target for angiotensin-converting enzyme inhibition? Gerontology. (2006) 52:237–42. doi: 10.1159/000093656, PMID: 16849867

[57] Aladag E Tas Z Ozdemir BS Akbaba TH Akpınar MG Goker H. Human ace D/I polymorphism could affect the Clinicobiological course of COVID-19. Journal of the renin-angiotensin-aldosterone. System. (2021) 2021:1–7. doi: 10.1155/2021/5509280, PMID: 34603503 PMC8448604

[58] Kai H Kai M. Interactions of coronaviruses with ACE2, angiotensin II, and RAS inhibitors—lessons from available evidence and insights into COVID-19. Hypertens Res. (2020) 43:648–54. doi: 10.1038/s41440-020-0455-8, PMID: 32341442 PMC7184165

[59] McConnell BB Yang VW. Mammalian Krüppel-like factors in health and diseases. Physiol Rev. (2010) 90:1337–81. doi: 10.1152/physrev.00058.2009, PMID: 20959618 PMC2975554

[60] Prosdocimo DA Sabeh MK Jain MK. Kruppel-like factors in muscle health and disease. Trends Cardiovasc Med. (2015) 25:278–87. doi: 10.1016/j.tcm.2014.11.006, PMID: 25528994 PMC4422160

[61] Oishi Y Manabe I Tobe K Ohsugi M Kubota T Fujiu K. SUMOylation of Krüppel-like transcription factor 5 acts as a molecular switch in transcriptional programs of lipid metabolism involving PPAR-δ. Nat Med. (2008) 14:656–66. doi: 10.1038/nm1756, PMID: 18500350

[62] Hayashi S Manabe I Suzuki Y Relaix F Oishi Y. Klf5 regulates muscle differentiation by directly targeting muscle-specific genes in cooperation with MyoD in mice. eLife. (2016) 5:17462. doi: 10.7554/eLife.17462, PMID: 27743478 PMC5074804

[63] Liu L Koike H Ono T Hayashi S Kudo F Kaneda A. Identification of a KLF5-dependent program and drug development for skeletal muscle atrophy. Proc Natl Acad Sci. (2021) 118:35. doi: 10.1073/pnas.2102895118, PMID: 34426497 PMC8536343

[64] Hennes E Lampe P Dötsch L Bruning N Pulvermacher LM Sievers S. Cell-based identification of new IDO1 modulator Chemotypes. Angew Chem Int Ed. (2021) 60:9869–74. doi: 10.1002/anie.202016004, PMID: 33565680 PMC8252559

[65] Sultana S Elengickal A Bensreti H de Chantemèle EB McGee-Lawrence ME Hamrick MW. The kynurenine pathway in HIV, frailty and inflammaging. Front Immunol. (2023) 14:1244622. doi: 10.3389/fimmu.2023.1244622, PMID: 37744363 PMC10514395

[66] Mondal A Smith C DuHadaway JB Sutanto-Ward E Prendergast GC Bravo-Nuevo A. IDO1 is an integral mediator of inflammatory neovascularization. EBioMedicine. (2016) 14:74–82. doi: 10.1016/j.ebiom.2016.11.013, PMID: 27889479 PMC5161421

[67] Turski WA Wnorowski A Turski GN Turski CA Turski L. AhR and IDO1 in pathogenesis of Covid-19 and the “systemic AhR activation syndrome:” a translational review and therapeutic perspectives. Restor Neurol Neurosci. (2020) 38:343–54. doi: 10.3233/rnn-201042, PMID: 32597823 PMC7592680

[68] Chilosi M Doglioni C Ravaglia C Martignoni G Salvagno GL Pizzolo G. Unbalanced IDO1/IDO2 endothelial expression and skewed Keynurenine pathway in the pathogenesis of COVID-19 and post-COVID-19 pneumonia. Biomedicines. (2022) 10:6. doi: 10.3390/biomedicines10061332, PMID: 35740354 PMC9220124

[69] Boutros R Lobjois V Ducommun B. CDC25 phosphatases in cancer cells: key players? Good targets? Nat Rev Cancer. (2007) 7:495–507. doi: 10.1038/nrc2169, PMID: 17568790

[70] Drummond MJ McCarthy JJ Sinha M Spratt HM Volpi E Esser KA. Aging and microRNA expression in human skeletal muscle: a microarray and bioinformatics analysis. Physiol Genomics. (2011) 43:595–603. doi: 10.1152/physiolgenomics.00148.2010, PMID: 20876843 PMC3110890

[71] Chen Z-j Xiao J Chen H-h. Identification of key genes related to immune cells in patients with COVID-19 via integrated bioinformatics-based analysis. Biochem Genet. (2023) 61:2650–71. doi: 10.1007/s10528-023-10400-1, PMID: 37222960 PMC10206360

[72] Sagulkoo P Chuntakaruk H Rungrotmongkol T Suratanee A Plaimas K. Multi-level biological network analysis and drug repurposing based on leukocyte transcriptomics in severe COVID-19: in silico systems biology to precision medicine. J Personal Med. (2022) 12:7. doi: 10.3390/jpm12071030, PMID: 35887528 PMC9319133

[73] Khalid Z Huan M Sohail Raza M Abbas M Naz Z Kombe Kombe AJ. Identification of novel therapeutic candidates against SARS-CoV-2 infections: an application of RNA sequencing toward mRNA based Nanotherapeutics. Front Microbiol. (2022) 13:901848. doi: 10.3389/fmicb.2022.901848, PMID: 35983322 PMC9378778

[74] Nain Z Rana HK Liò P Islam SMS Summers MA Moni MA. Pathogenetic profiling of COVID-19 and SARS-like viruses. Brief Bioinform. (2021) 22:1175–96. doi: 10.1093/bib/bbaa173, PMID: 32778874 PMC7454314

[75] Guin D Yadav S Singh P Singh P Thakran S Kukal S. Human genetic factors associated with pneumonia risk, a cue for COVID-19 susceptibility. Infect Genet Evol. (2022) 102:105299. doi: 10.1016/j.meegid.2022.105299, PMID: 35545162 PMC9080029

[76] Zhao J Zhang W Shen L Yang X Liu Y Gai Z. Association of the ACE, GSTM1, IL-6, NOS3, and CYP1A1 polymorphisms with susceptibility of *mycoplasma pneumoniae* pneumonia in Chinese children. Medicine. (2017) 96:e6642. doi: 10.1097/md.0000000000006642, PMID: 28403117 PMC5403114

[77] Hoogkamer W. Mitochondria initiate and regulate sarcopenia. Exerc Sport Sci Rev. (2017) 45:34–40. doi: 10.1249/jes.0000000000000094, PMID: 28098577 PMC5357179

[78] López-Otín C Blasco MA Partridge L Serrano M Kroemer G. Hallmarks of aging: an expanding universe. Cell. (2023) 186:243–78. doi: 10.1016/j.cell.2022.11.001, PMID: 36599349

[79] Davidson M Rashidi N Nurgali K Apostolopoulos V. The role of tryptophan metabolites in neuropsychiatric disorders. Int J Mol Sci. (2022) 23:17. doi: 10.3390/ijms23179968, PMID: 36077360 PMC9456464

[80] Mogi M Endo T Akai K Kitahara S Abe T Takeda M. An association analysis between hypertension, dementia, and depression and the phases of pre-sarcopenia to sarcopenia: a cross-sectional analysis. PLoS One. (2021) 16:e0252784. doi: 10.1371/journal.pone.0252784, PMID: 34292967 PMC8297796

[81] Islam T Rahman MR Aydin B Beklen H Arga KY Shahjaman M. Integrative transcriptomics analysis of lung epithelial cells and identification of repurposable drug candidates for COVID-19. Eur J Pharmacol. (2020) 887:173594. doi: 10.1016/j.ejphar.2020.173594 32971089 PMC7505772

[82] Li P Li T Zhang Z Dai X Zeng B Li Z. Bioinformatics and system biology approach to identify the influences among COVID-19, ARDS and sepsis. Front Immunol. (2023) 14:1152186. doi: 10.3389/fimmu.2023.1152186, PMID: 37261353 PMC10227520

[83] Gaytán-Pacheco N Ibáñez-Salazar A Herrera-van Oostdam AS Oropeza-Valdez JJ Magaña-Aquino M Adrián López J. miR-146a, miR-221, and miR-155 are involved in inflammatory immune response in severe COVID-19 patients. Diagnostics. (2022) 13:1. doi: 10.3390/diagnostics13010133, PMID: 36611425 PMC9818442

[84] Gedikbasi A Adas G Isiksacan N Kart Yasar K Canbolat Unlu E Yilmaz R. The effect of host miRNAs on prognosis in COVID-19: miRNA-155 may promote severity via targeting suppressor of cytokine signaling 1 (SOCS1) gene. Genes. (2022) 13:7. doi: 10.3390/genes13071146, PMID: 35885930 PMC9320261

[85] Li X Wang Y Zhou Q Pan J Xu J. Potential predictive value of miR-125b-5p, miR-155-5p and their target genes in the course of COVID-19. Infect Drug Resist. (2022) 15:4079–91. doi: 10.2147/idr.S372420, PMID: 35937783 PMC9346419

[86] Tidball JG. Regulation of muscle growth and regeneration by the immune system. Nat Rev Immunol. (2017) 17:165–78. doi: 10.1038/nri.2016.150, PMID: 28163303 PMC5452982

[87] Zhang X Li H He M Wang J Wu Y Li Y. Immune system and sarcopenia: presented relationship and future perspective. Exp Gerontol. (2022) 164:111823. doi: 10.1016/j.exger.2022.111823, PMID: 35504482

[88] Wang Y Wehling-Henricks M Welc SS Fisher AL Zuo Q Tidball JG. Aging of the immune system causes reductions in muscle stem cell populations, promotes their shift to a fibrogenic phenotype, and modulates sarcopenia. FASEB J. (2018) 33:1415–27. doi: 10.1096/fj.201800973R 30130434 PMC6355087

