# Supplementary figures and images for: Bioinformatics and system biology approach to identify potential common pathogenesis for COVID-19 infection and sarcopenia
Source: Front Med (Lausanne). 2024 Jun 24;11:1378846. doi: 10.3389/fmed.2024.1378846 (PMC11228343; doi:10.3389/fmed.2024.1378846)

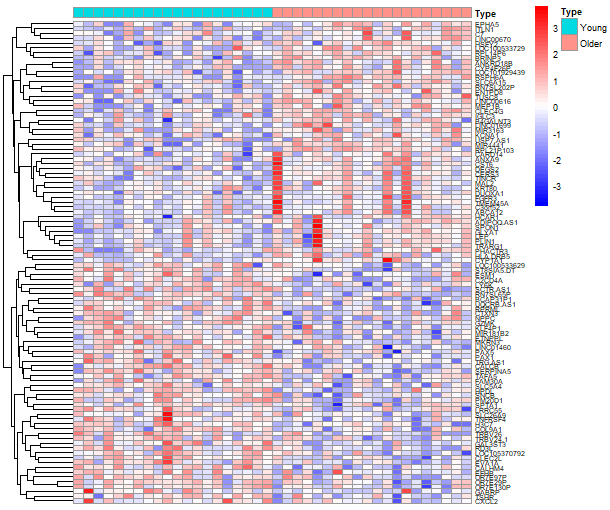

Supplement: Supplementary file 2 [file Data_Sheet_2.ZIP › raw data3/06.diff/GSE111016/GSE111016heatmap.png]

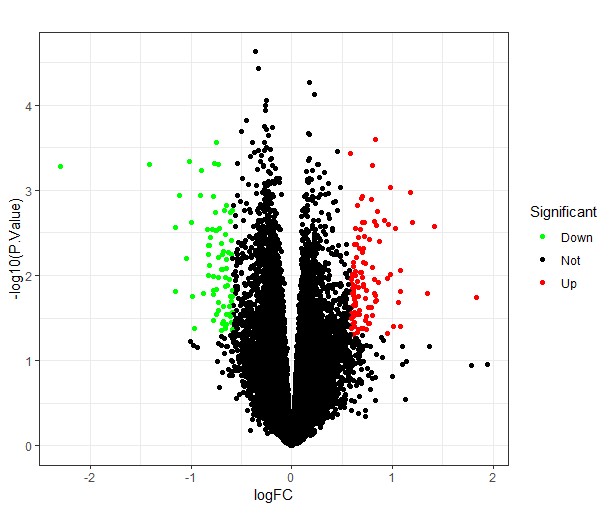

Supplement: Supplementary file 2 [file Data_Sheet_2.ZIP › raw data3/06.diff/GSE111016/GSE111016vol.png]

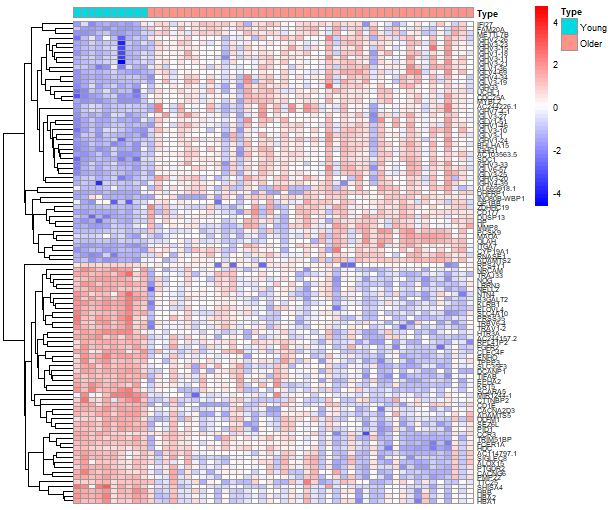

Supplement: Supplementary file 2 [file Data_Sheet_2.ZIP › raw data3/06.diff/GSE111016/GSE171110heatmap.png]

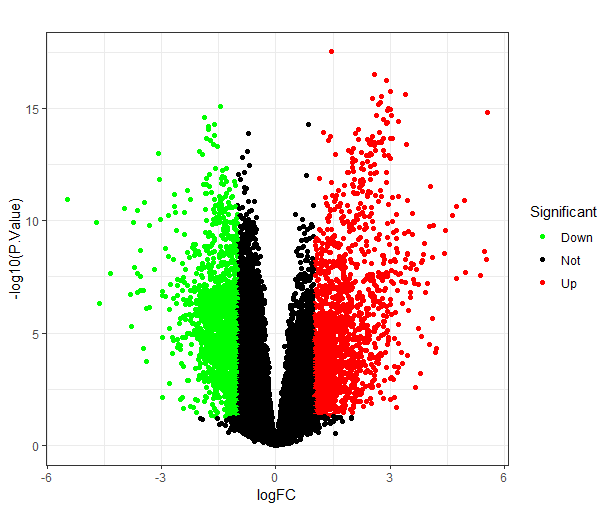

Supplement: Supplementary file 2 [file Data_Sheet_2.ZIP › raw data3/06.diff/GSE111016/GSE171110vol.png]

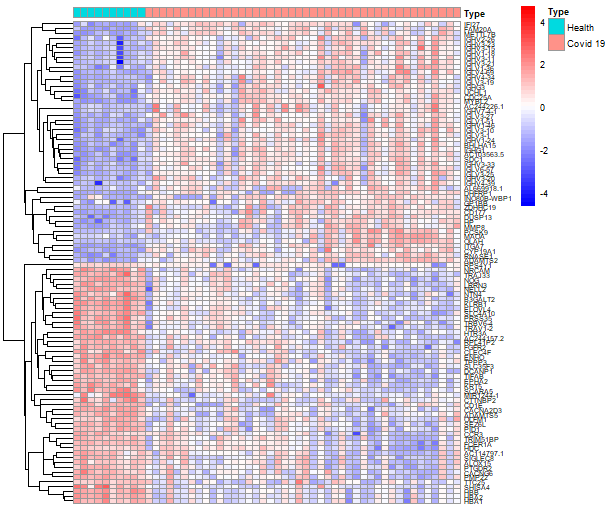

Supplement: Supplementary file 2 [file Data_Sheet_2.ZIP › raw data3/06.diff/GSE171110/GSE171110heatmap.png]

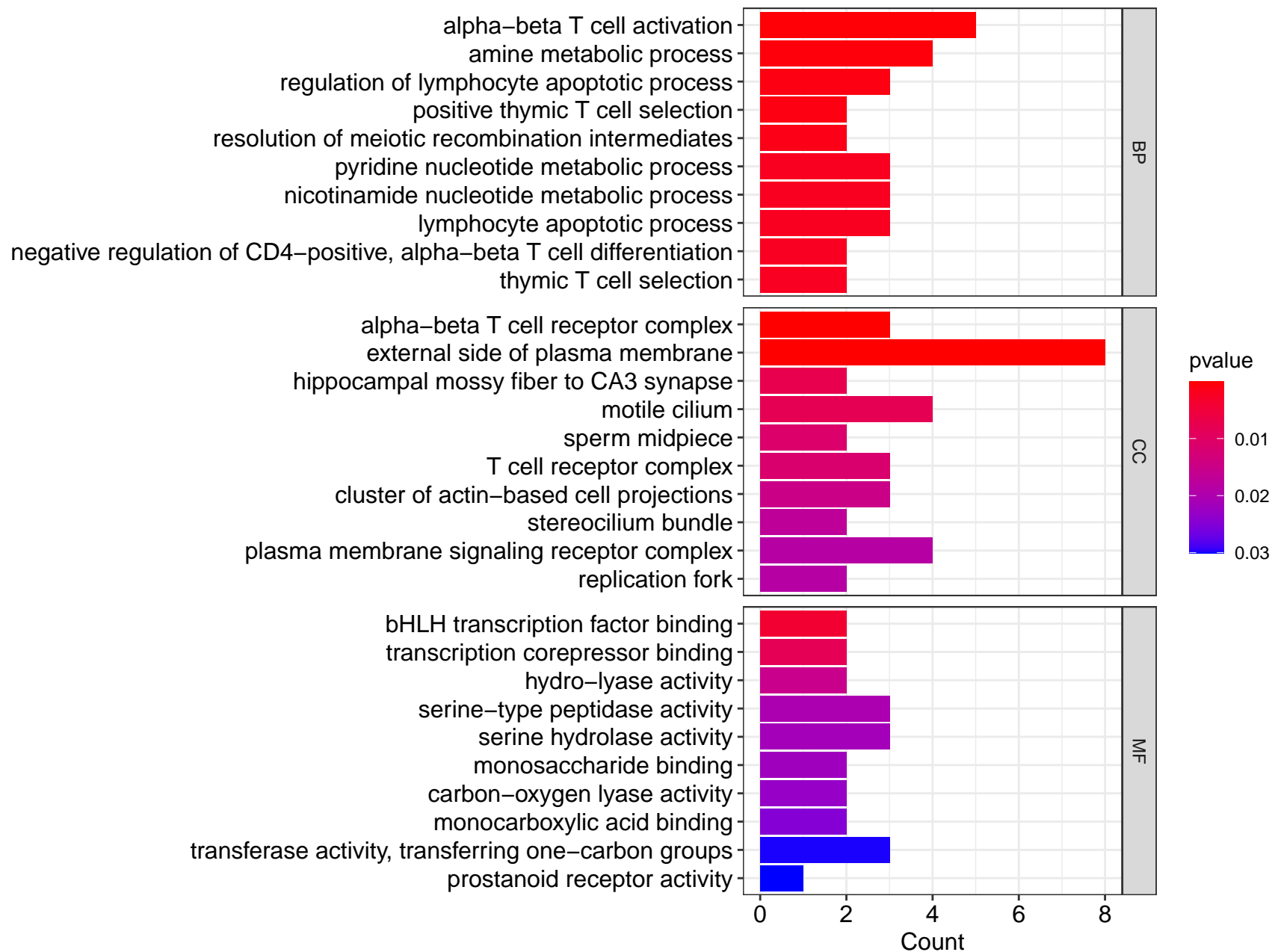

Supplement: Supplementary file 2 [file Data_Sheet_2.ZIP › raw data3/07.GO/barplot.pdf]

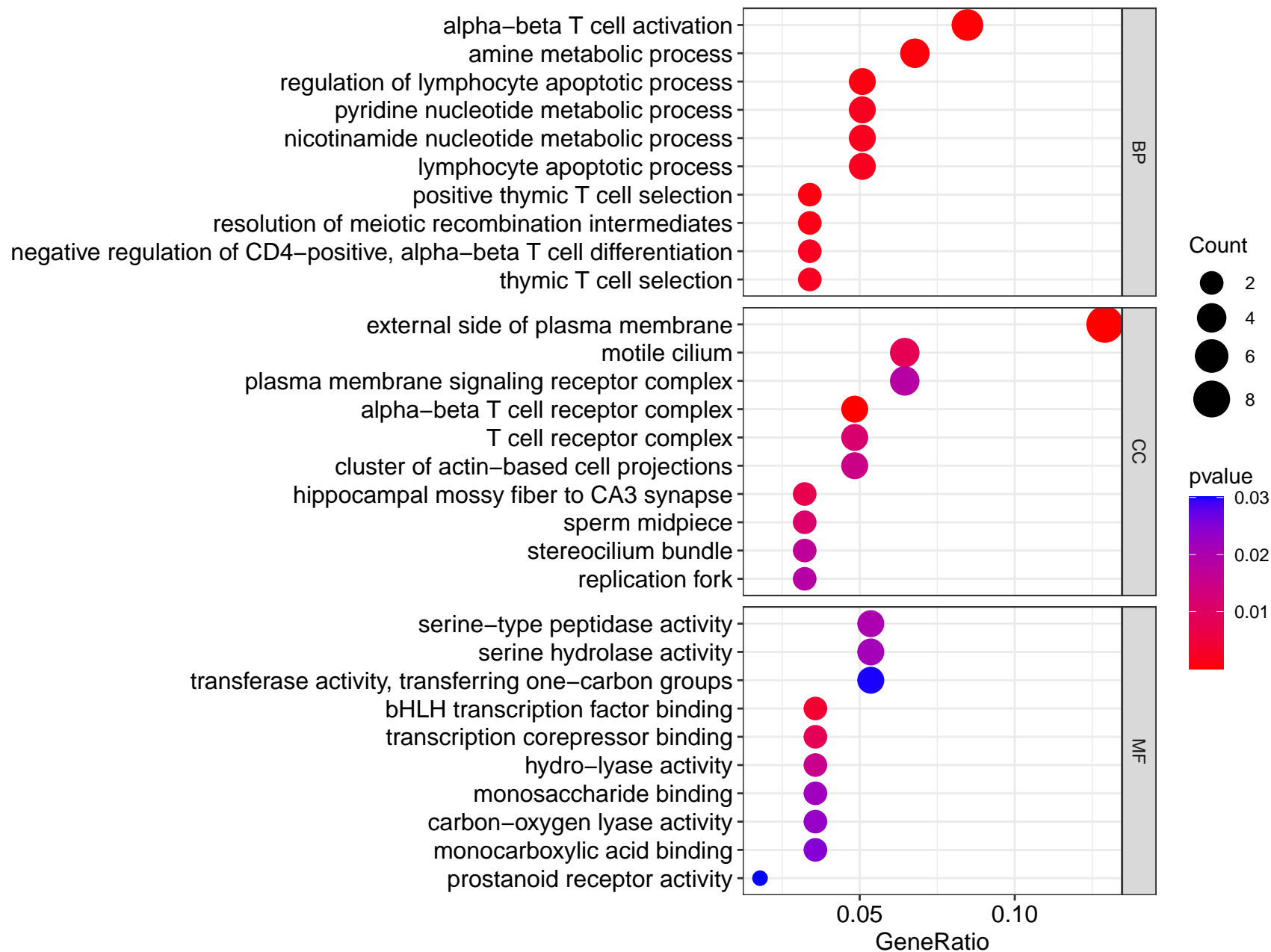

Supplement: Supplementary file 2 [file Data_Sheet_2.ZIP › raw data3/07.GO/bubble.pdf]

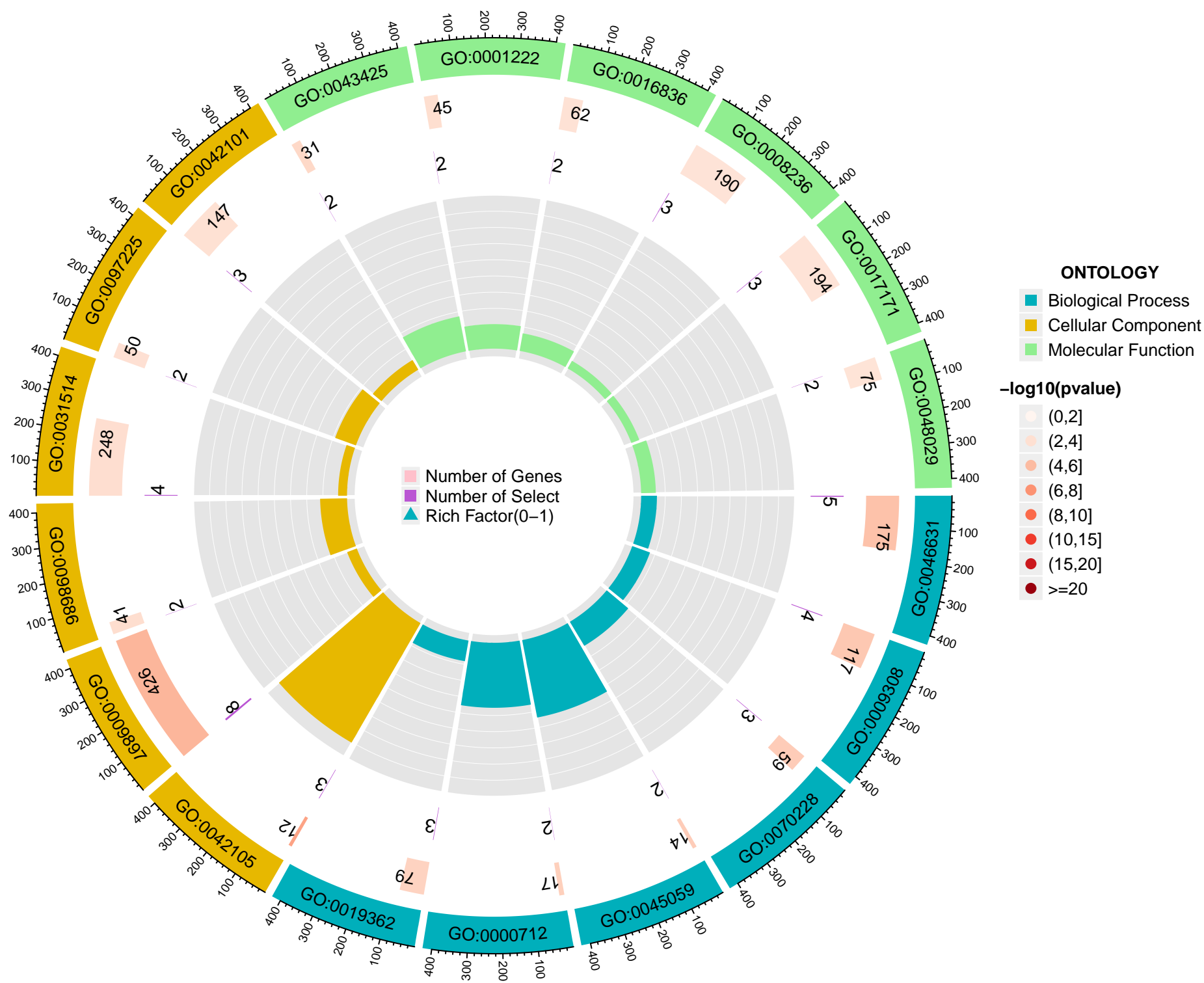

Supplement: Supplementary file 2 [file Data_Sheet_2.ZIP › raw data3/07.GO/GO.circlize.pdf]

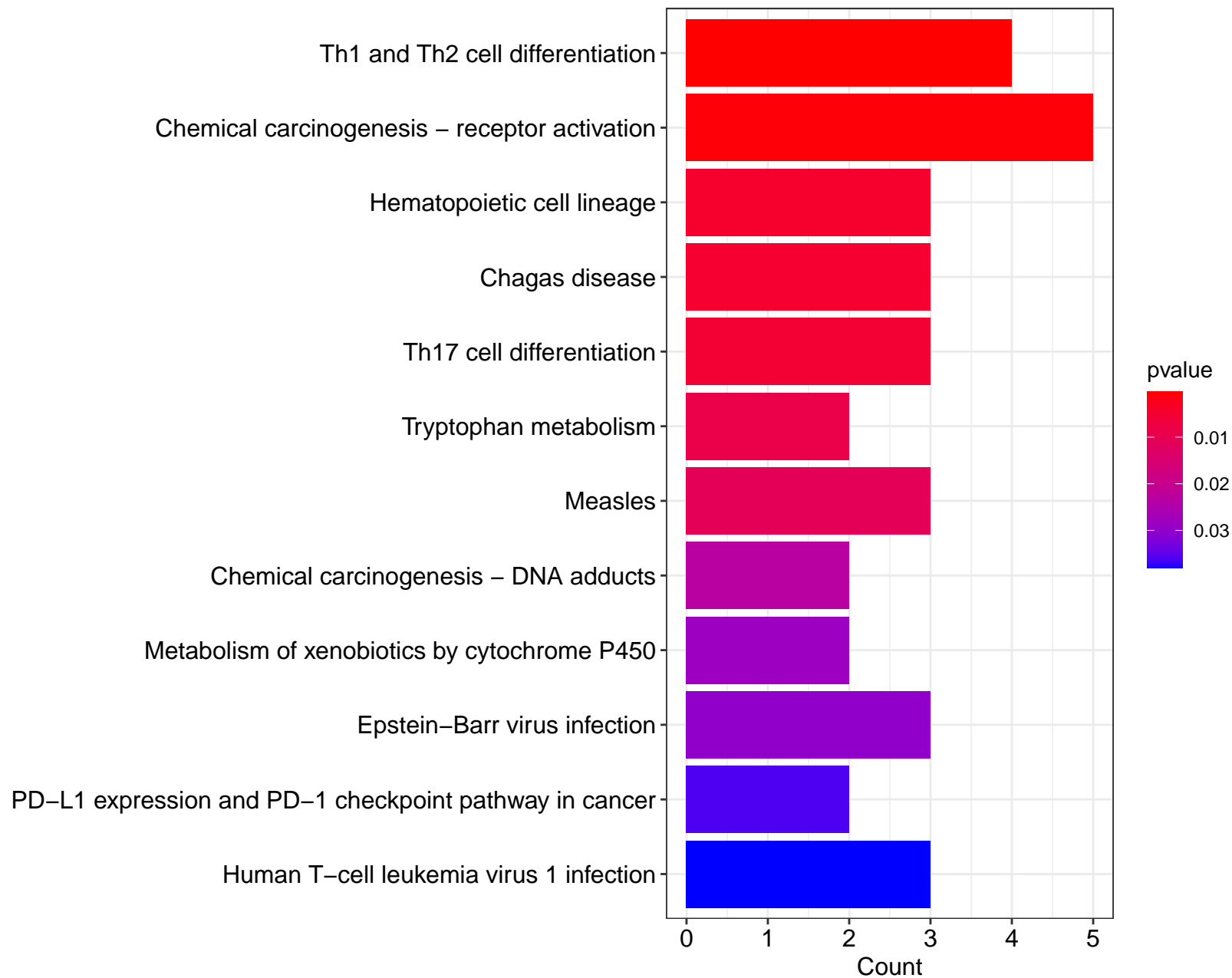

Supplement: Supplementary file 2 [file Data_Sheet_2.ZIP › raw data3/08.KEGG/barplot.pdf]

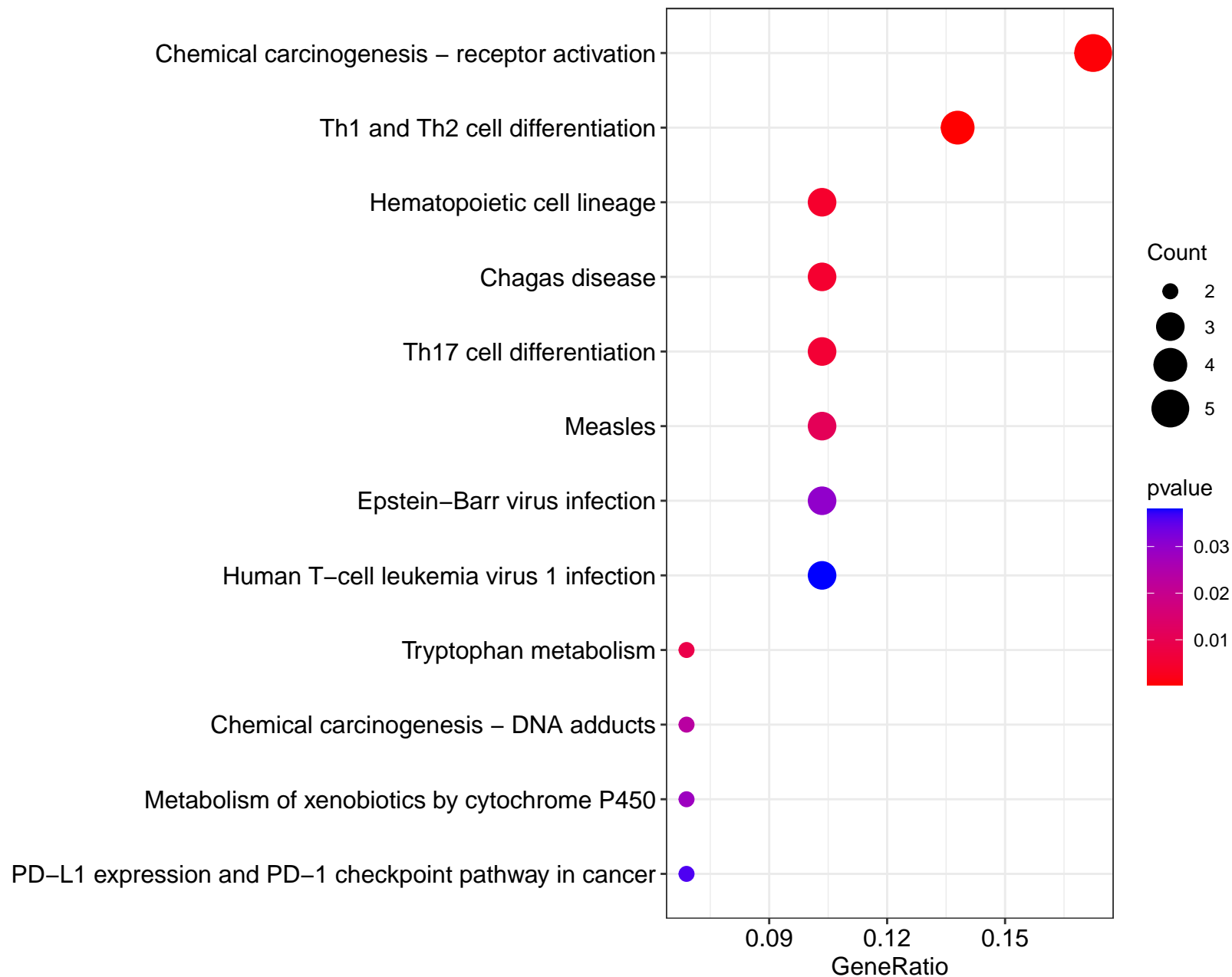

Supplement: Supplementary file 2 [file Data_Sheet_2.ZIP › raw data3/08.KEGG/bubble.pdf]

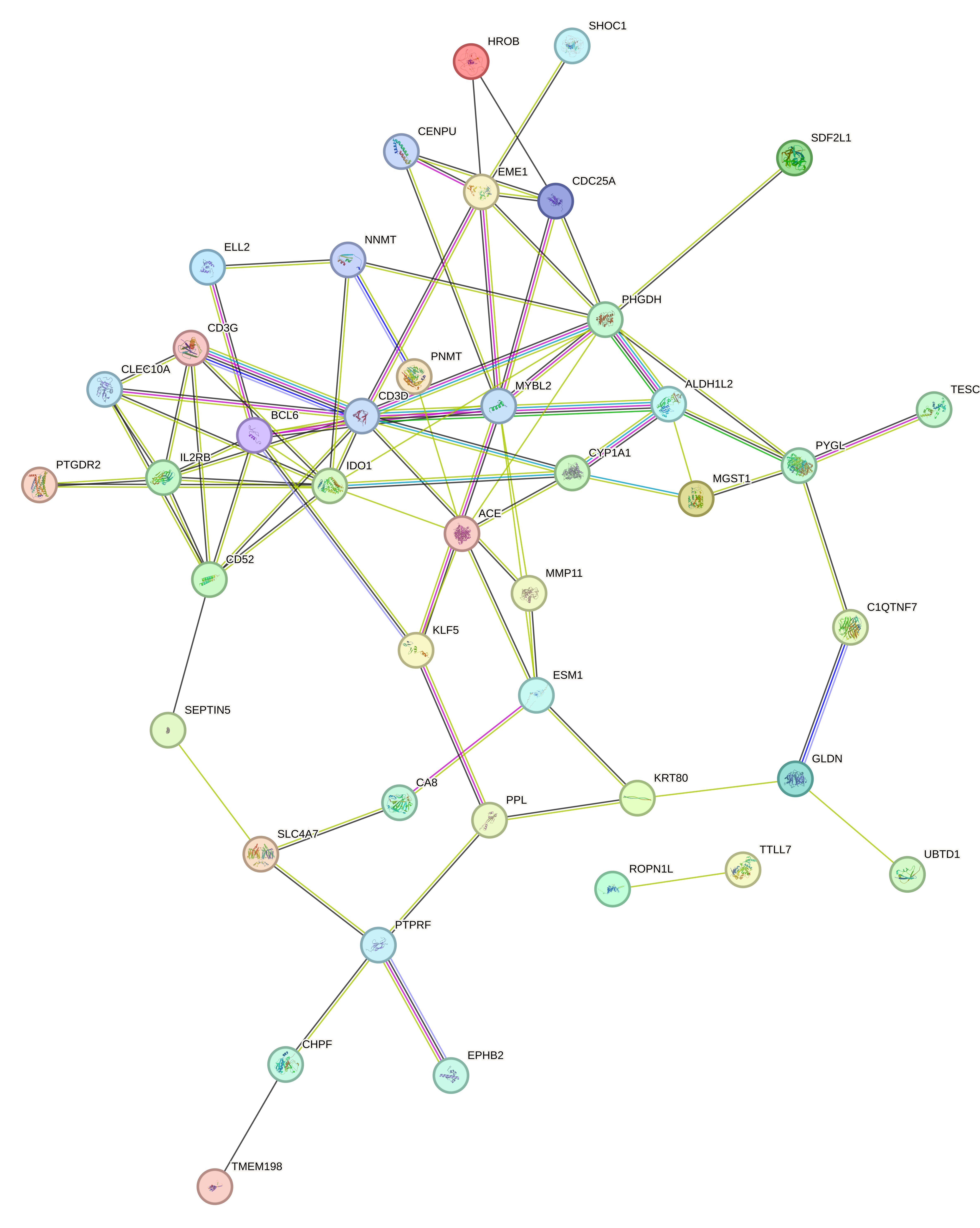

Supplement: Supplementary file 3 [file Data_Sheet_3.ZIP › raw data4/09.PPI/string_hires_image.png]

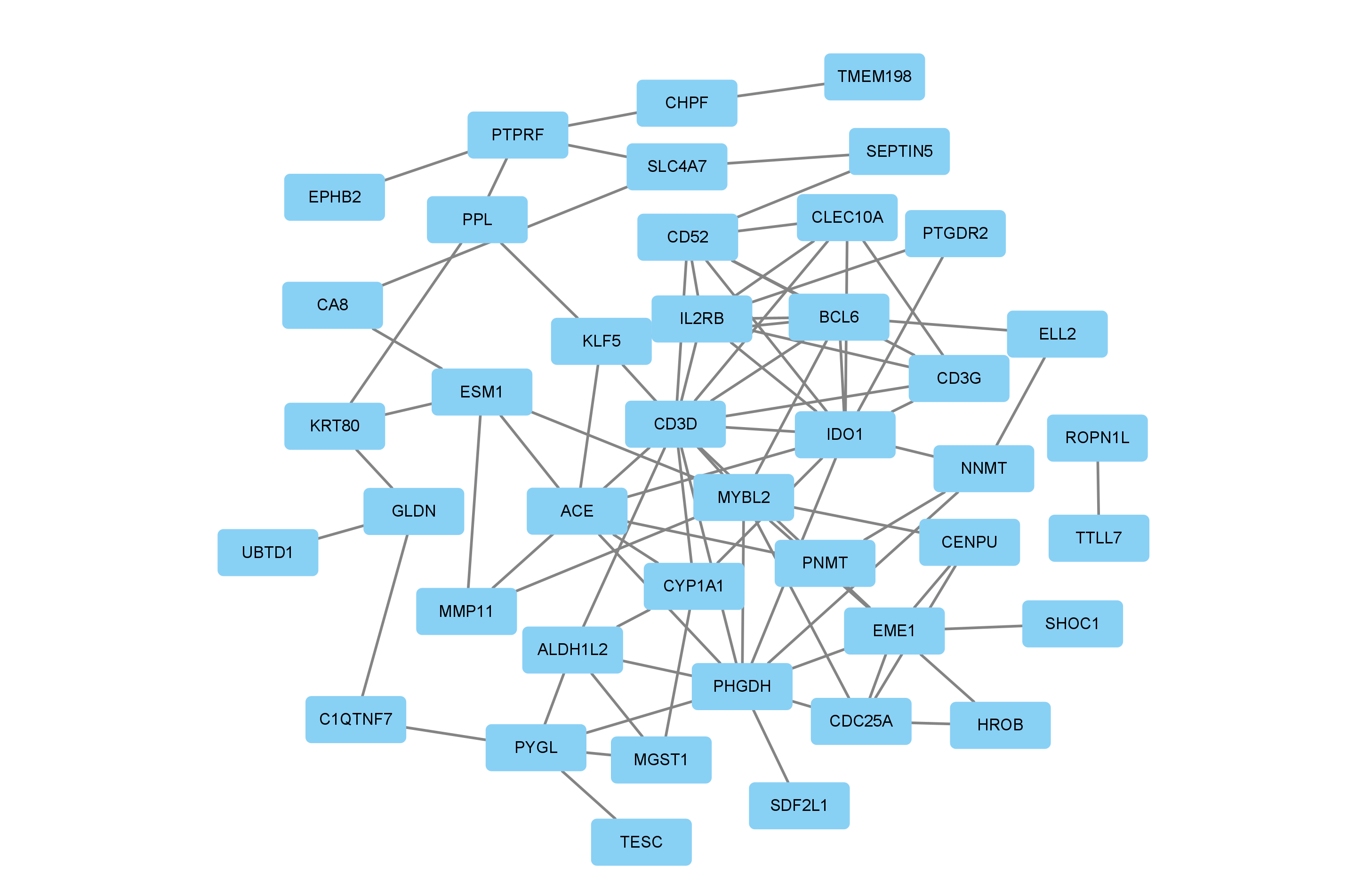

Supplement: Supplementary file 3 [file Data_Sheet_3.ZIP › raw data4/10.precyto/string_interactions_short.txt.png]

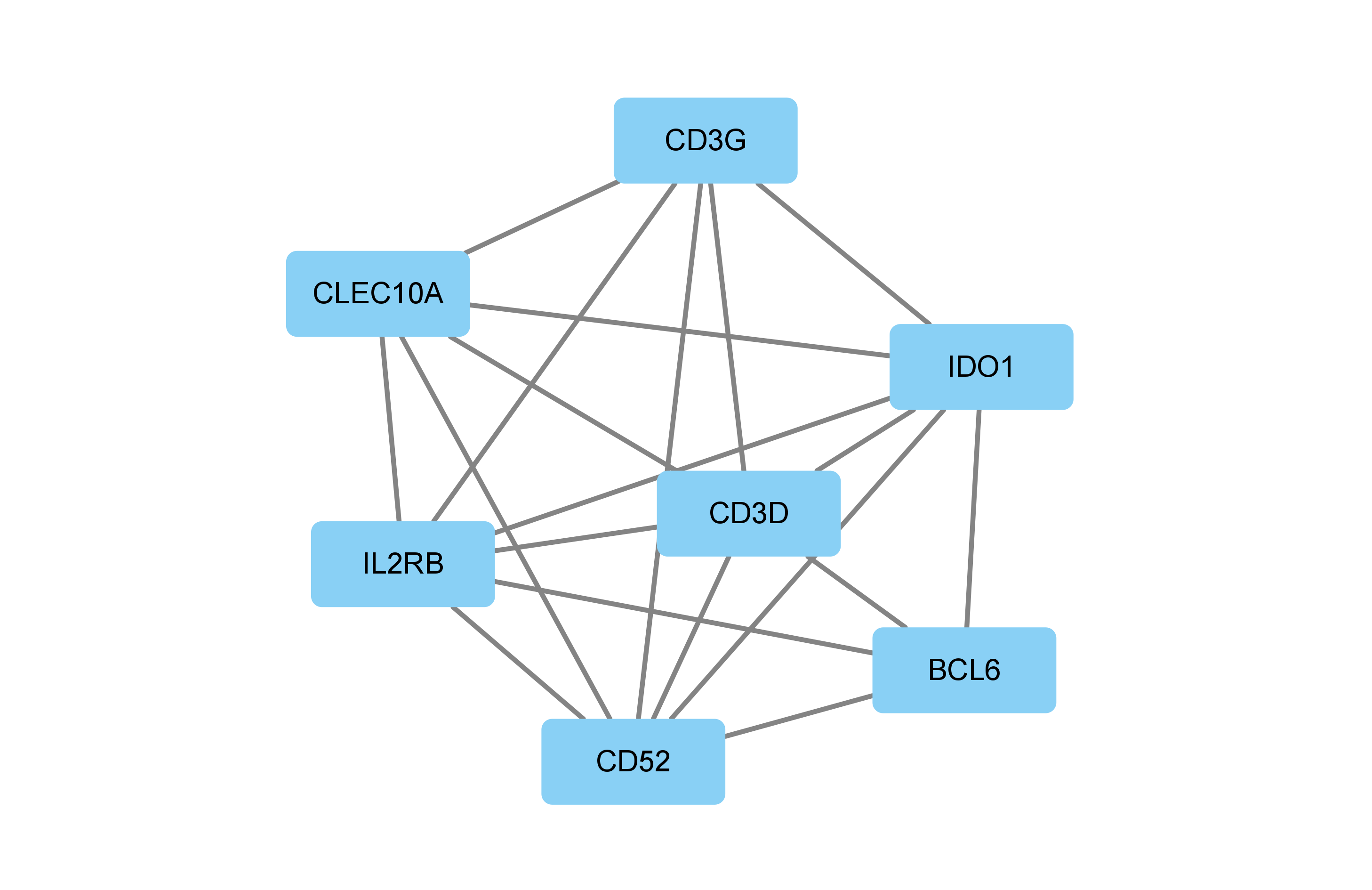

Supplement: Supplementary file 3 [file Data_Sheet_3.ZIP › raw data4/11.MCODE/1.png]

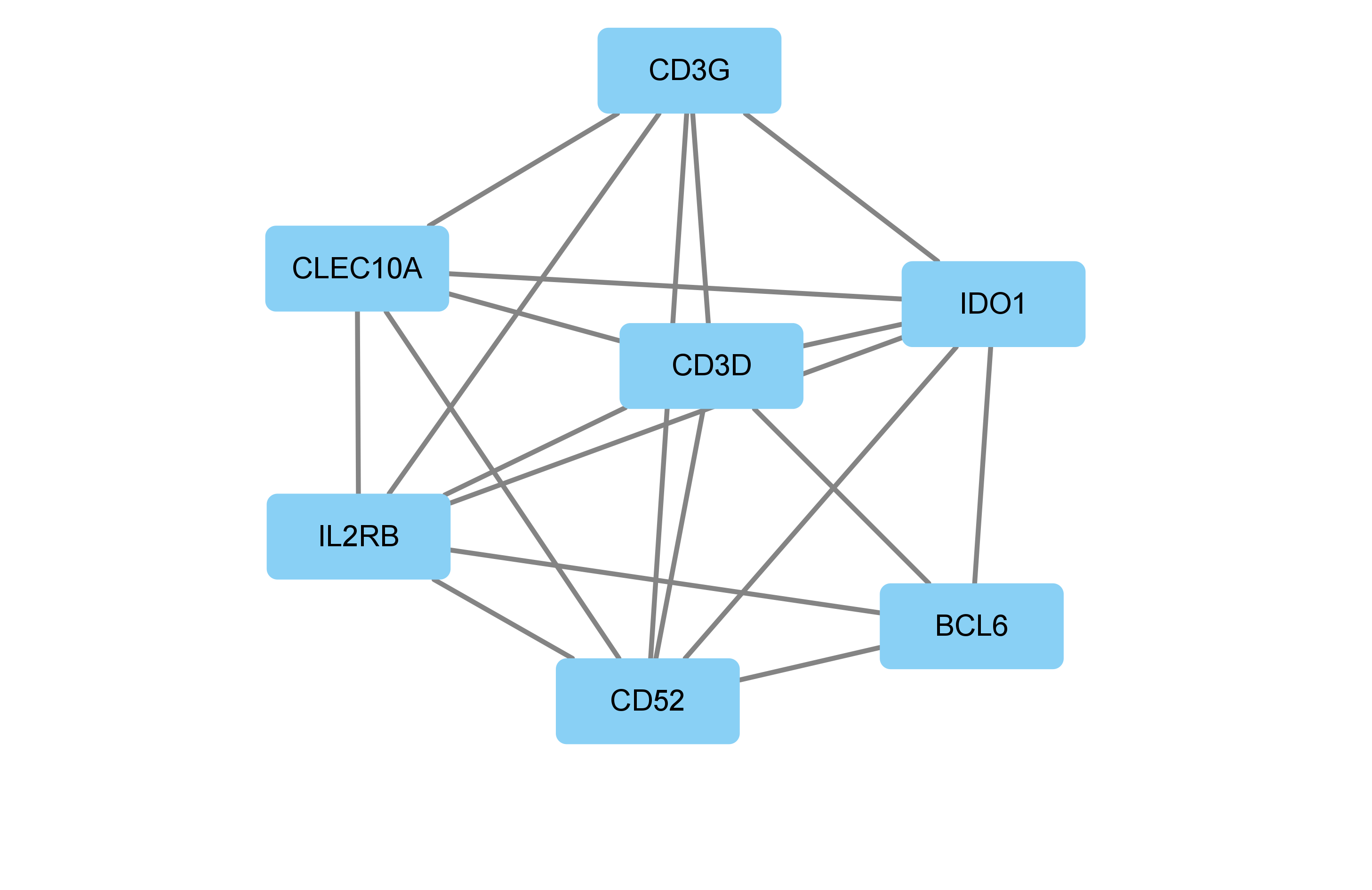

Supplement: Supplementary file 3 [file Data_Sheet_3.ZIP › raw data4/11.MCODE/11.png]

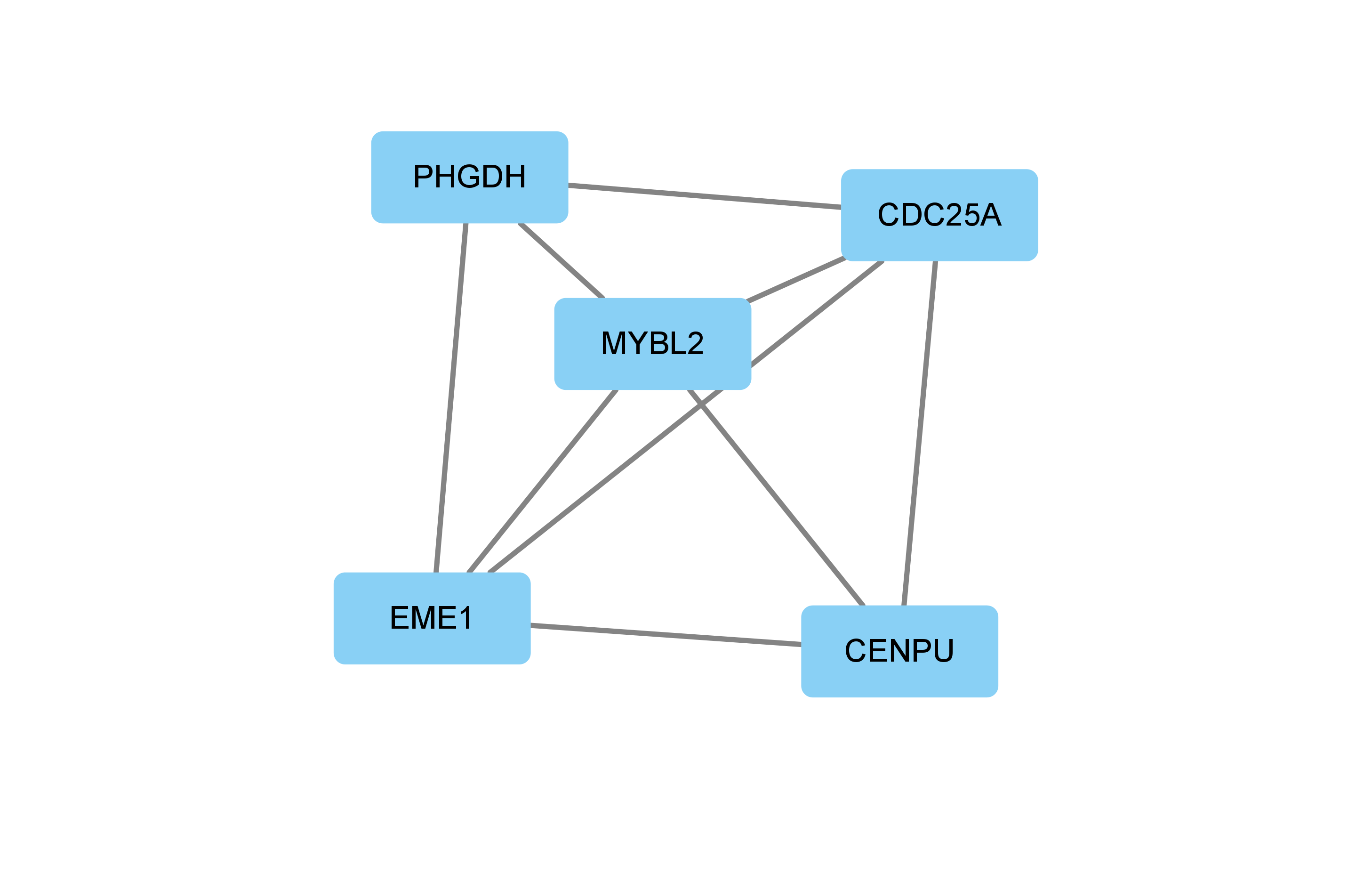

Supplement: Supplementary file 3 [file Data_Sheet_3.ZIP › raw data4/11.MCODE/2.png]

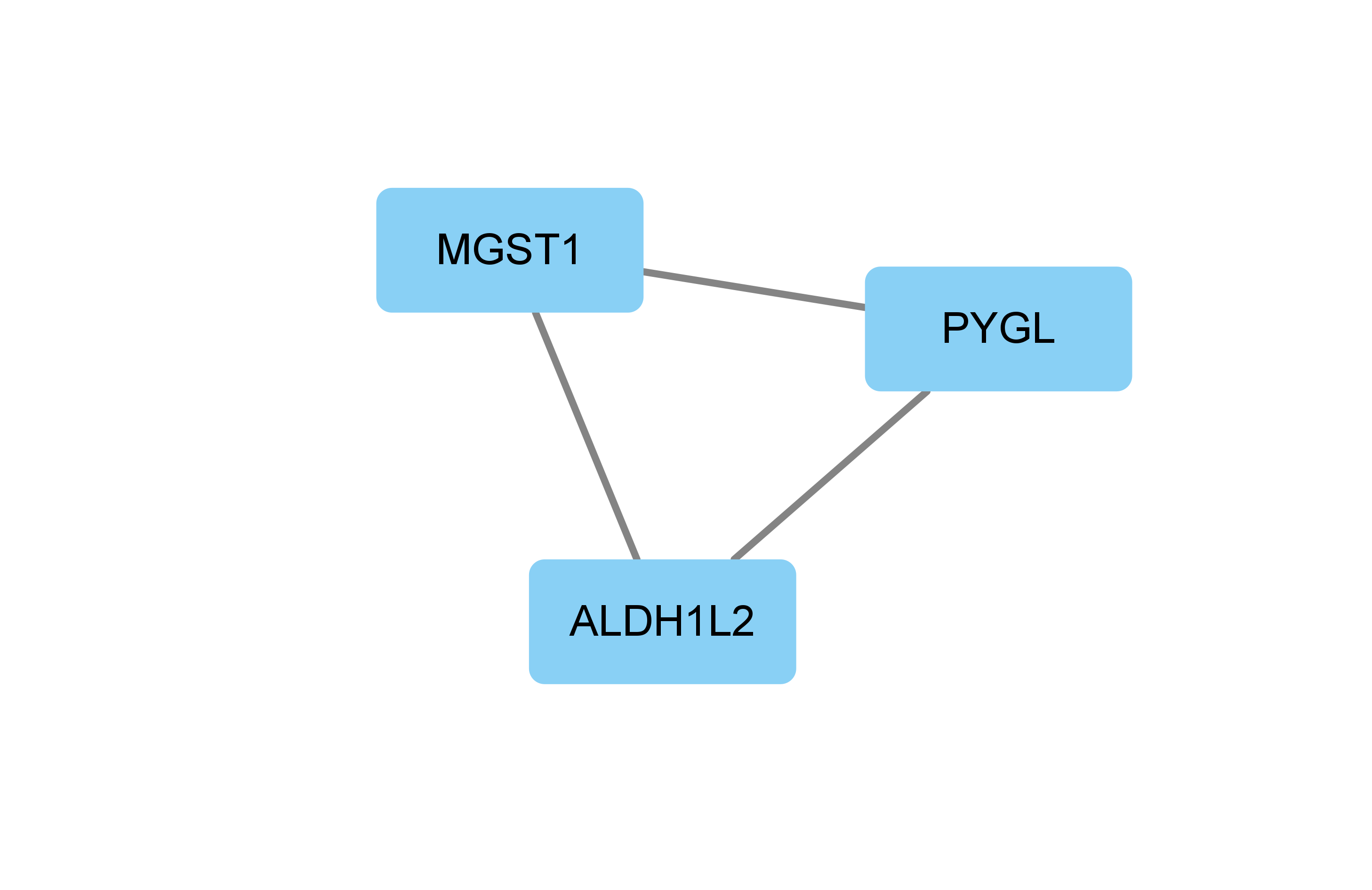

Supplement: Supplementary file 3 [file Data_Sheet_3.ZIP › raw data4/11.MCODE/3.png]

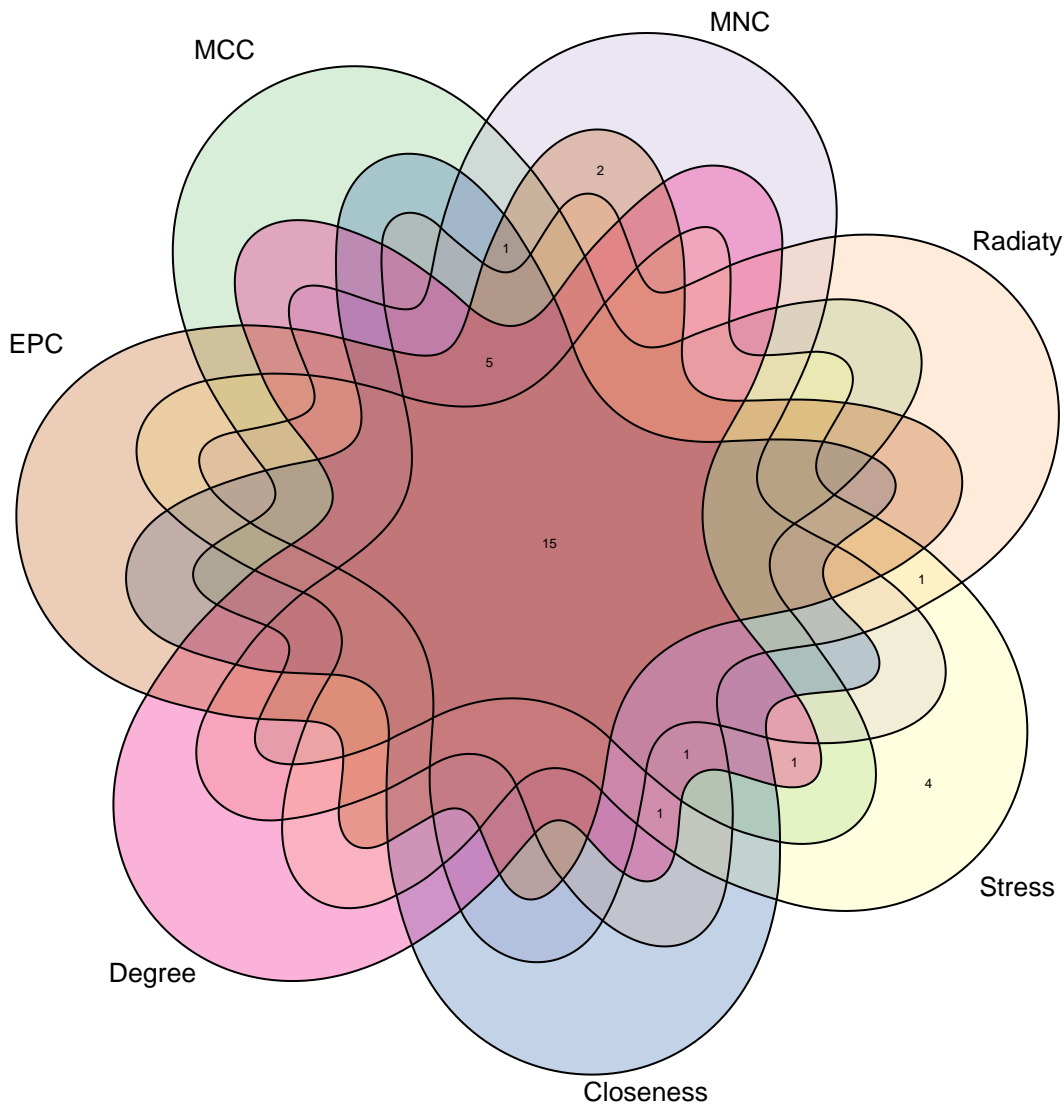

Supplement: Supplementary file 3 [file Data_Sheet_3.ZIP › raw data4/14.cytoHubba/Veen.pdf]

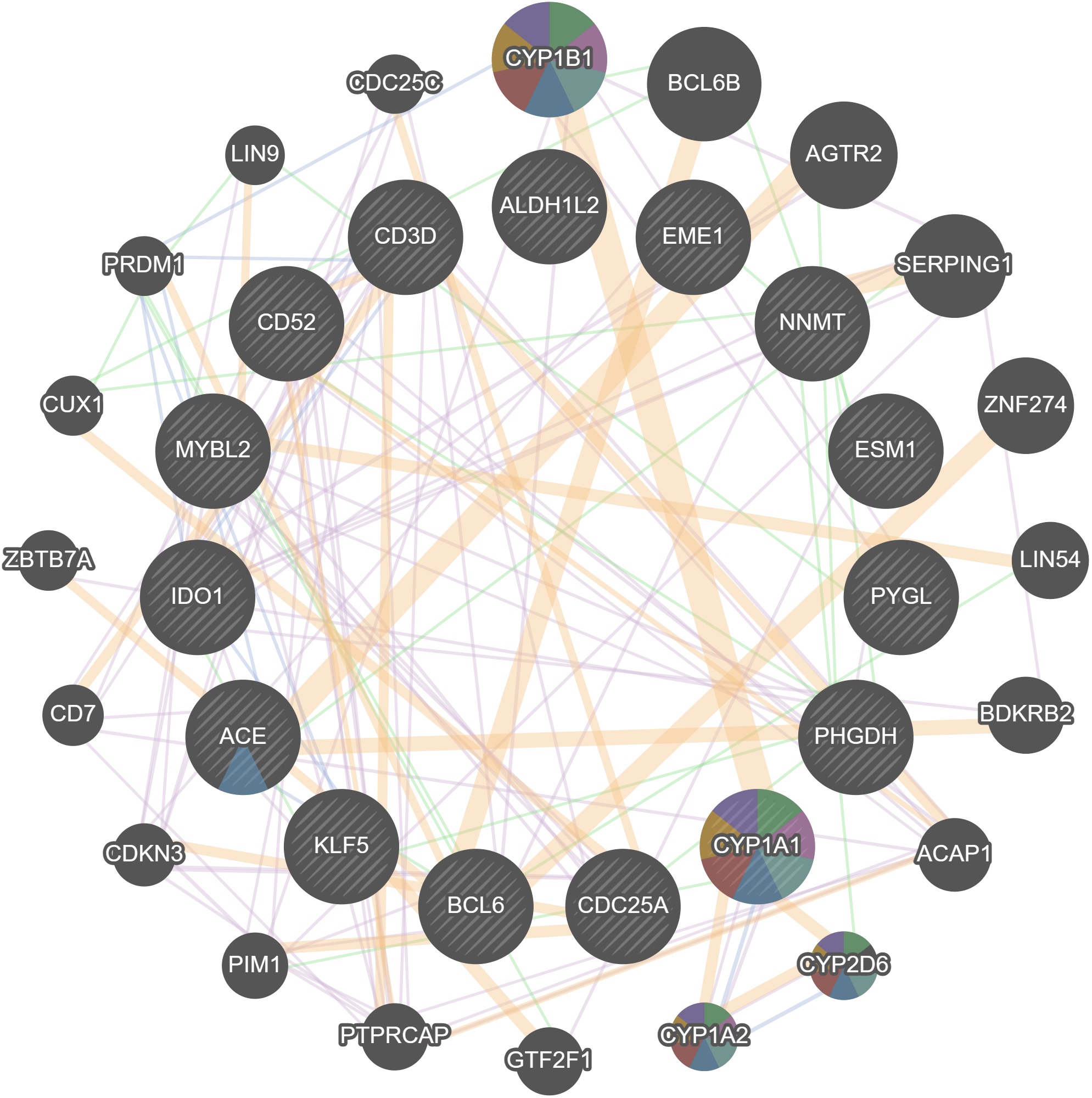

Supplement: Supplementary file 3 [file Data_Sheet_3.ZIP › raw data4/15.GeneMANIA/genemania-network.jpg]

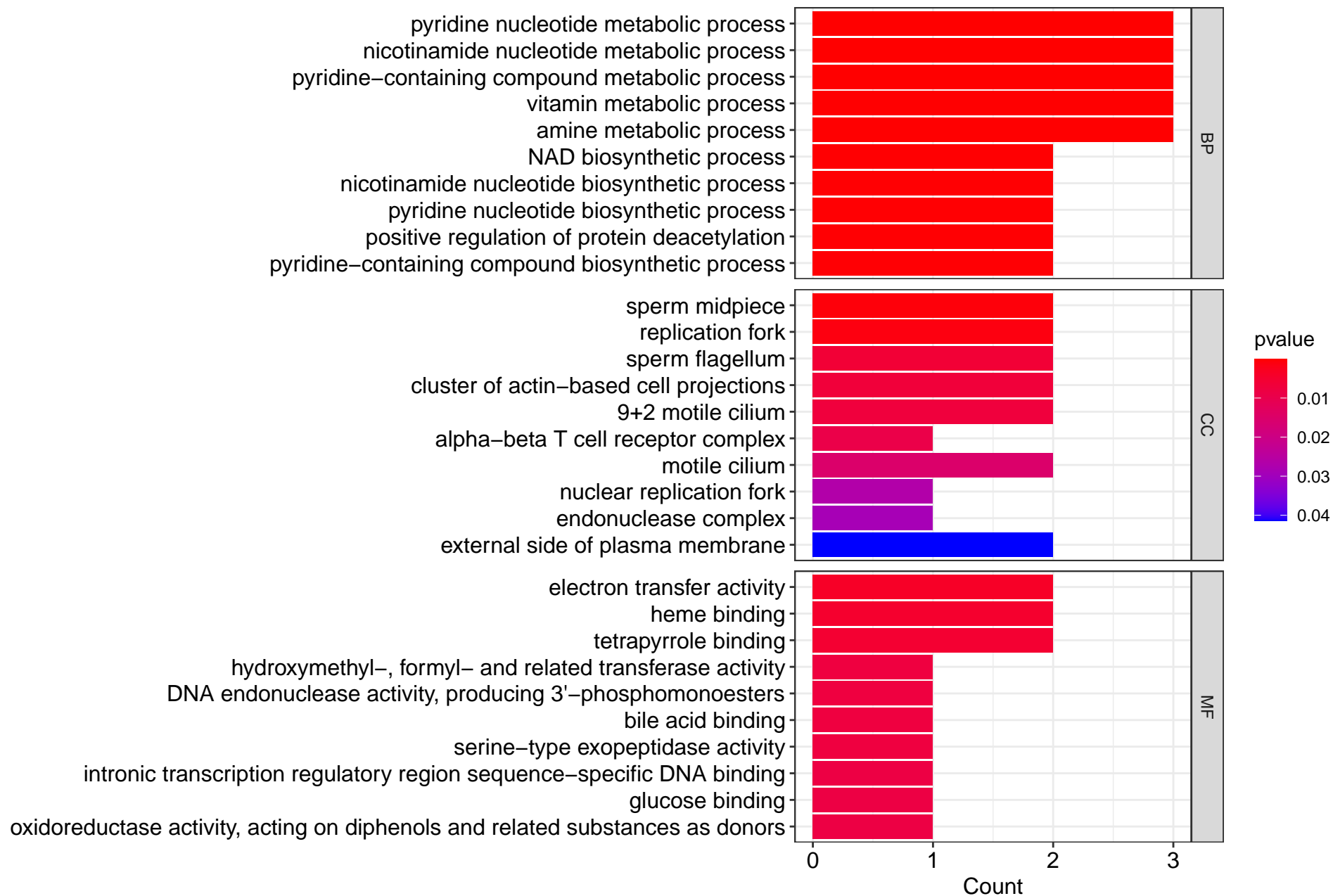

Supplement: Supplementary file 3 [file Data_Sheet_3.ZIP › raw data4/16.hubheneGO/barplot.pdf]

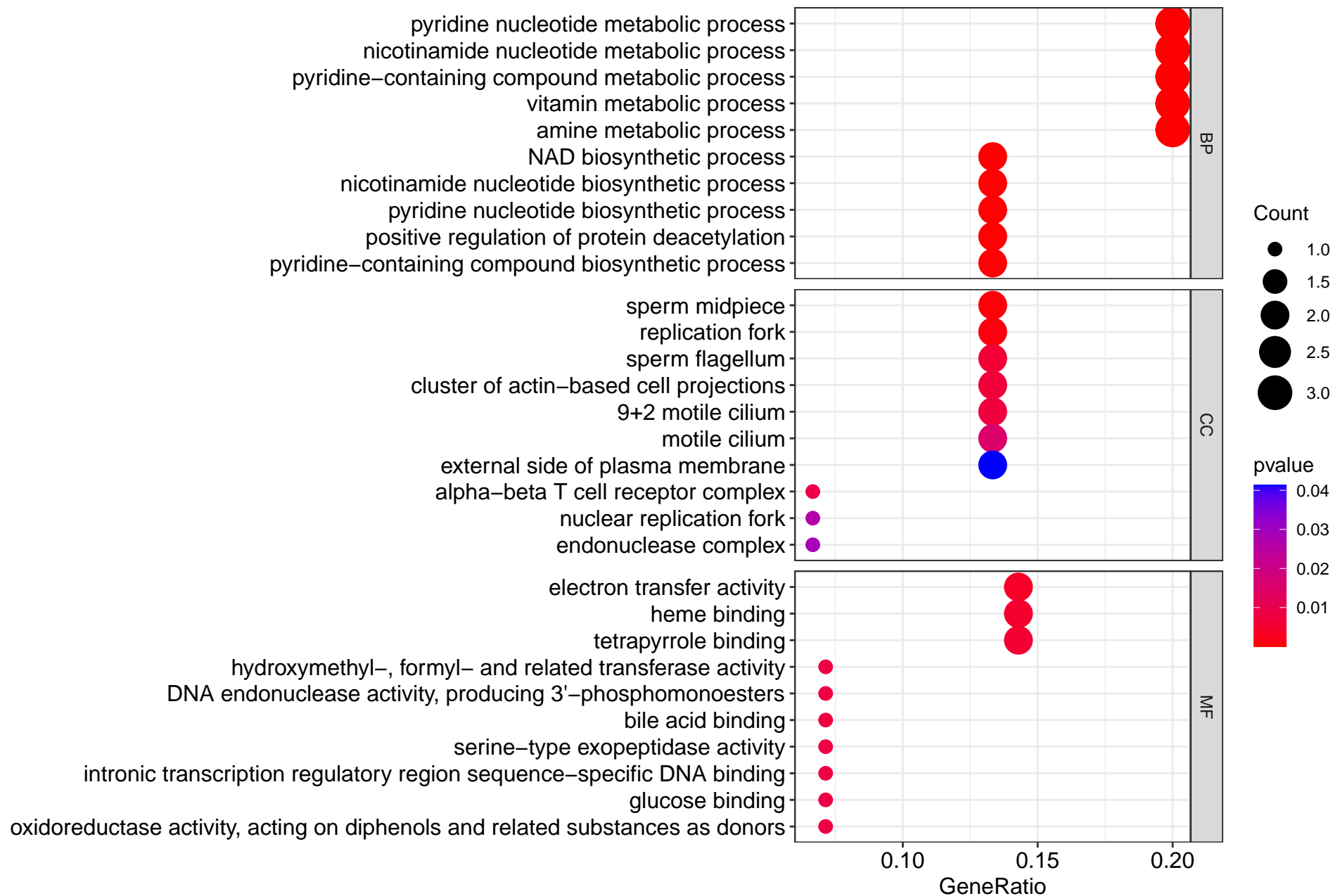

Supplement: Supplementary file 3 [file Data_Sheet_3.ZIP › raw data4/16.hubheneGO/bubble.pdf]

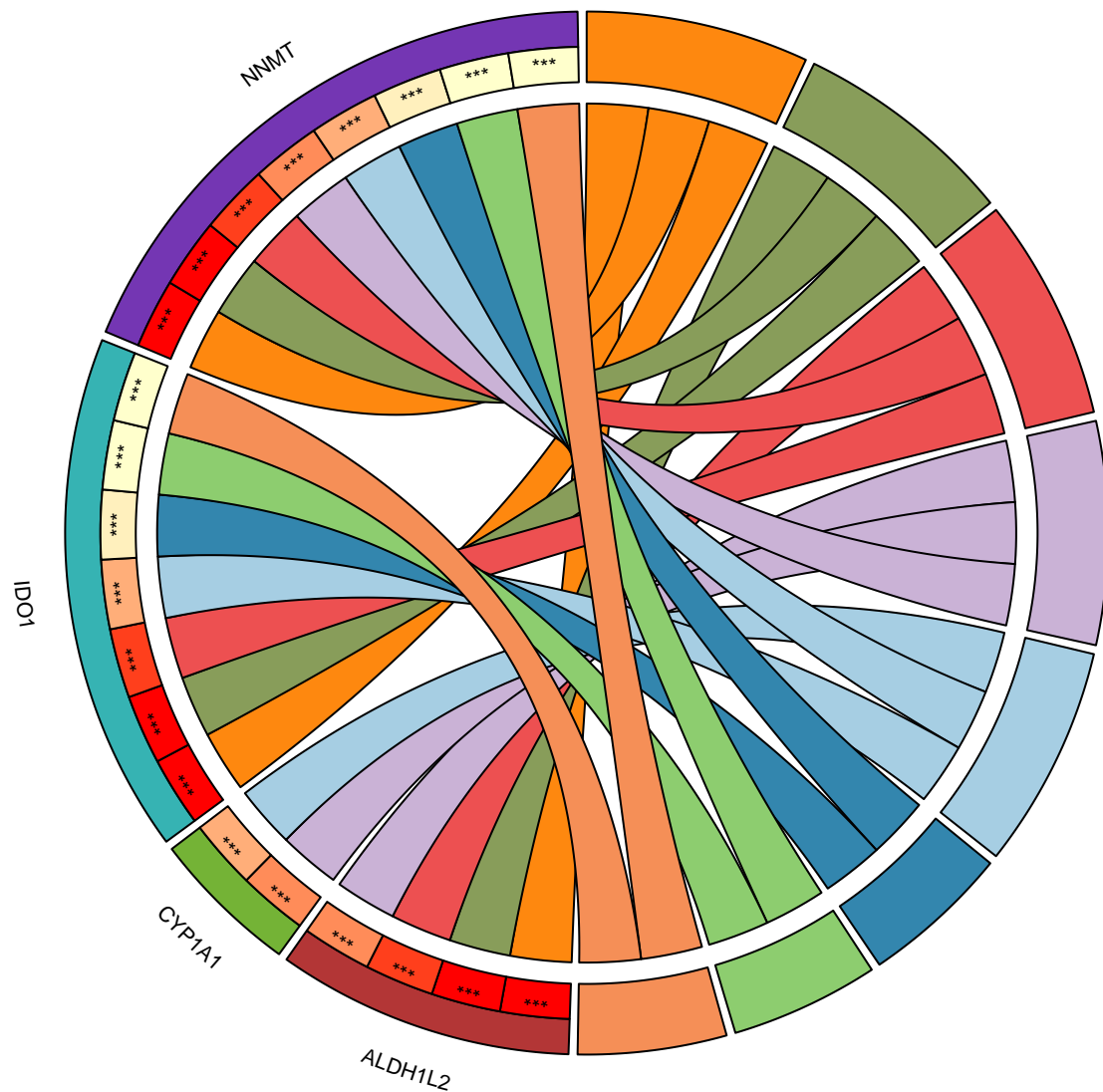

$-\log_{10}(\text{pvalue})$

3.65

4.06125

4.49

Supplement: Supplementary file 3 [file Data_Sheet_3.ZIP › raw data4/17.hubgeneGOcircos/GO.circos.pdf]

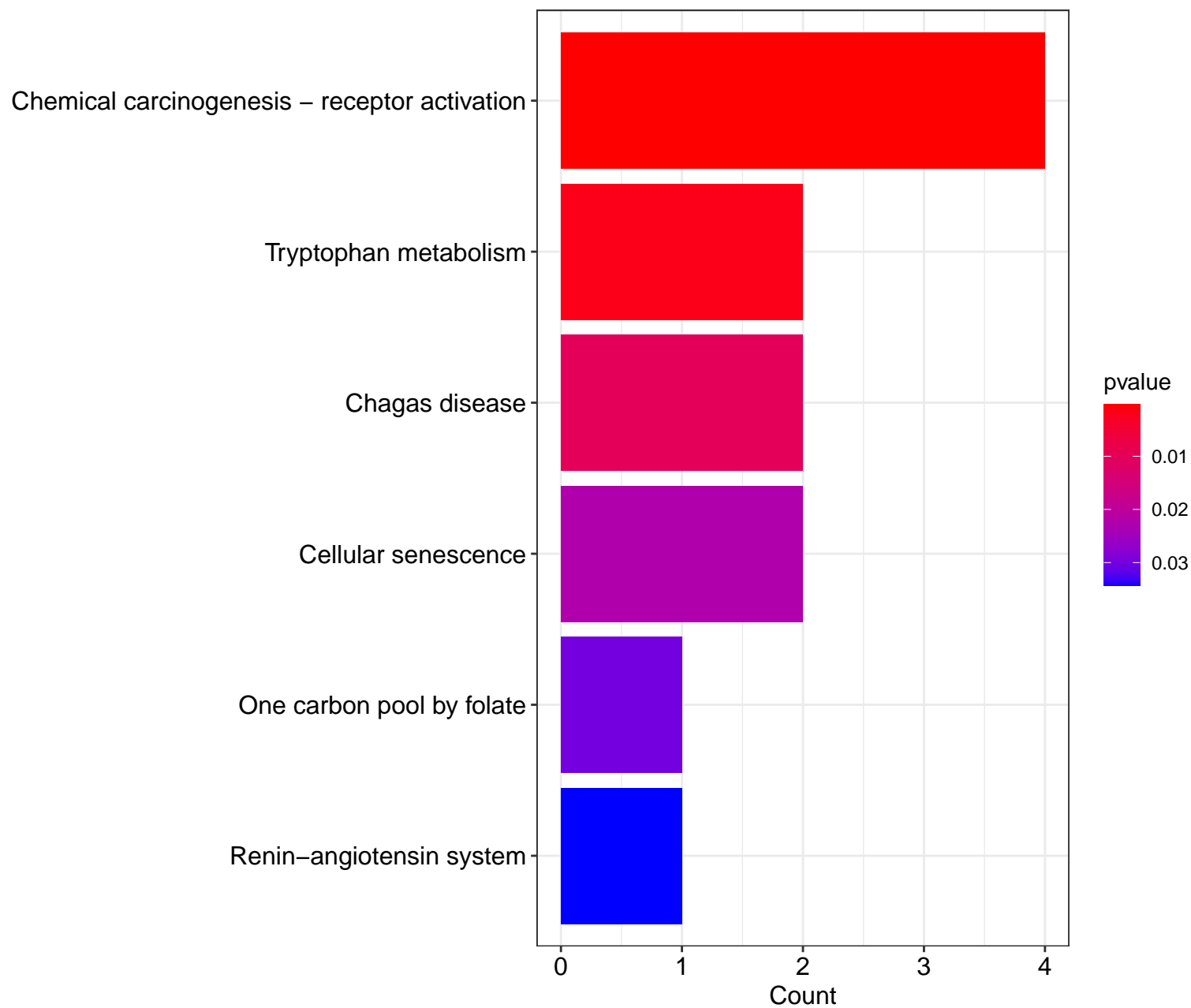

Supplement: Supplementary file 3 [file Data_Sheet_3.ZIP › raw data4/18.hubgeneKEGG/barplot.pdf]

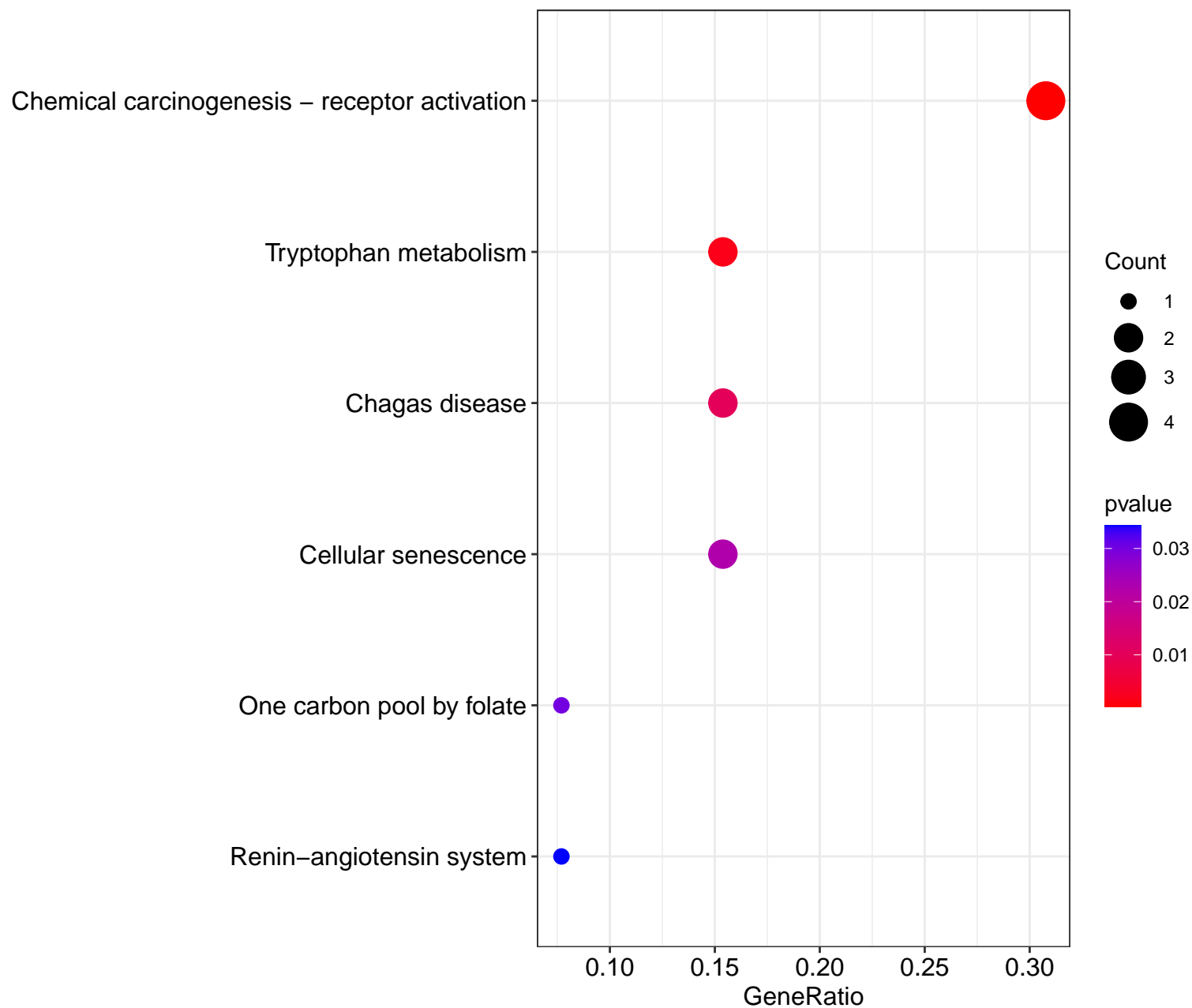

Supplement: Supplementary file 3 [file Data_Sheet_3.ZIP › raw data4/18.hubgeneKEGG/bubble.pdf]

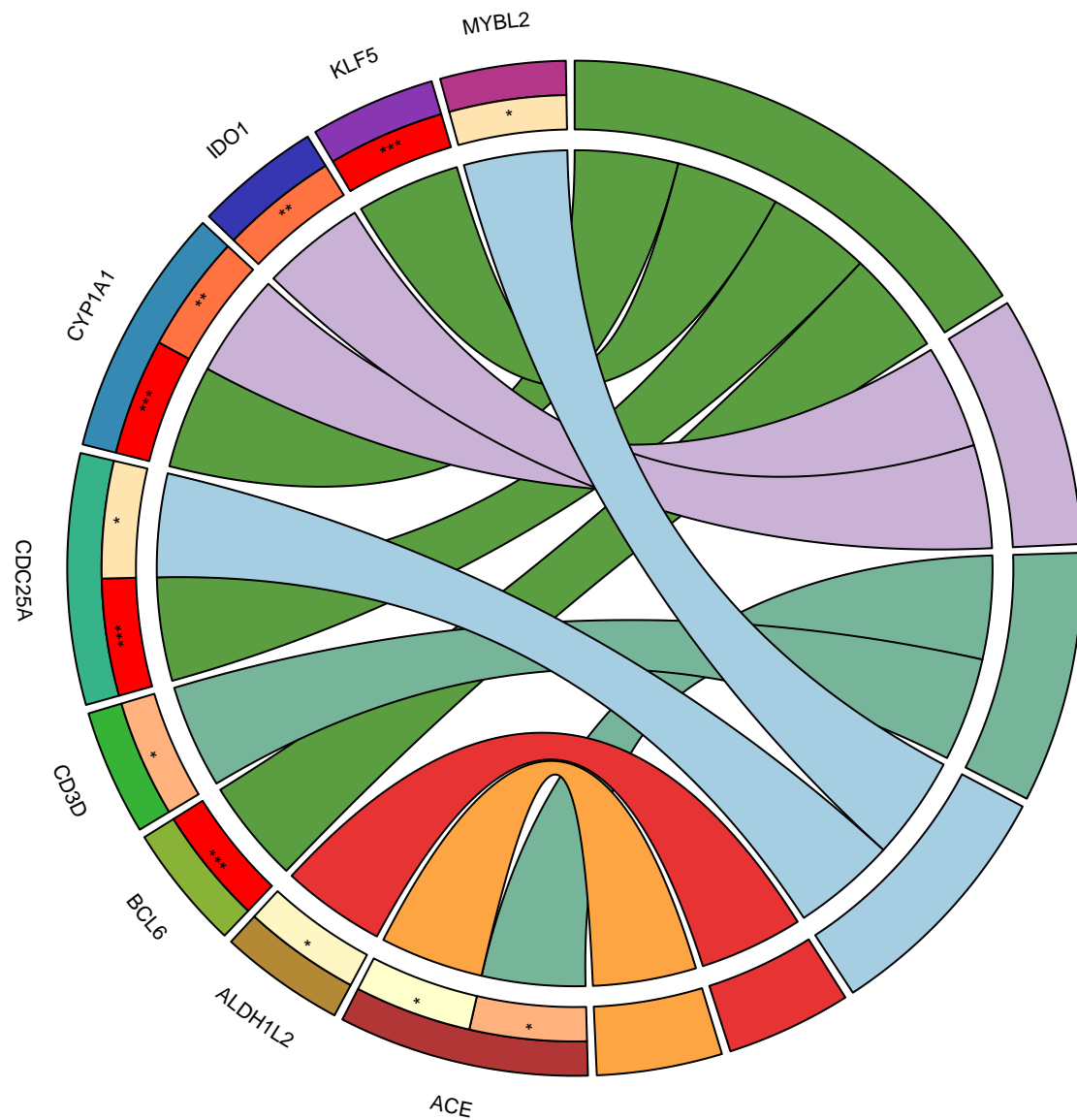

Supplement: Supplementary file 3 [file Data_Sheet_3.ZIP › raw data4/19.hubgeneKEGGcircos/KEGG.circos.pdf]

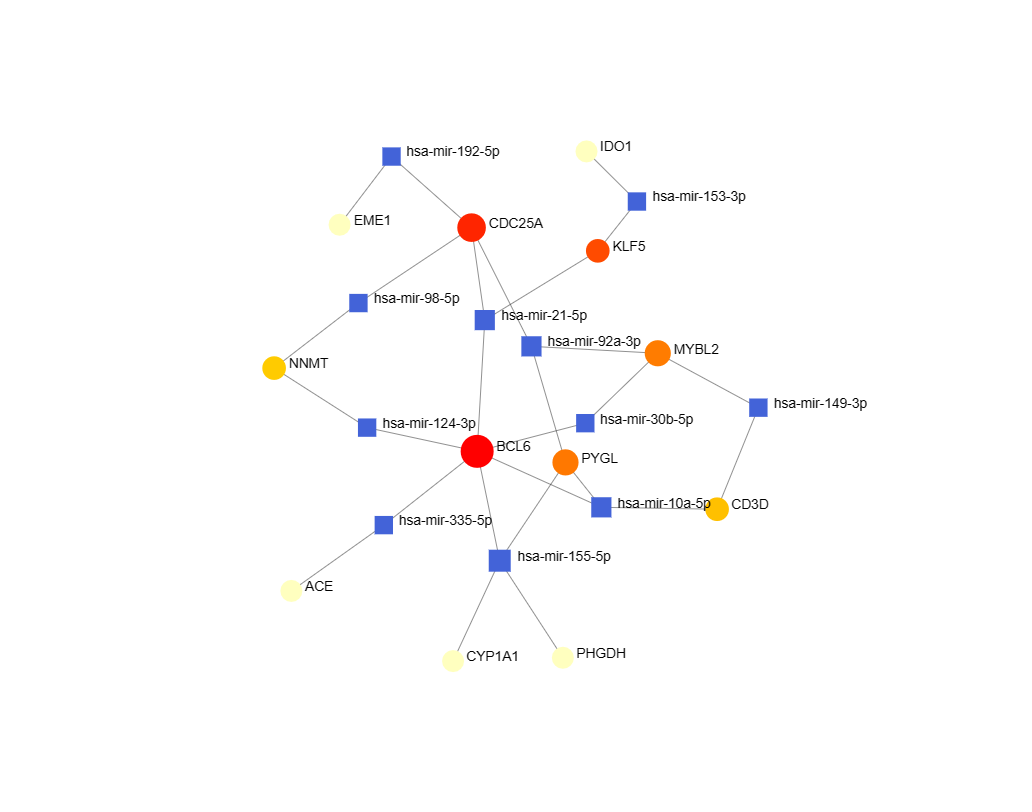

Supplement: Supplementary file 3 [file Data_Sheet_3.ZIP › raw data4/20.NetworkanalystTfMirna/2023-9-12 at 16.47.38.png]

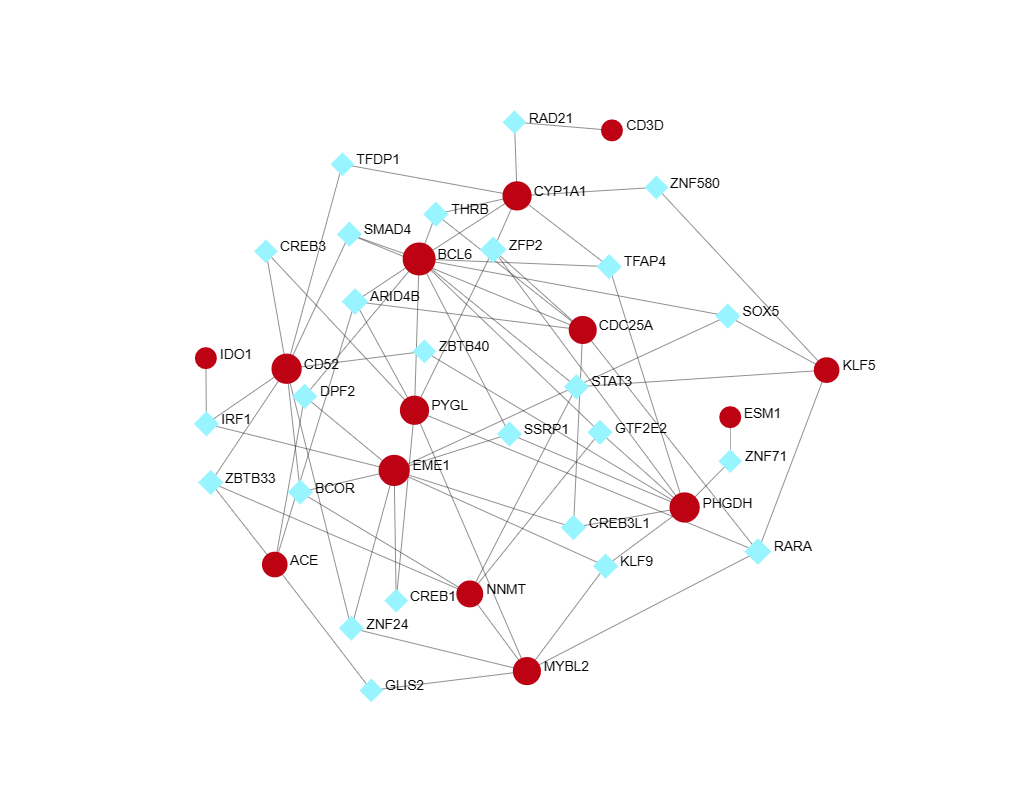

Supplement: Supplementary file 3 [file Data_Sheet_3.ZIP › raw data4/20.NetworkanalystTfMirna/2023-9-12 at 16.51.37.png]

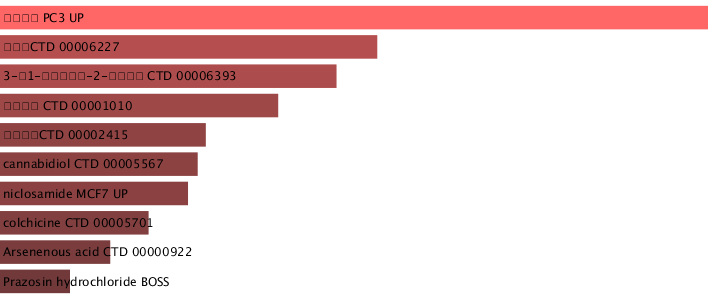

Supplement: Supplementary file 3 [file Data_Sheet_3.ZIP › raw data4/21.DSigDB/DSigDB_bar_graph (1).png]

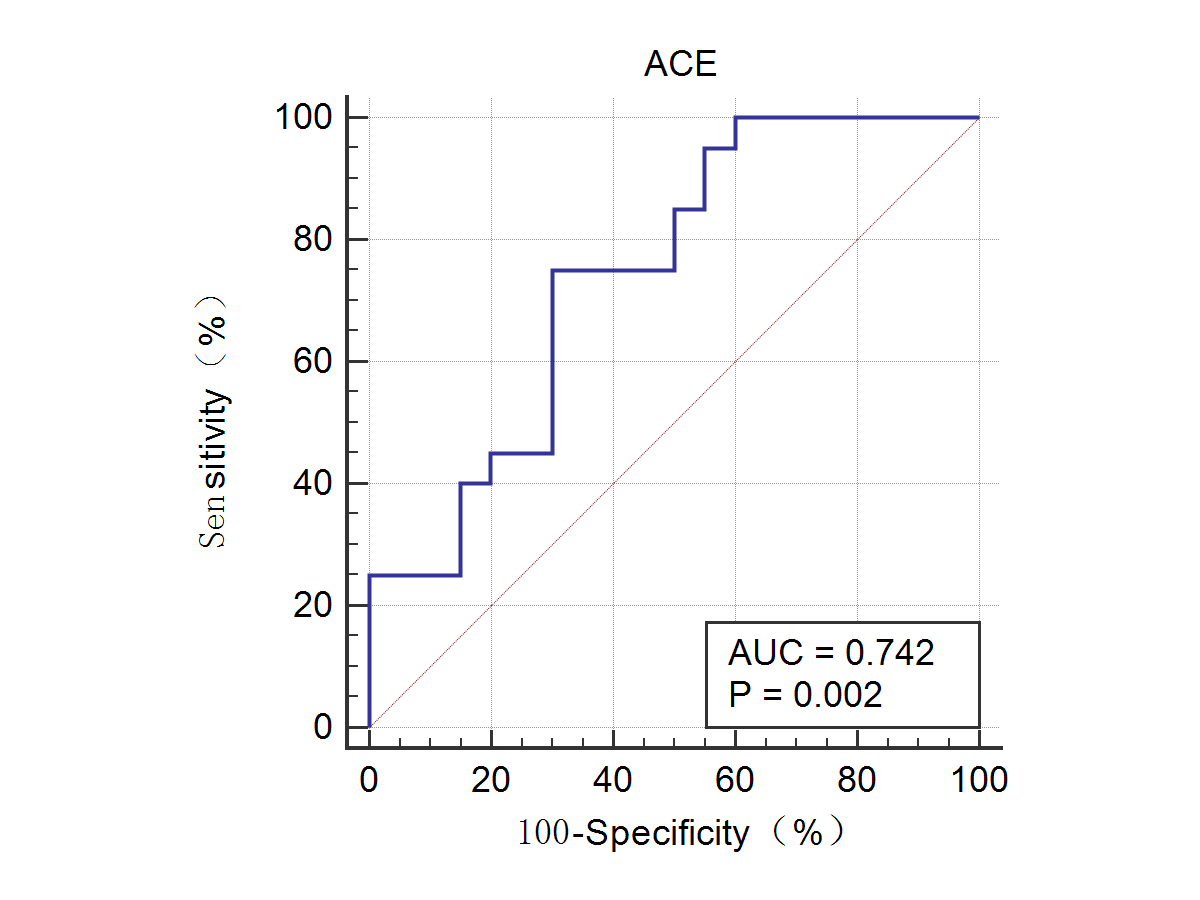

Supplement: Supplementary file 3 [file Data_Sheet_3.ZIP › raw data4/22.ROC/111016roc/ACE.png]

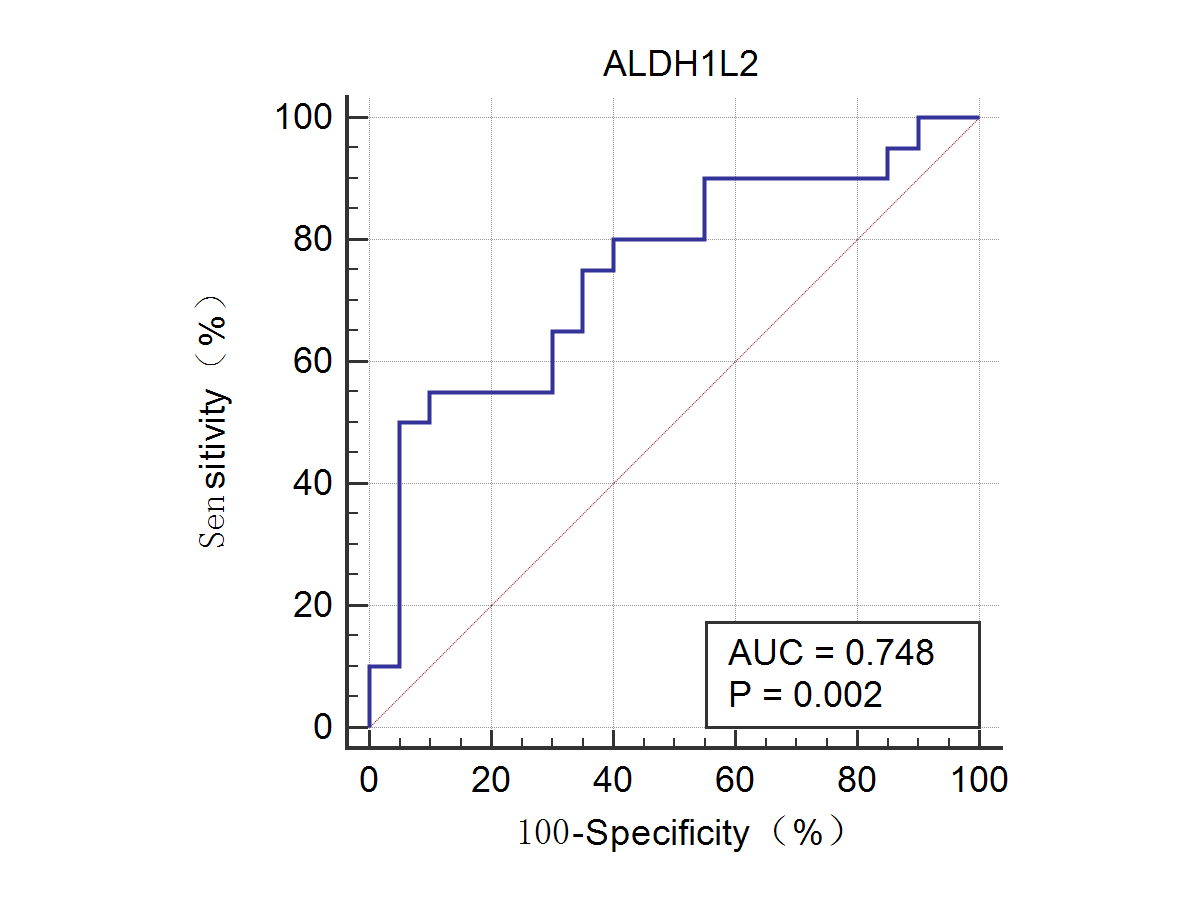

Supplement: Supplementary file 3 [file Data_Sheet_3.ZIP › raw data4/22.ROC/111016roc/ALDH1L2.png]

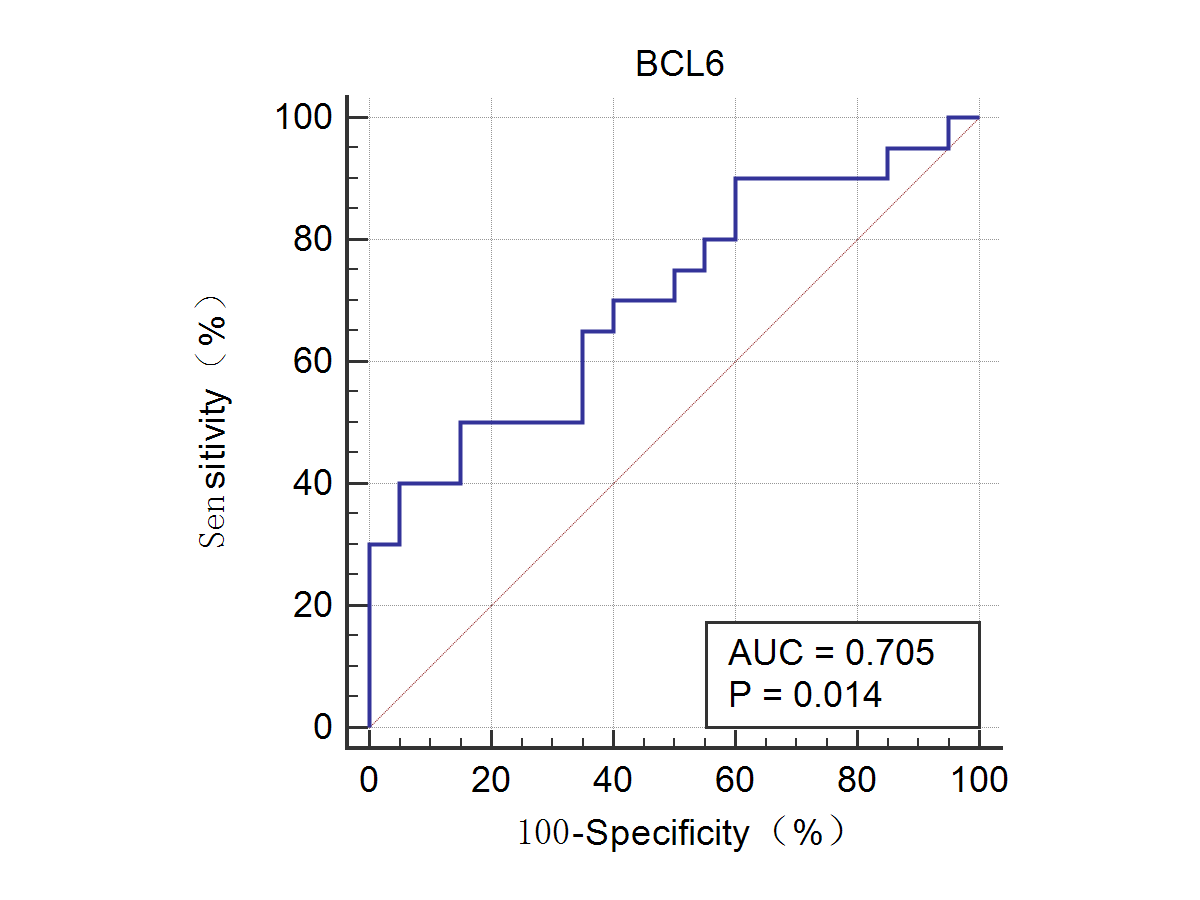

Supplement: Supplementary file 3 [file Data_Sheet_3.ZIP › raw data4/22.ROC/111016roc/BCL6.png]

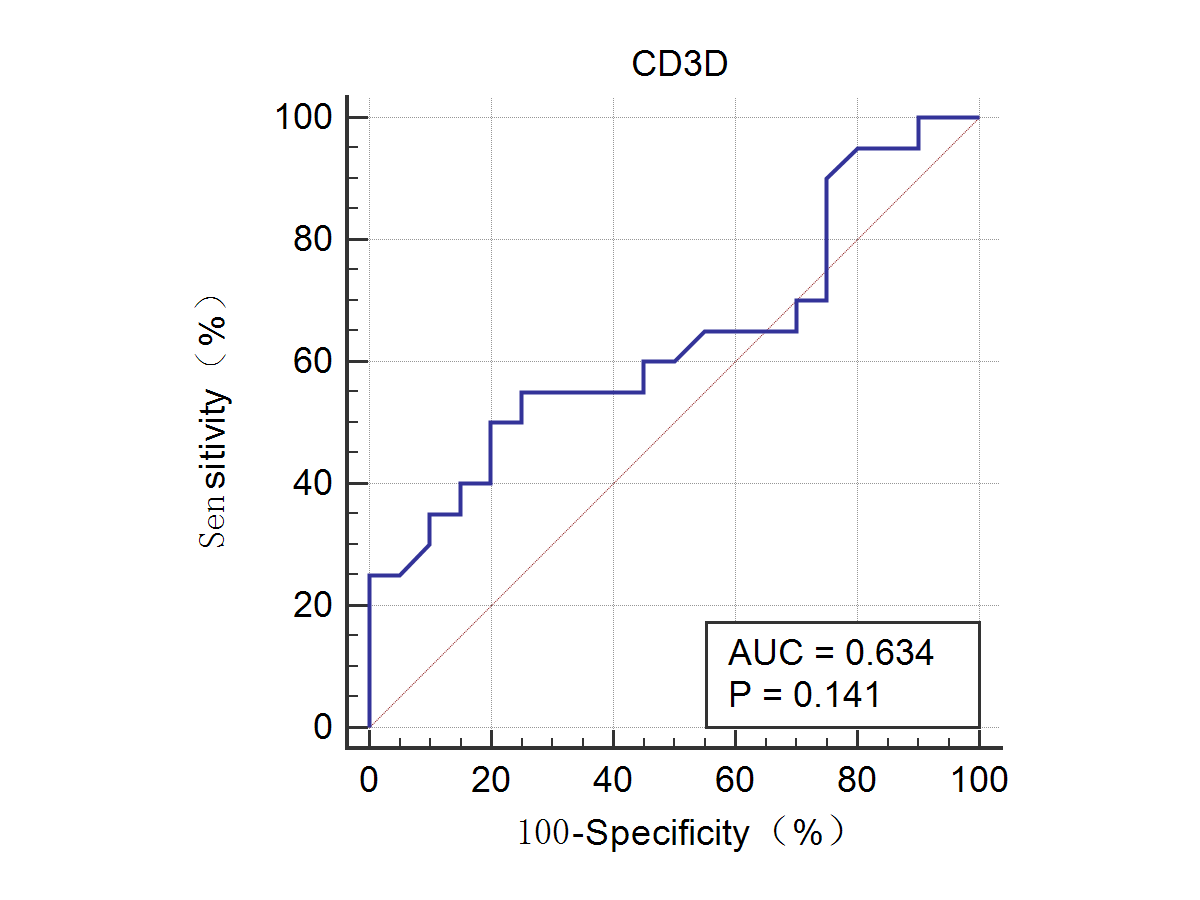

Supplement: Supplementary file 3 [file Data_Sheet_3.ZIP › raw data4/22.ROC/111016roc/CD3D.png]

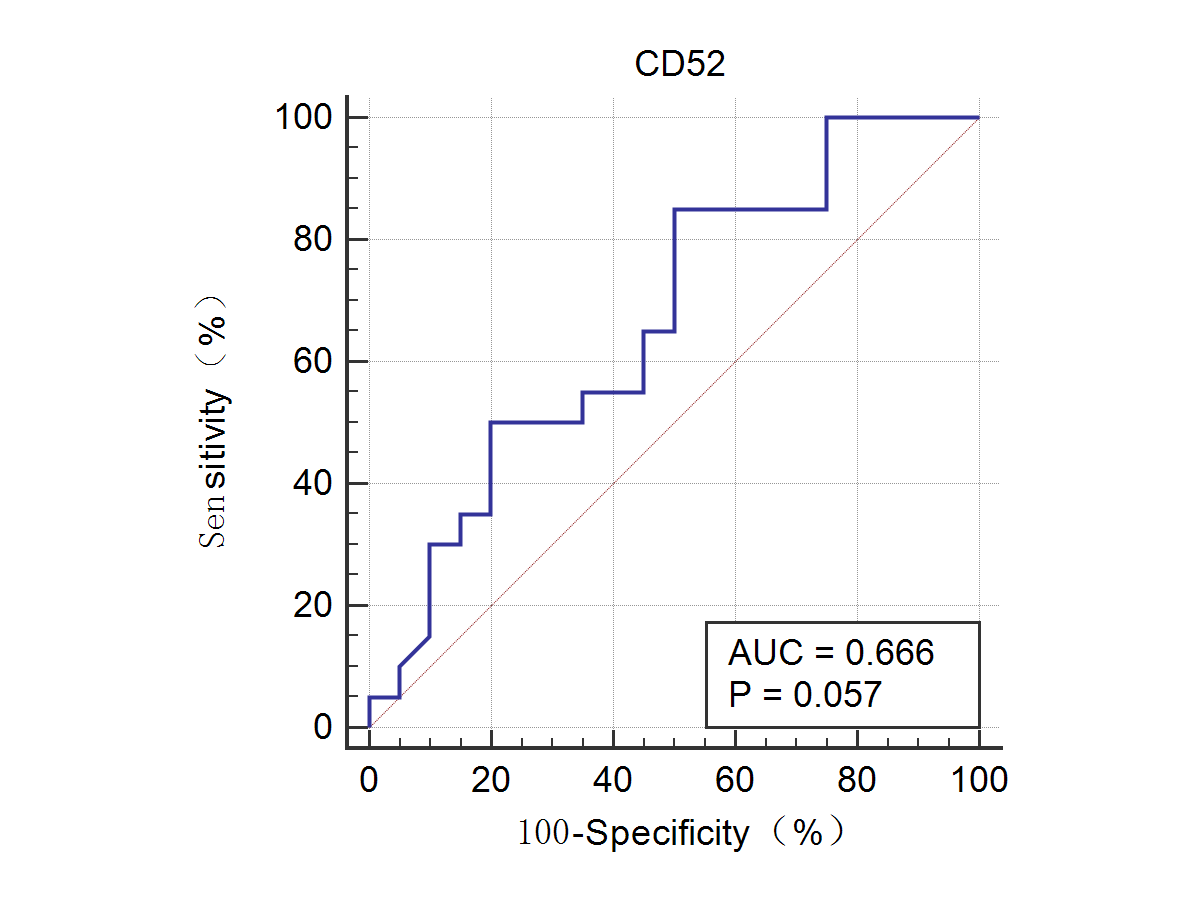

Supplement: Supplementary file 3 [file Data_Sheet_3.ZIP › raw data4/22.ROC/111016roc/CD52.png]

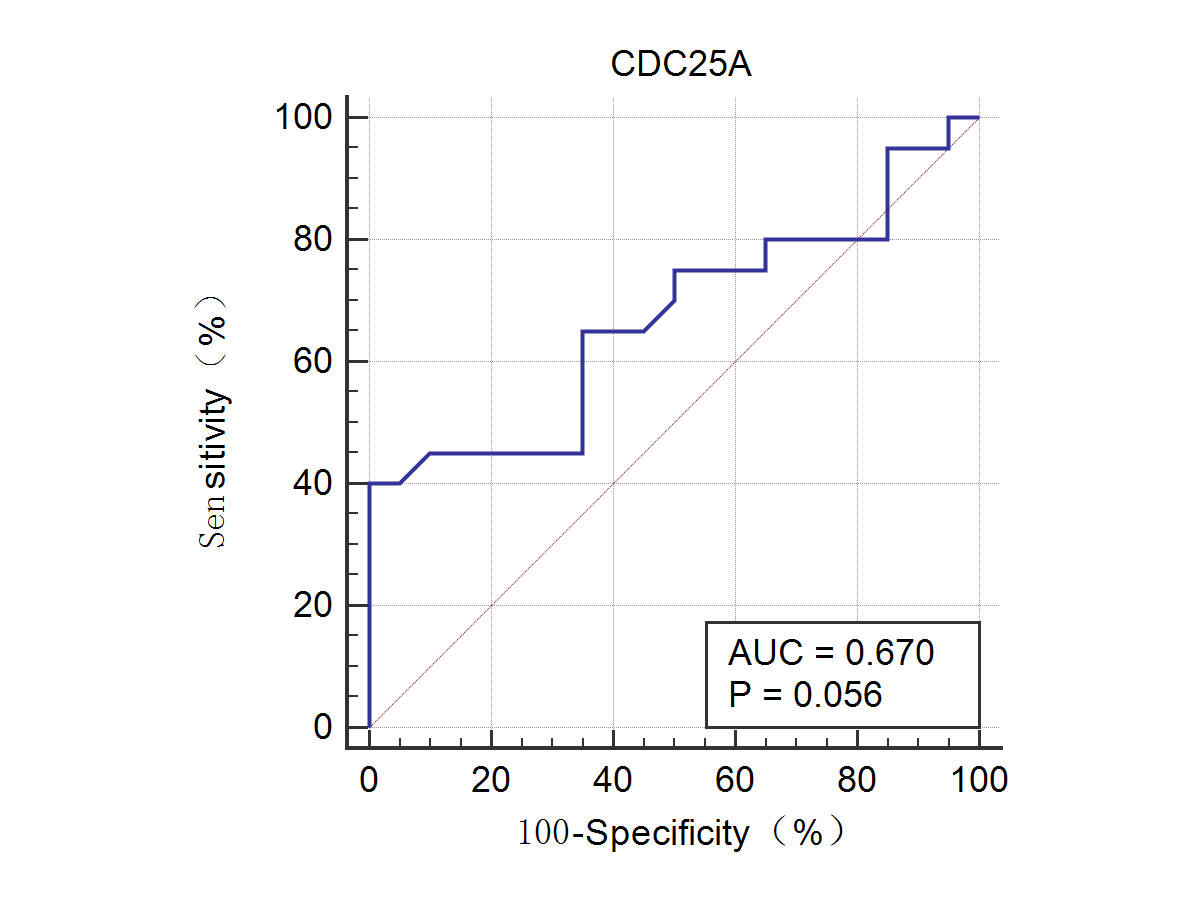

Supplement: Supplementary file 3 [file Data_Sheet_3.ZIP › raw data4/22.ROC/111016roc/CDC25A.png]

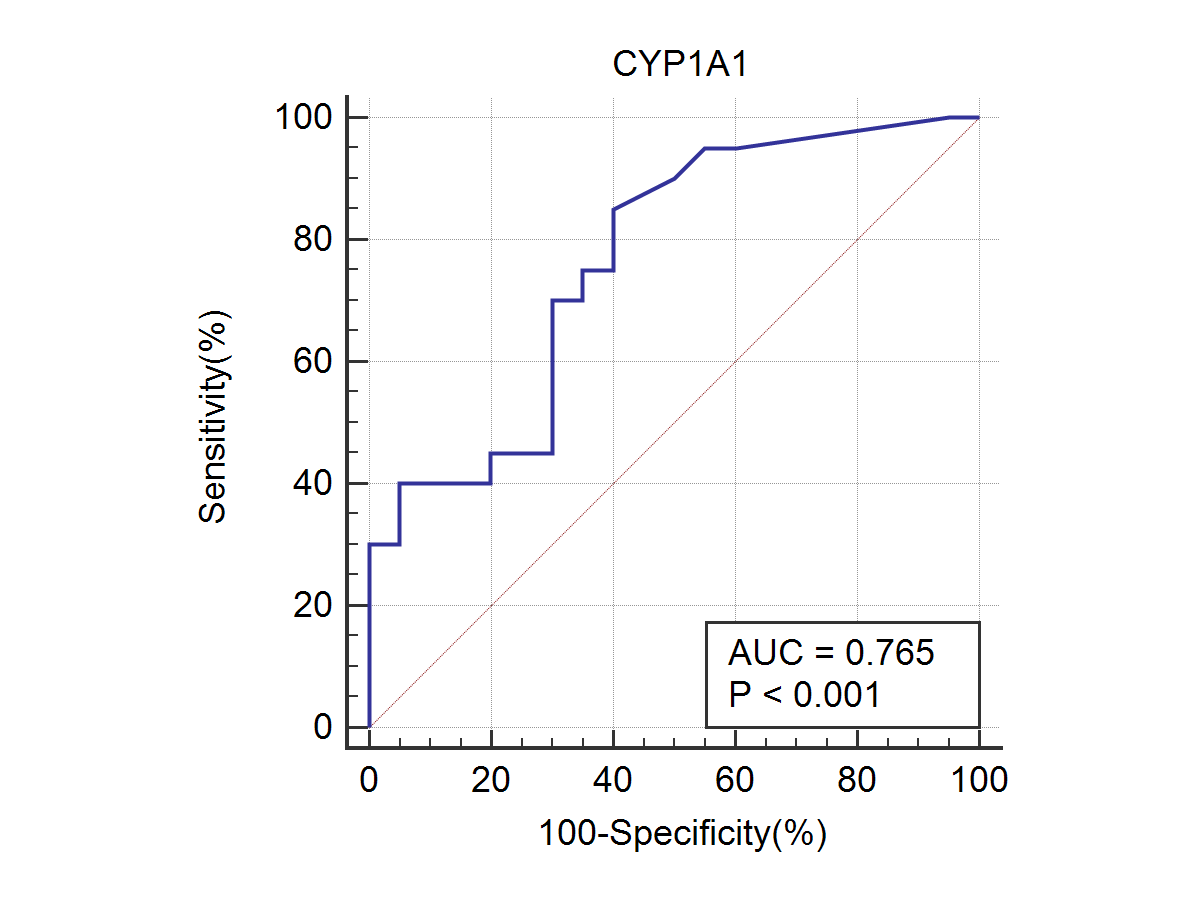

Supplement: Supplementary file 3 [file Data_Sheet_3.ZIP › raw data4/22.ROC/111016roc/CYP1A1.png]

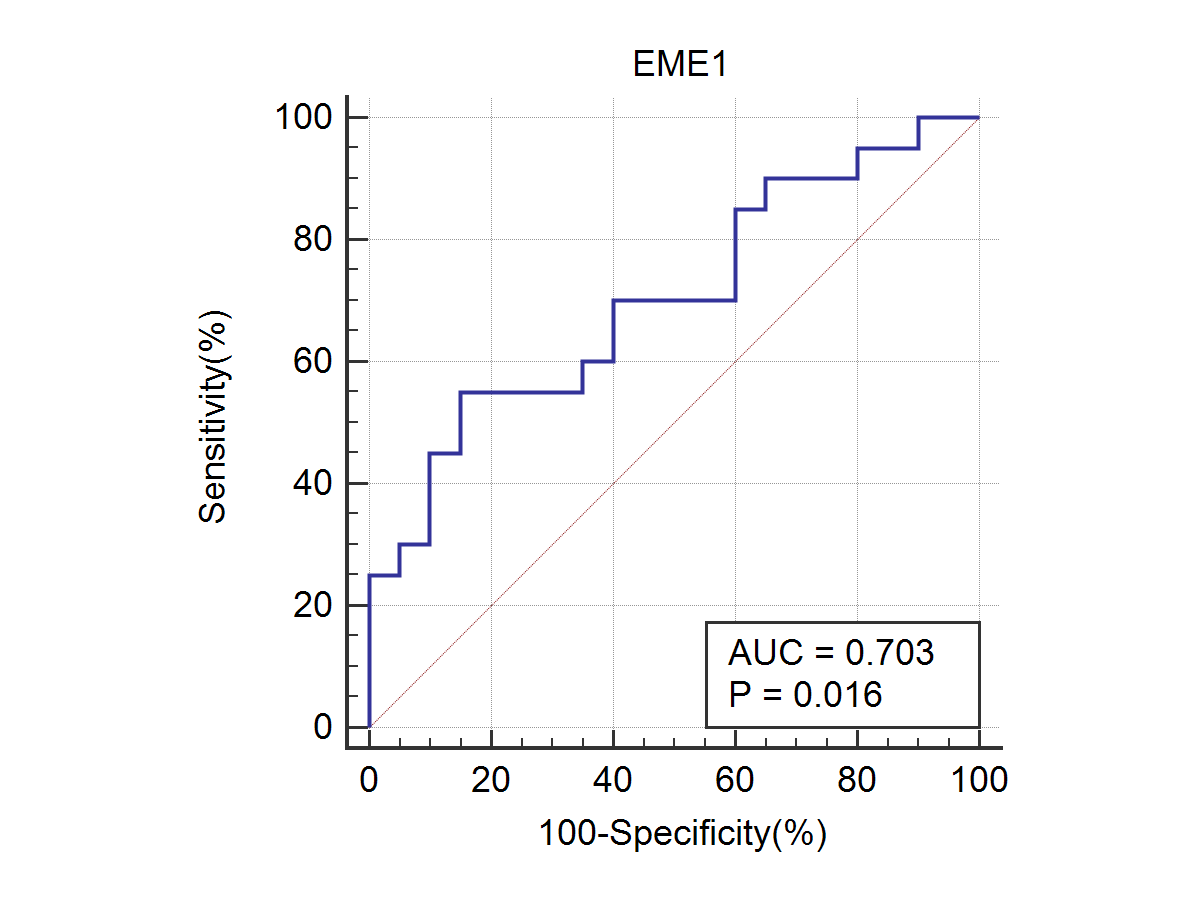

Supplement: Supplementary file 3 [file Data_Sheet_3.ZIP › raw data4/22.ROC/111016roc/EME1.png]

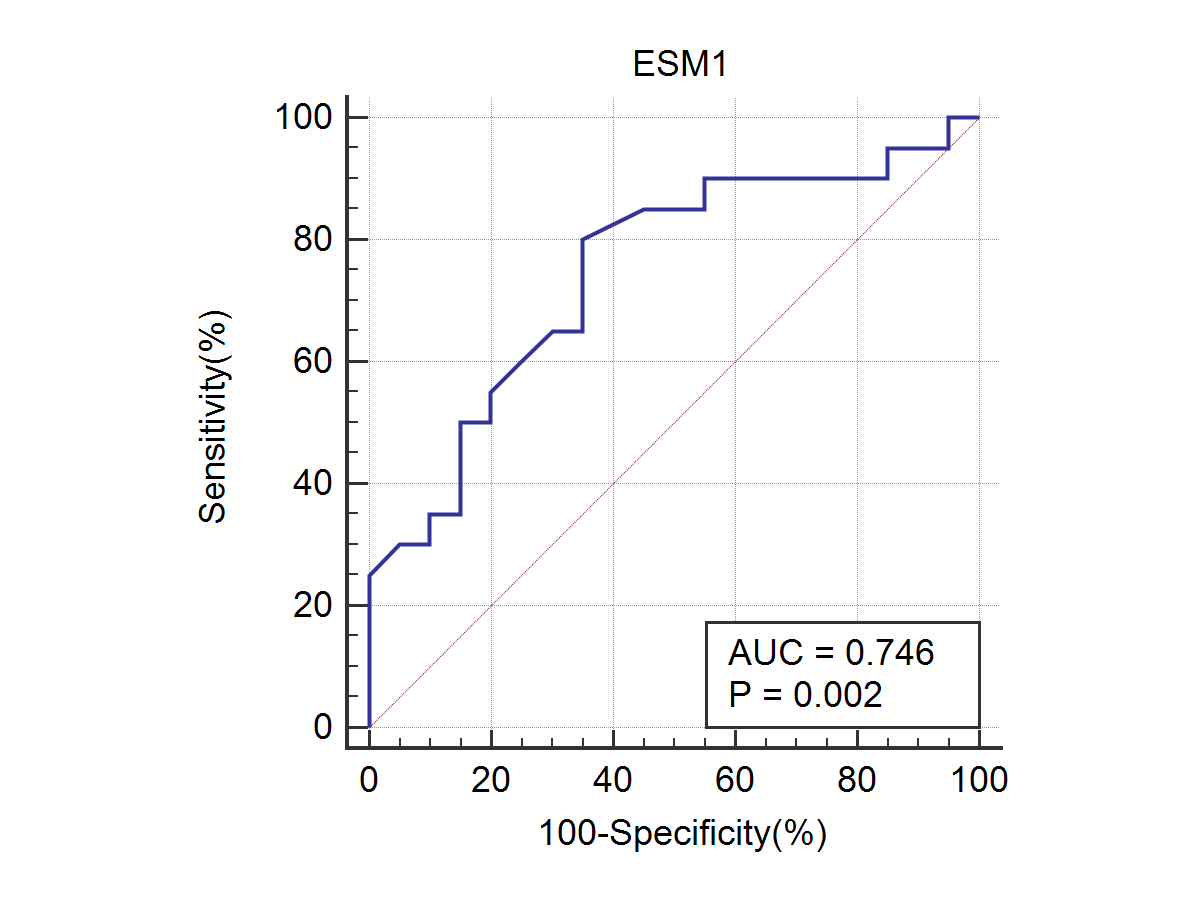

Supplement: Supplementary file 3 [file Data_Sheet_3.ZIP › raw data4/22.ROC/111016roc/ESM1.png]

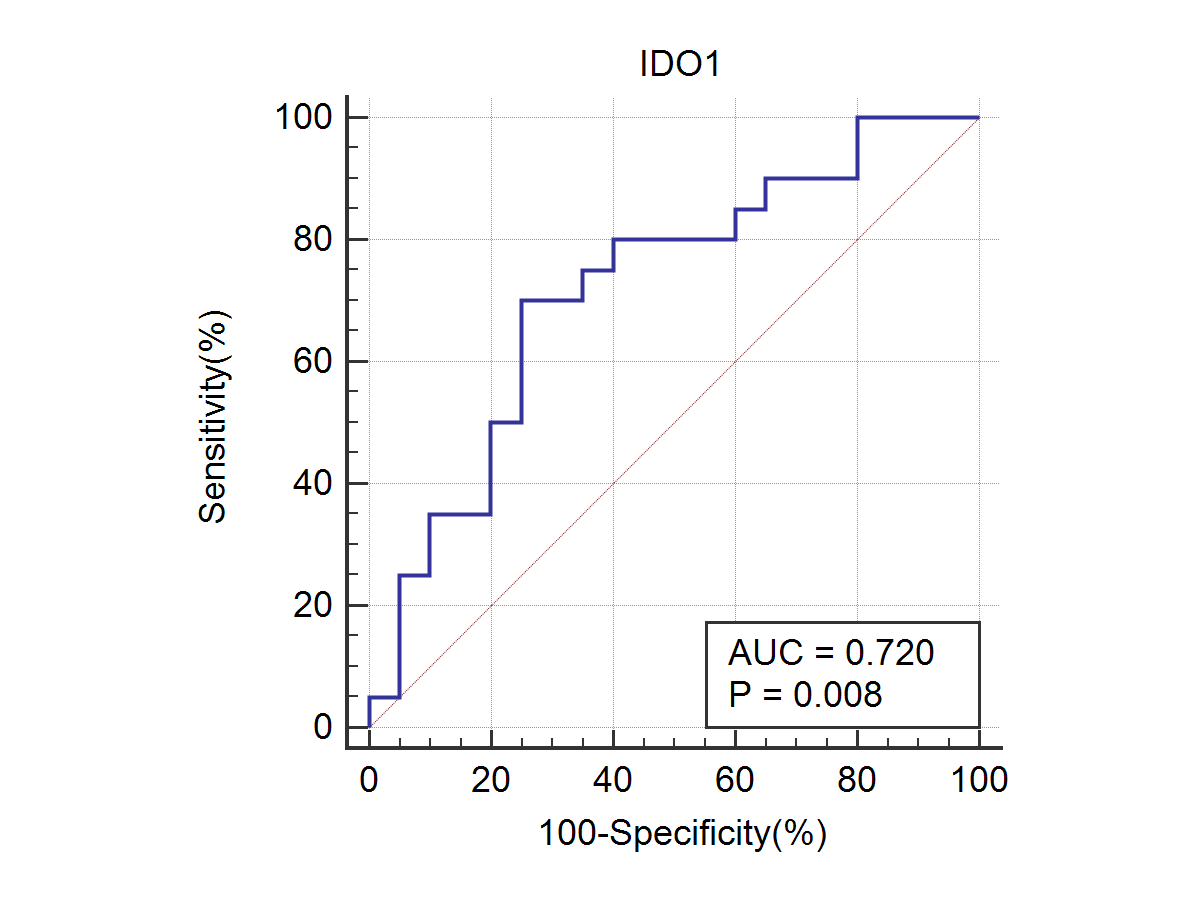

Supplement: Supplementary file 3 [file Data_Sheet_3.ZIP › raw data4/22.ROC/111016roc/IDO1.png]

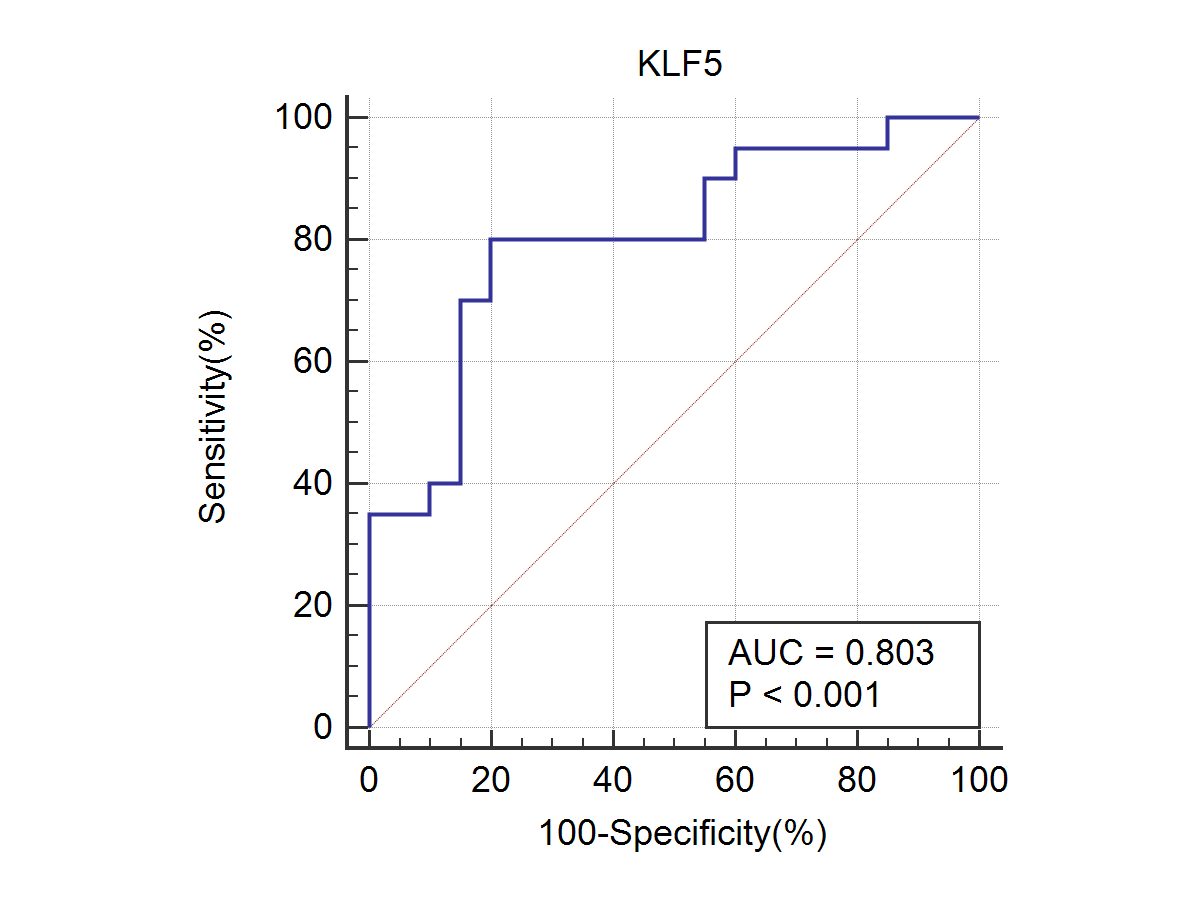

Supplement: Supplementary file 3 [file Data_Sheet_3.ZIP › raw data4/22.ROC/111016roc/KLF5.png]

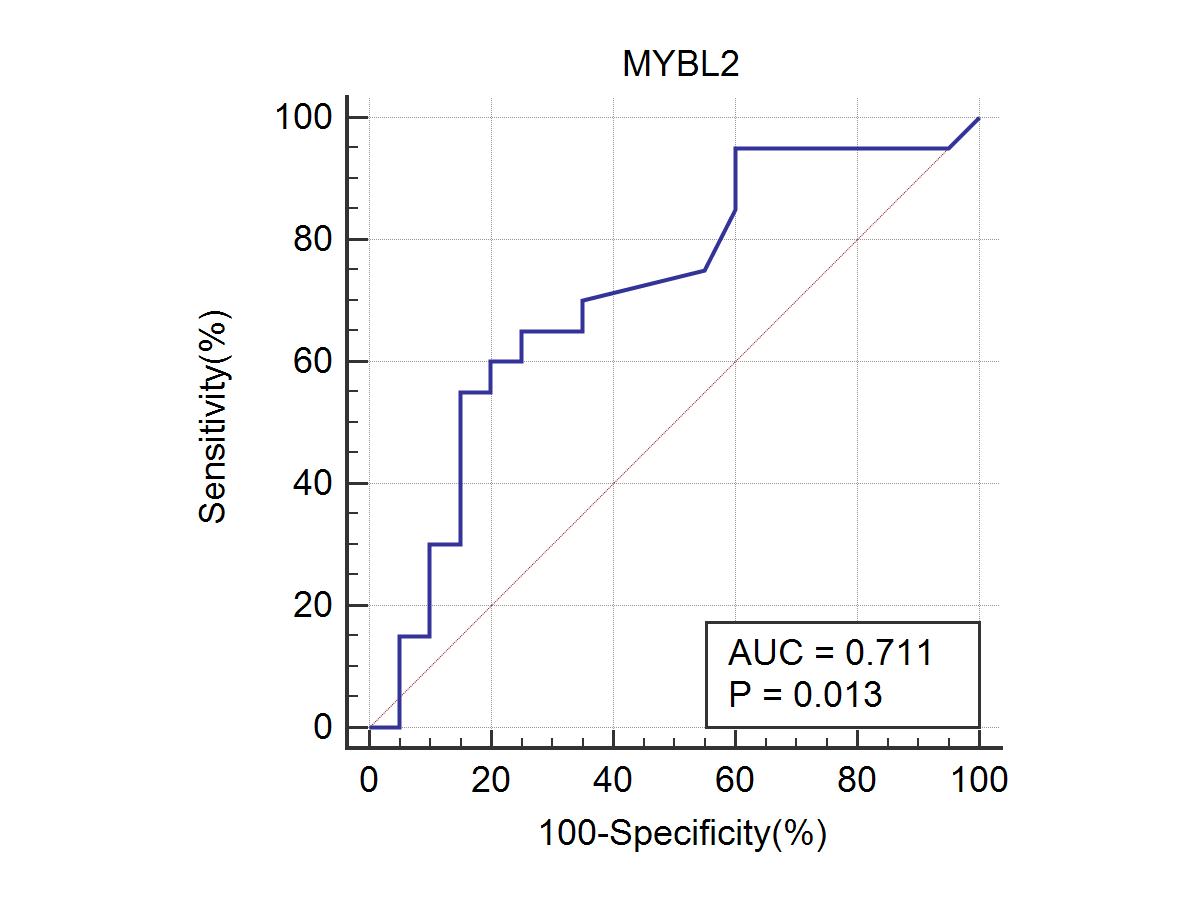

Supplement: Supplementary file 3 [file Data_Sheet_3.ZIP › raw data4/22.ROC/111016roc/MYBL2.png]

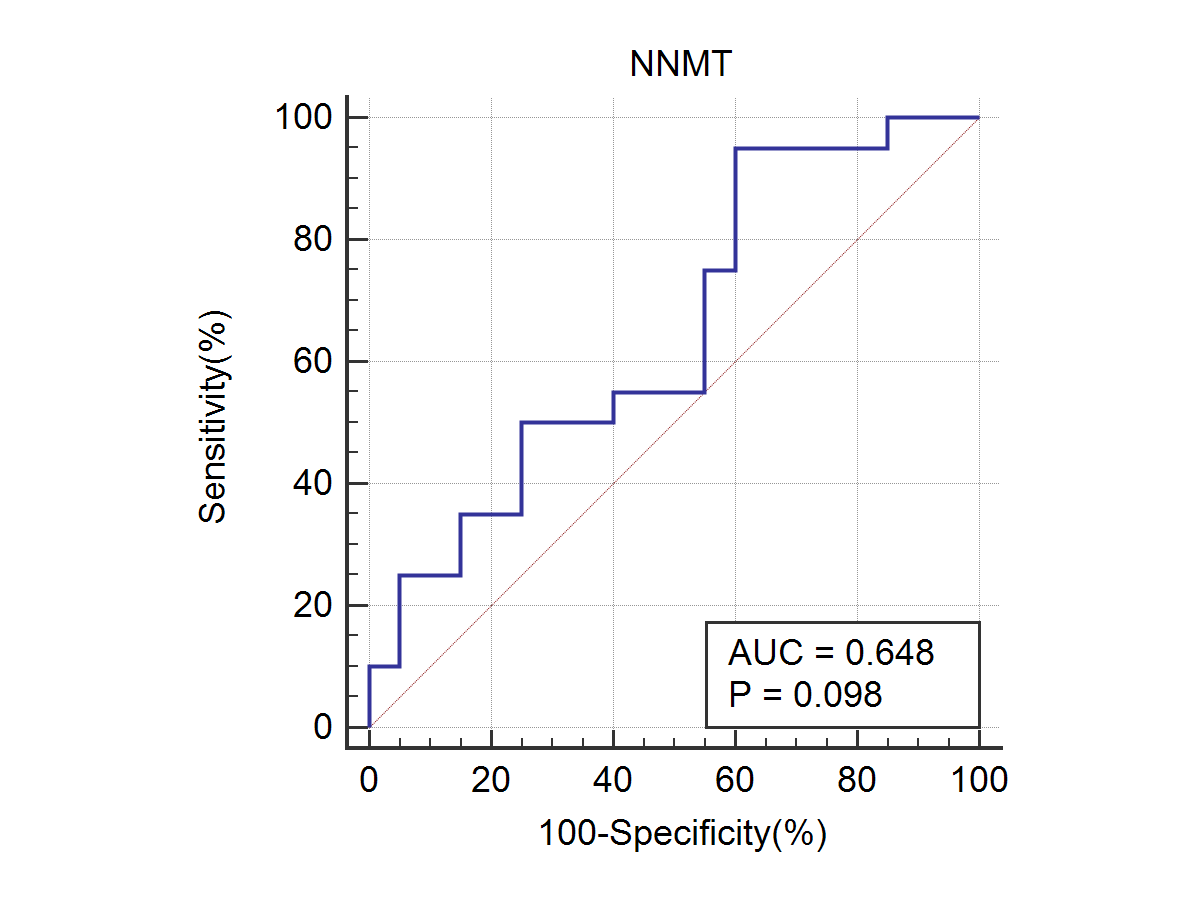

Supplement: Supplementary file 3 [file Data_Sheet_3.ZIP › raw data4/22.ROC/111016roc/NNMT.png]

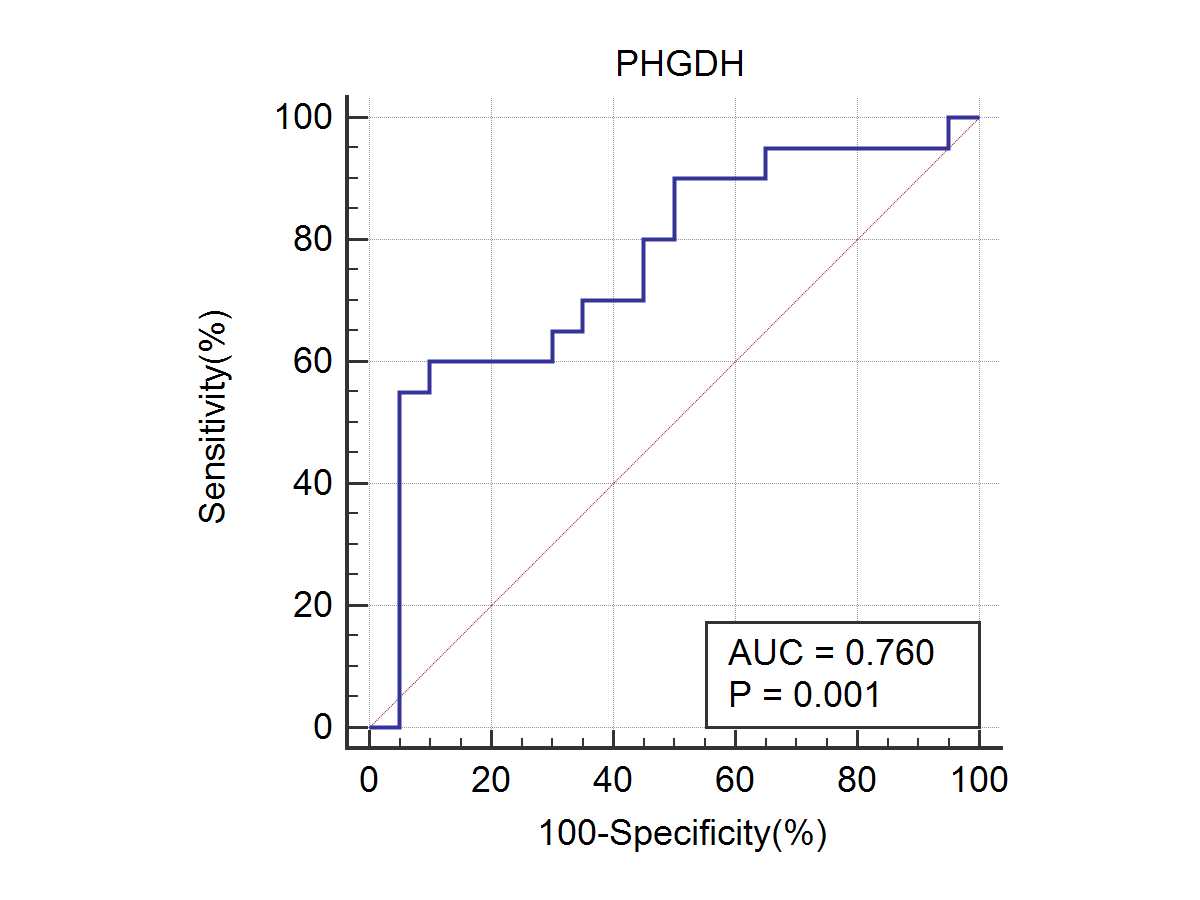

Supplement: Supplementary file 3 [file Data_Sheet_3.ZIP › raw data4/22.ROC/111016roc/PHGDH.png]

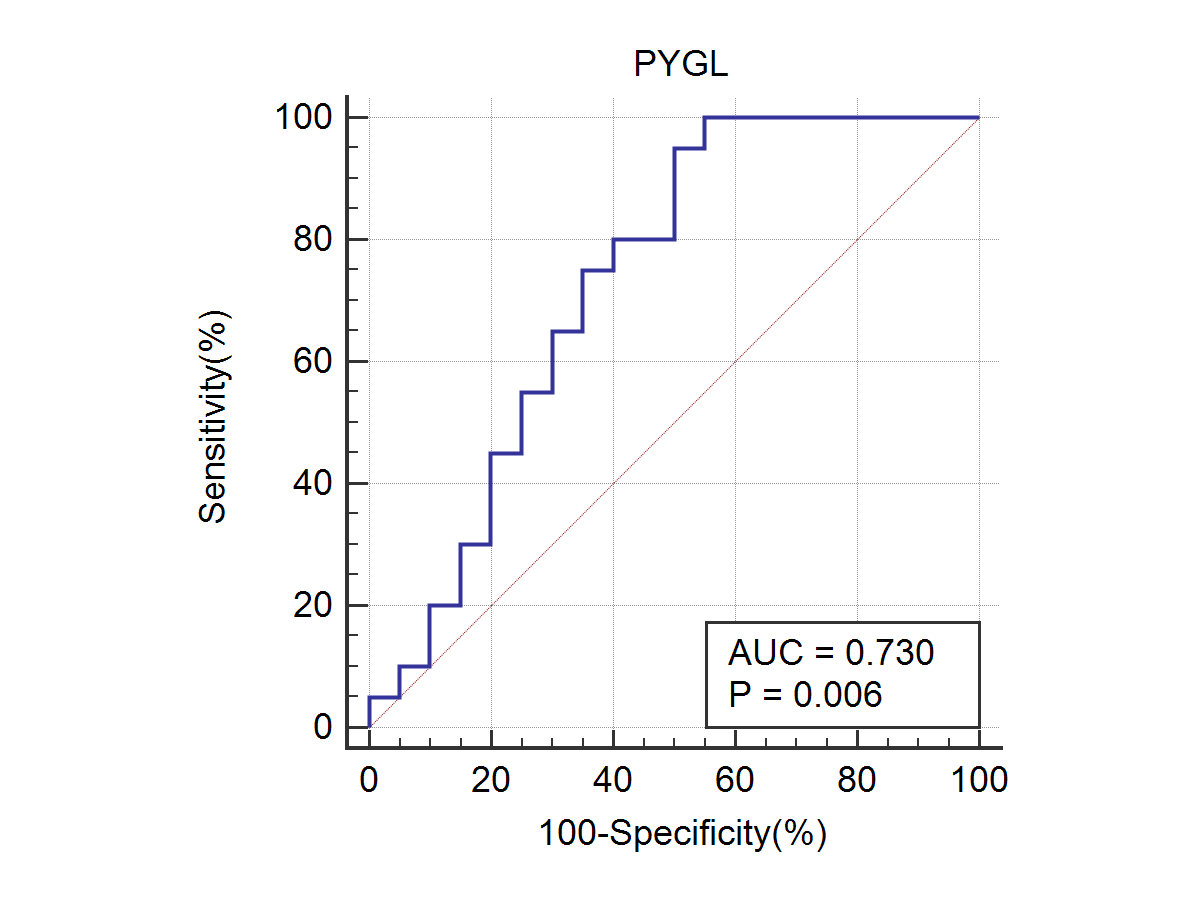

Supplement: Supplementary file 3 [file Data_Sheet_3.ZIP › raw data4/22.ROC/111016roc/PYGL.png]

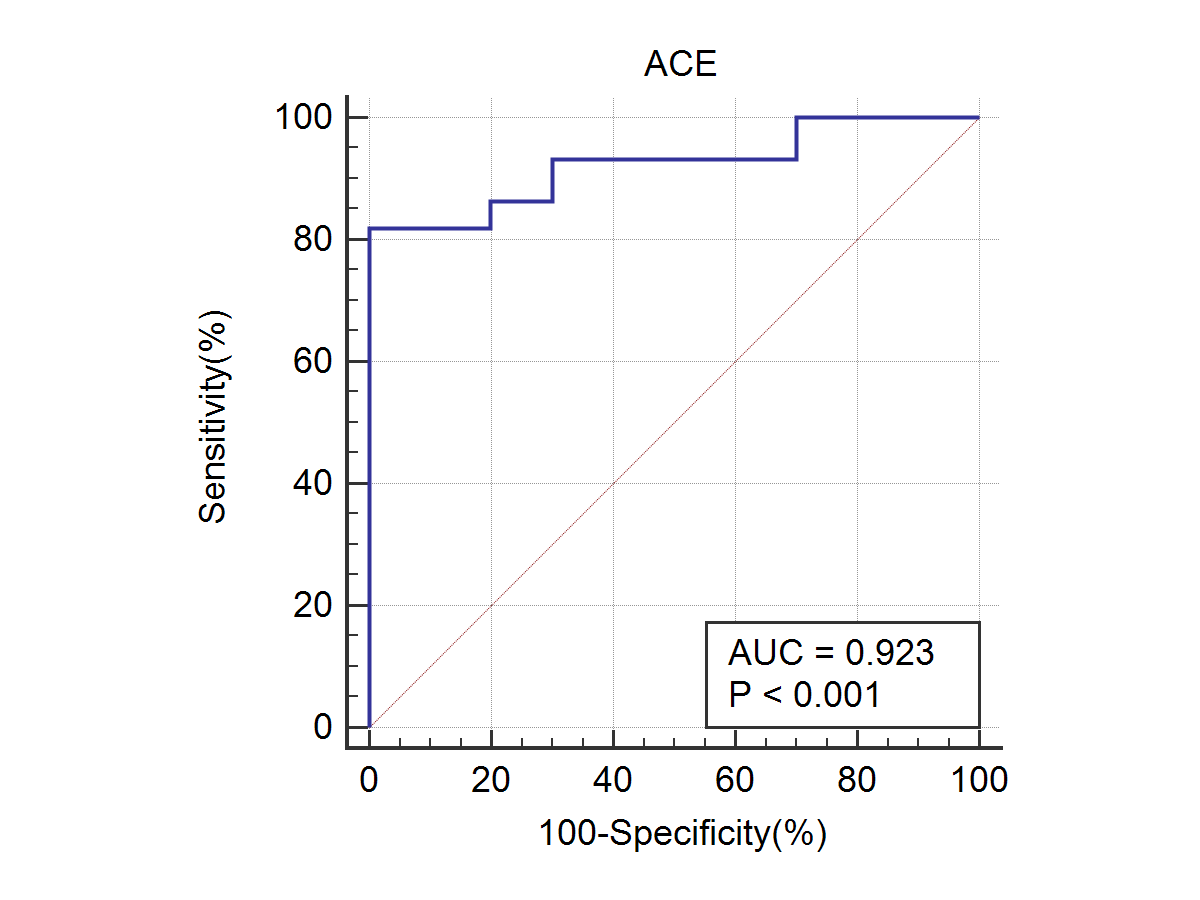

Supplement: Supplementary file 3 [file Data_Sheet_3.ZIP › raw data4/22.ROC/171110roc/ACE.png]

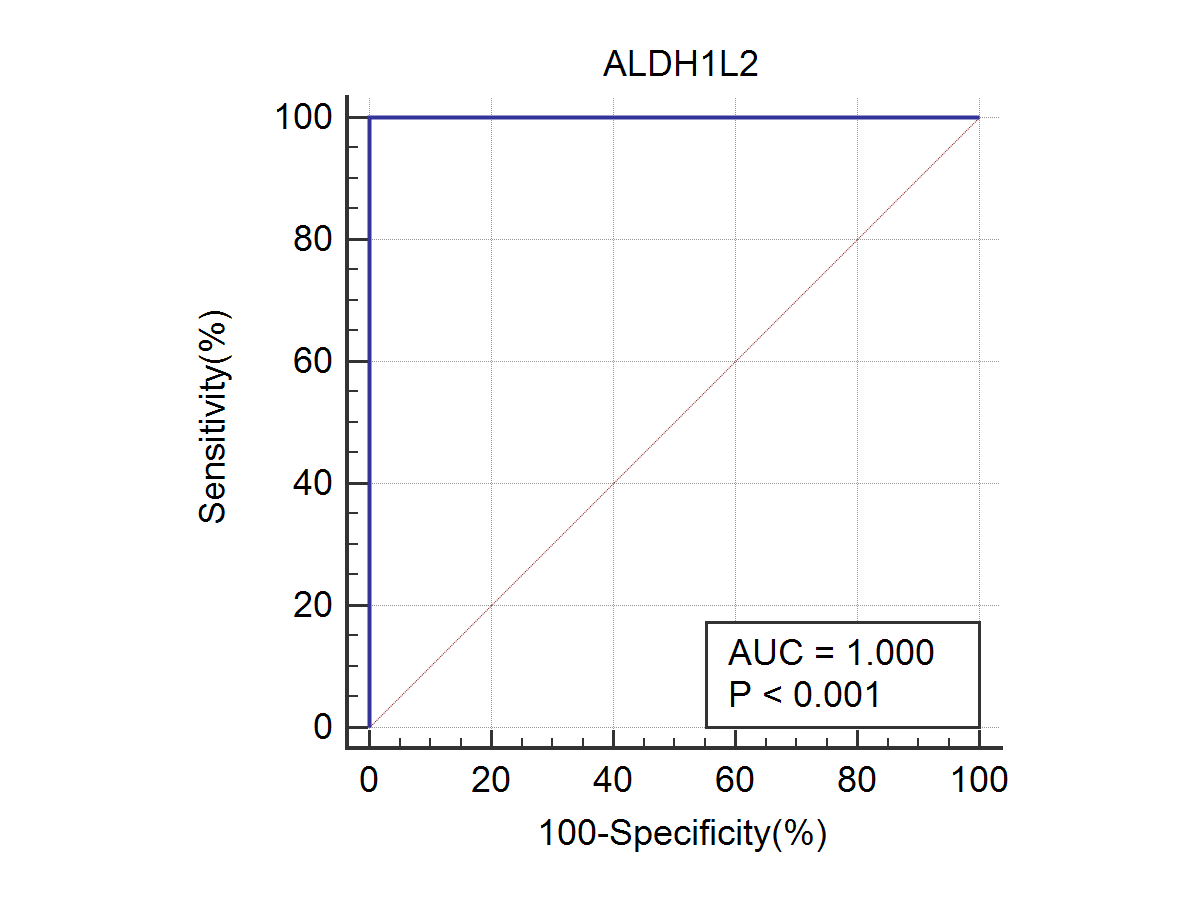

Supplement: Supplementary file 3 [file Data_Sheet_3.ZIP › raw data4/22.ROC/171110roc/ALDH1L2.png]

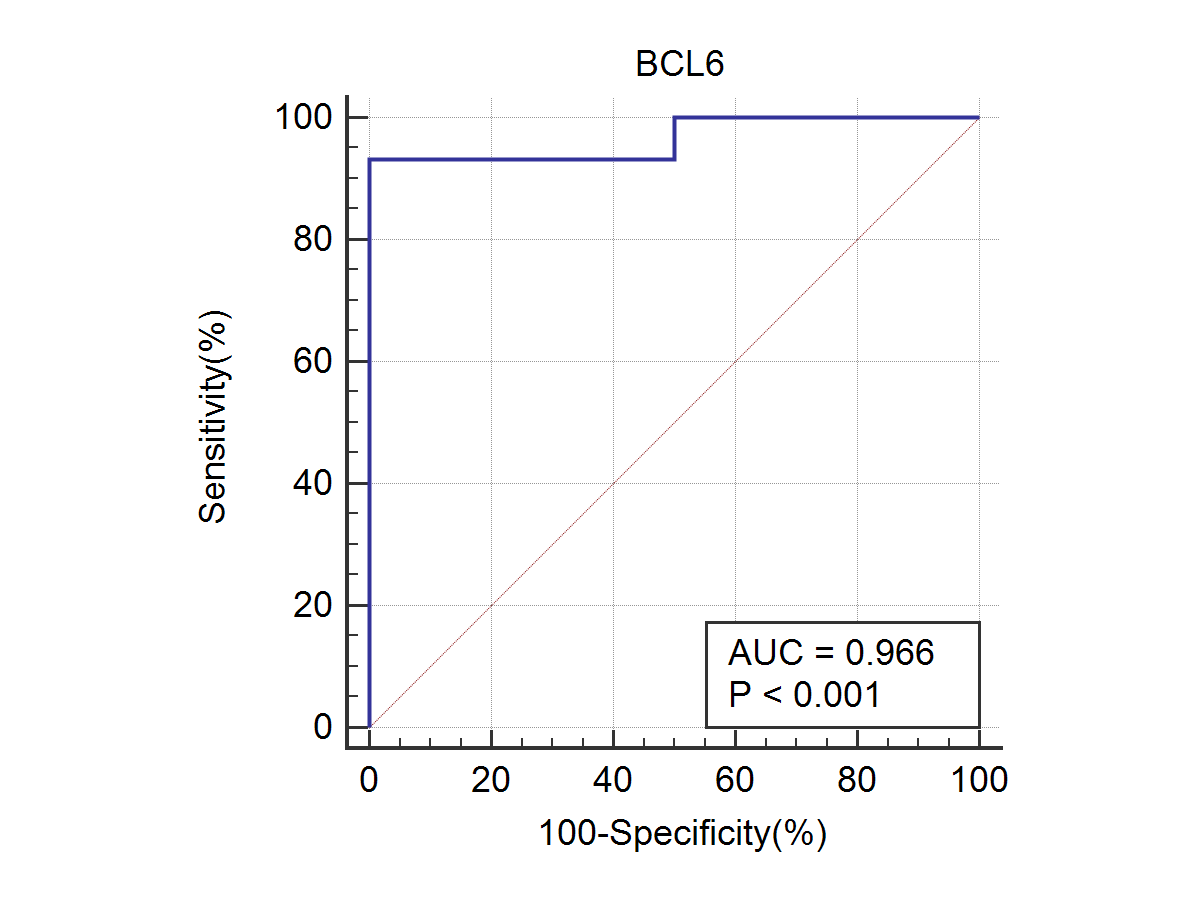

Supplement: Supplementary file 3 [file Data_Sheet_3.ZIP › raw data4/22.ROC/171110roc/BCL6.png]

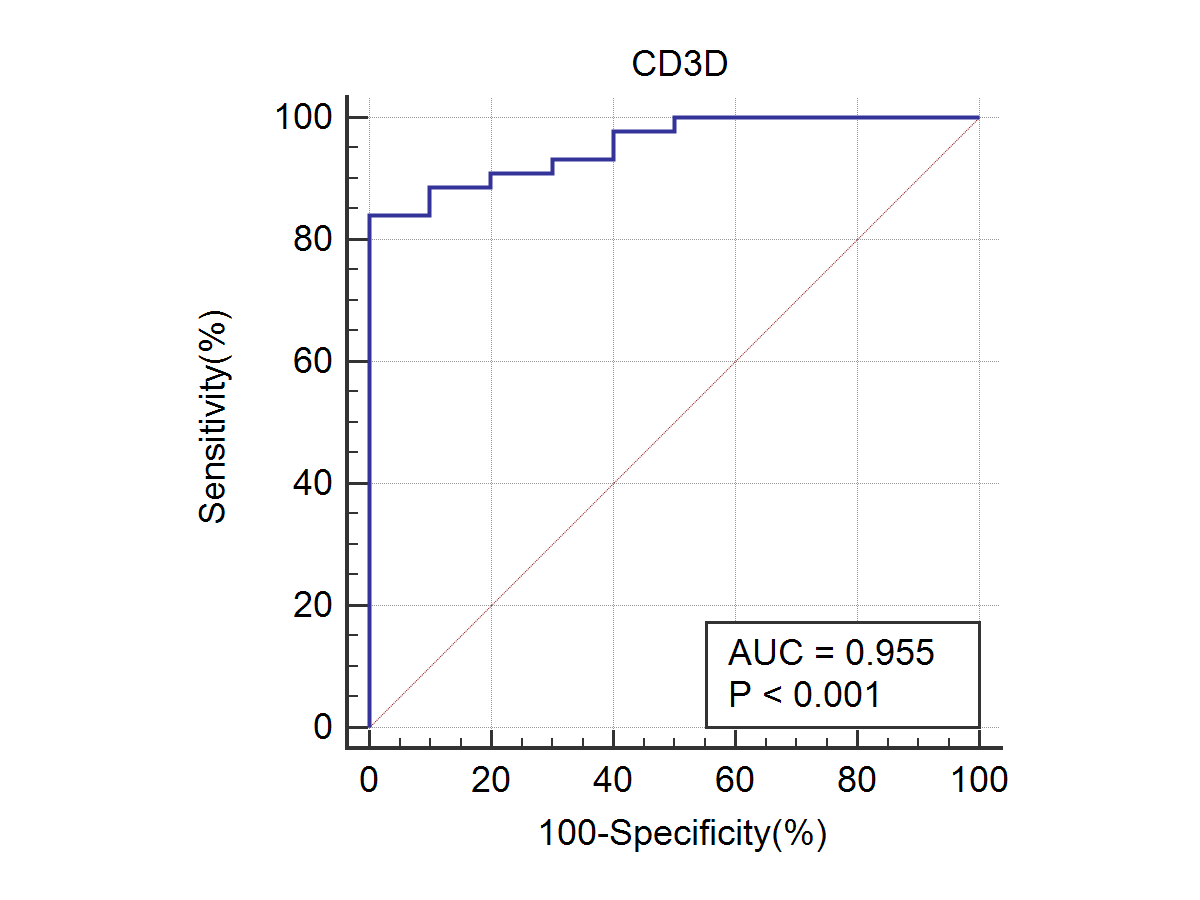

Supplement: Supplementary file 3 [file Data_Sheet_3.ZIP › raw data4/22.ROC/171110roc/CD3D.png]

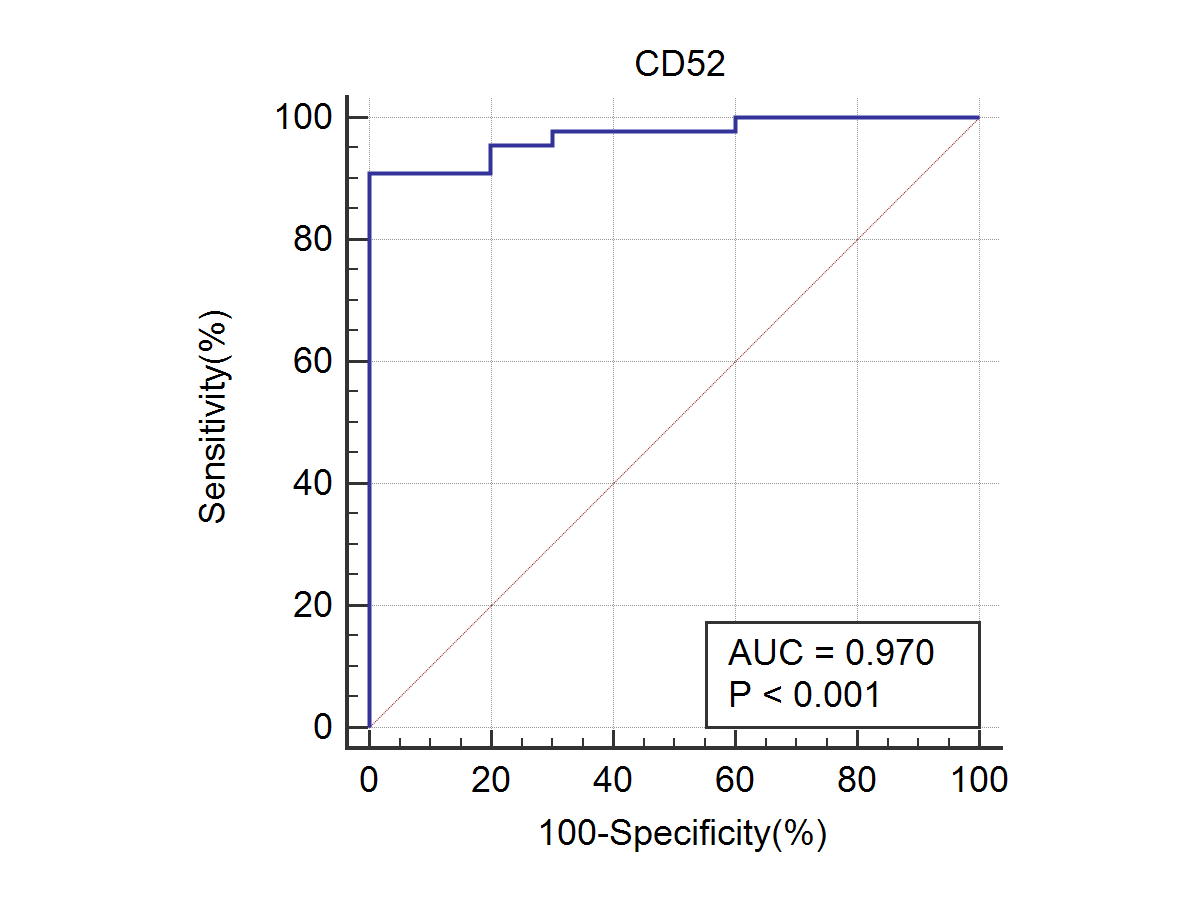

Supplement: Supplementary file 3 [file Data_Sheet_3.ZIP › raw data4/22.ROC/171110roc/CD52.png]

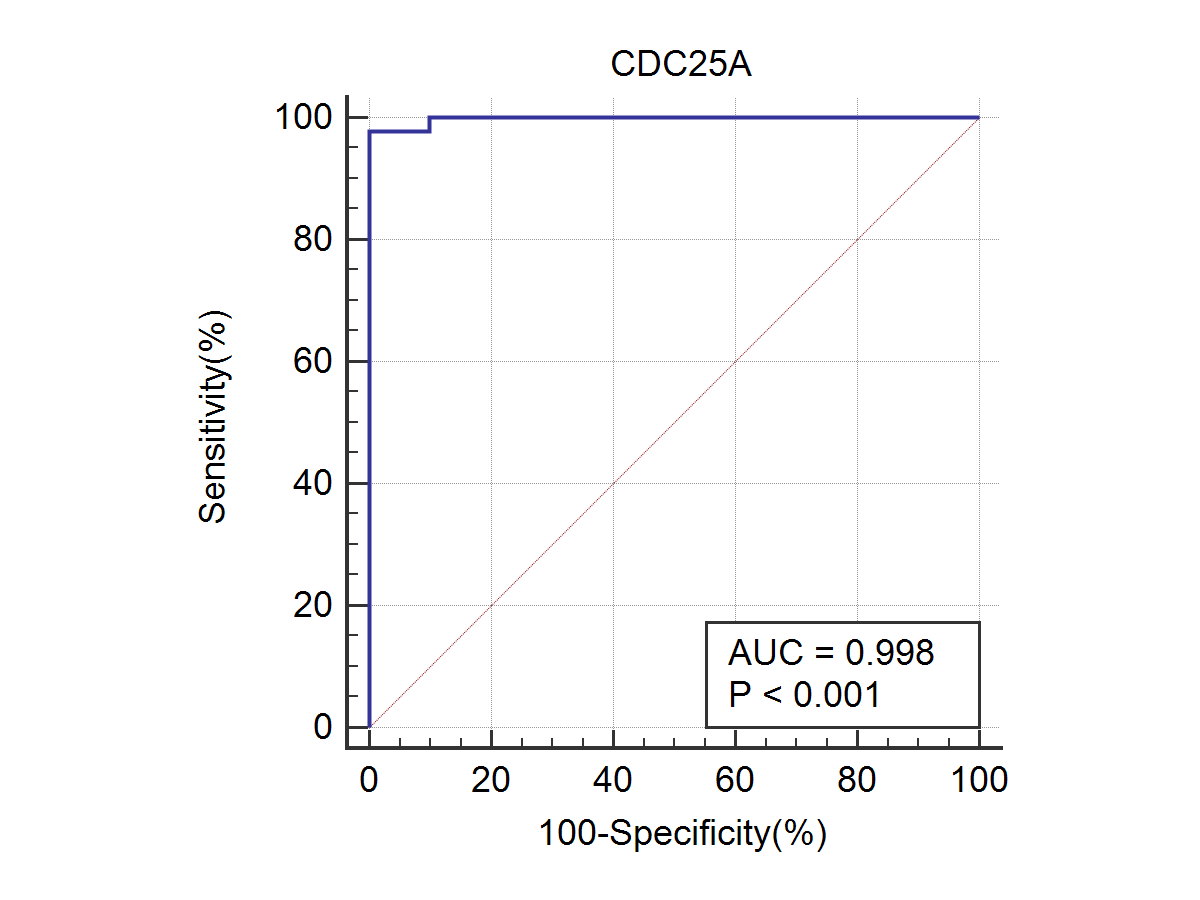

Supplement: Supplementary file 3 [file Data_Sheet_3.ZIP › raw data4/22.ROC/171110roc/CDC25A.png]

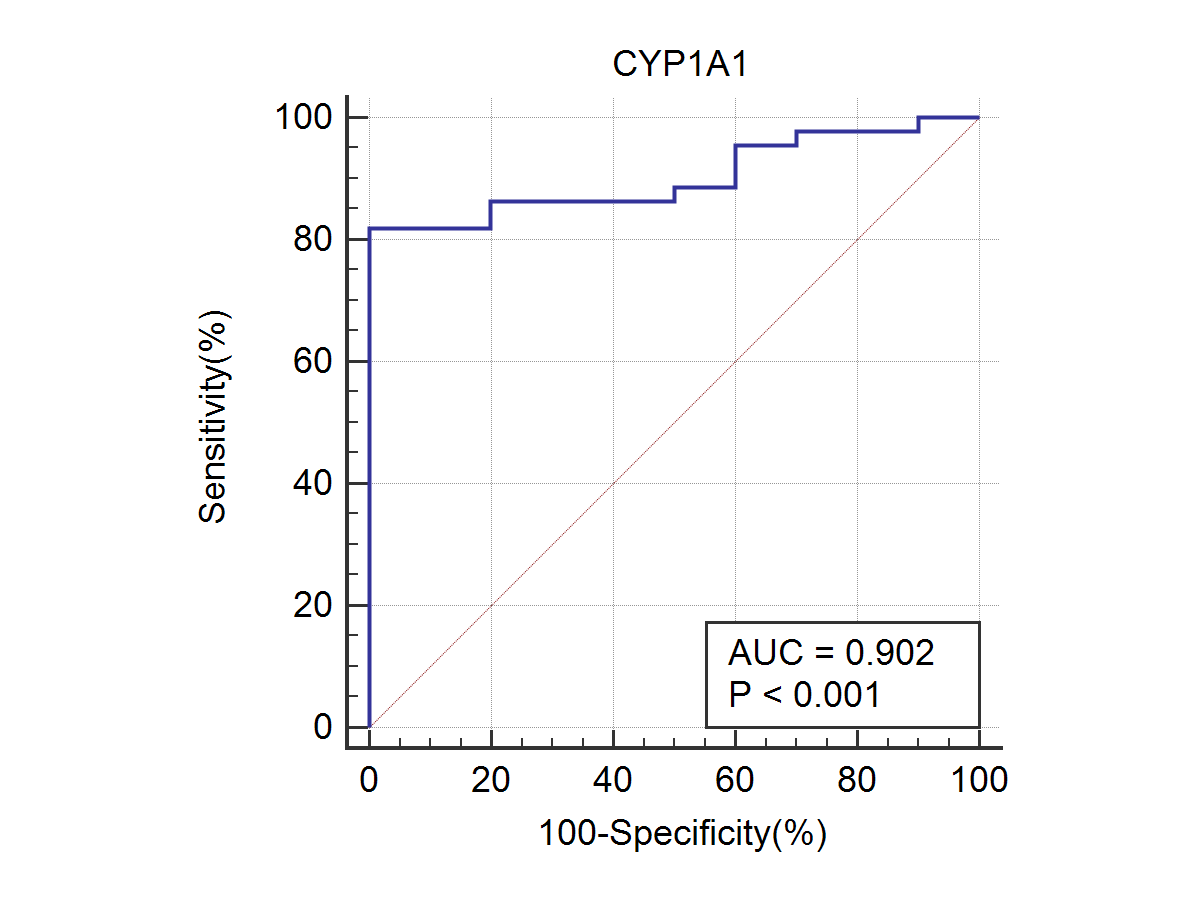

Supplement: Supplementary file 3 [file Data_Sheet_3.ZIP › raw data4/22.ROC/171110roc/CYP1A1.png]

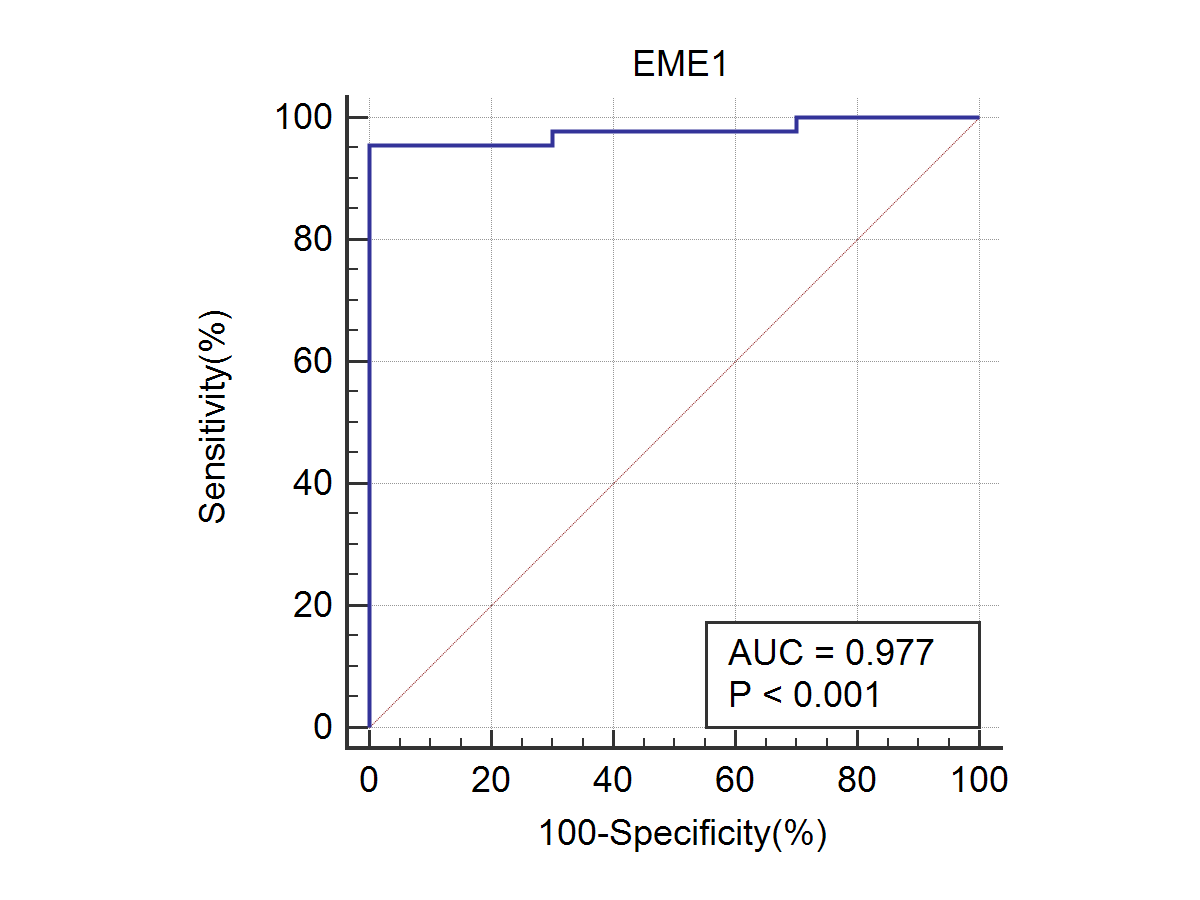

Supplement: Supplementary file 3 [file Data_Sheet_3.ZIP › raw data4/22.ROC/171110roc/EME1.png]

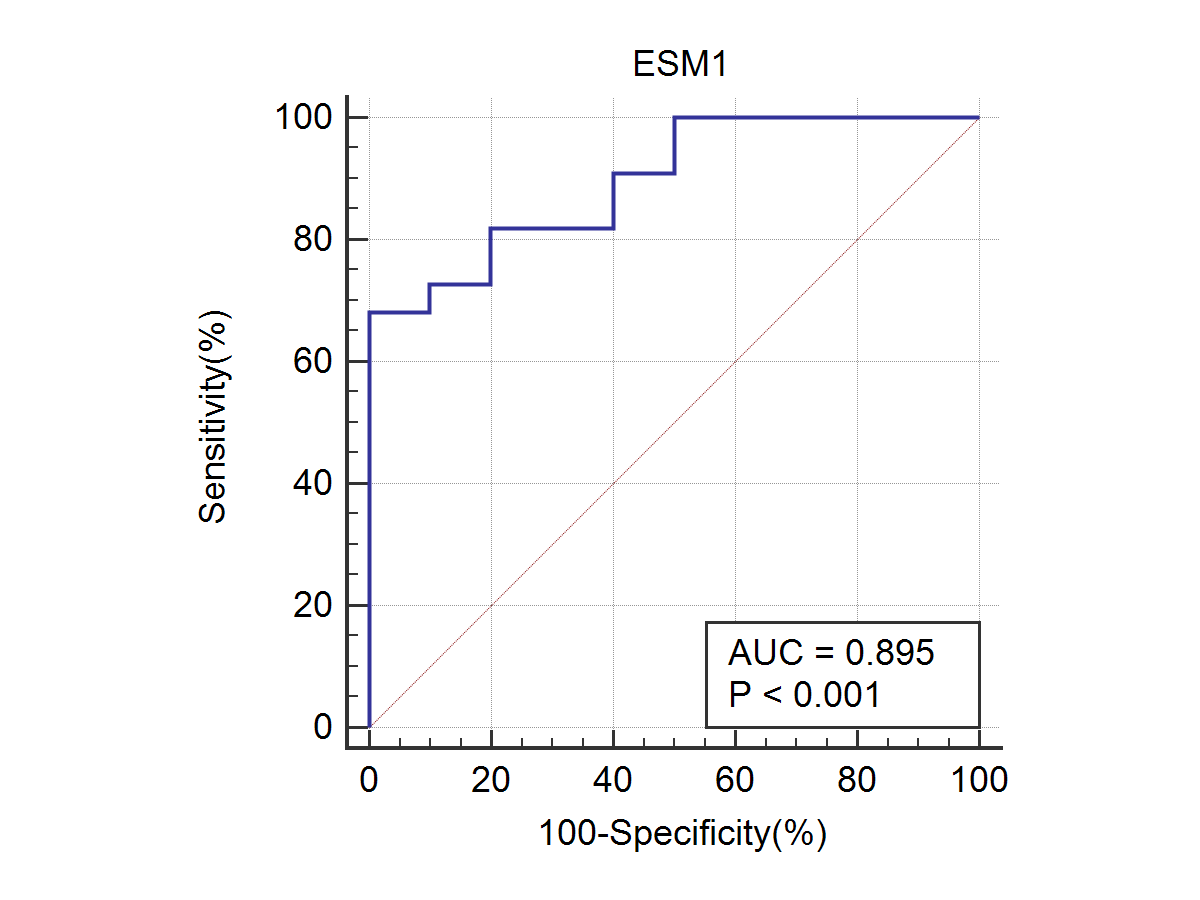

Supplement: Supplementary file 3 [file Data_Sheet_3.ZIP › raw data4/22.ROC/171110roc/ESM1.png]

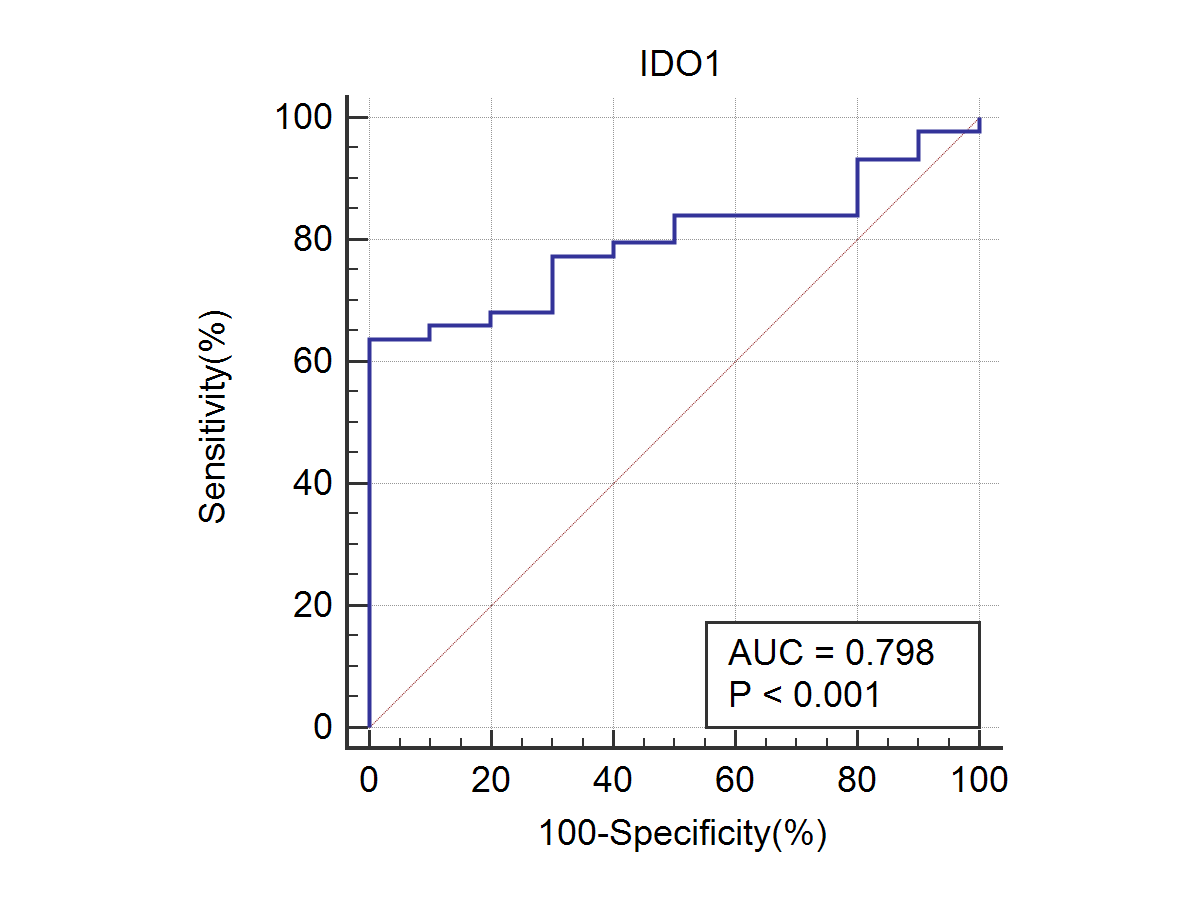

Supplement: Supplementary file 3 [file Data_Sheet_3.ZIP › raw data4/22.ROC/171110roc/IDO1.png]

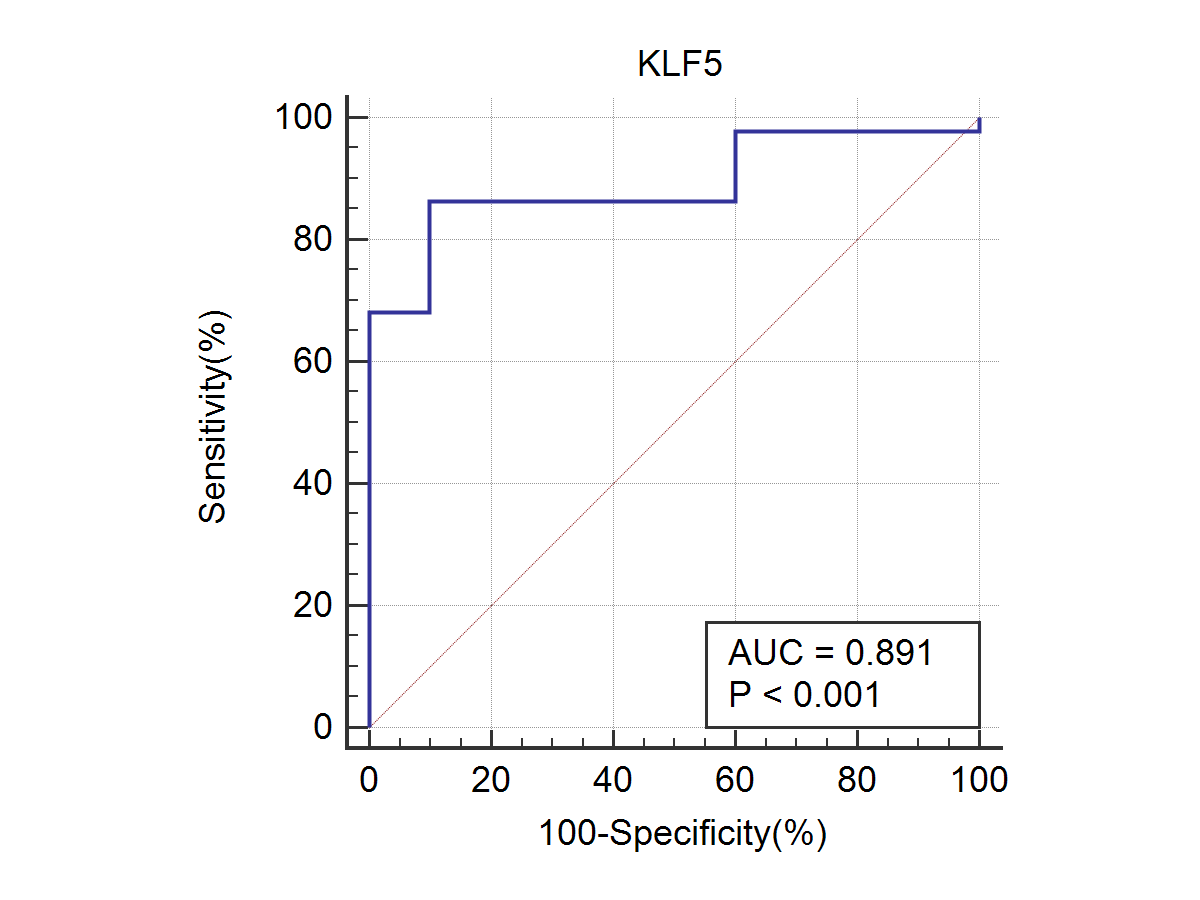

Supplement: Supplementary file 3 [file Data_Sheet_3.ZIP › raw data4/22.ROC/171110roc/KLF5.png]

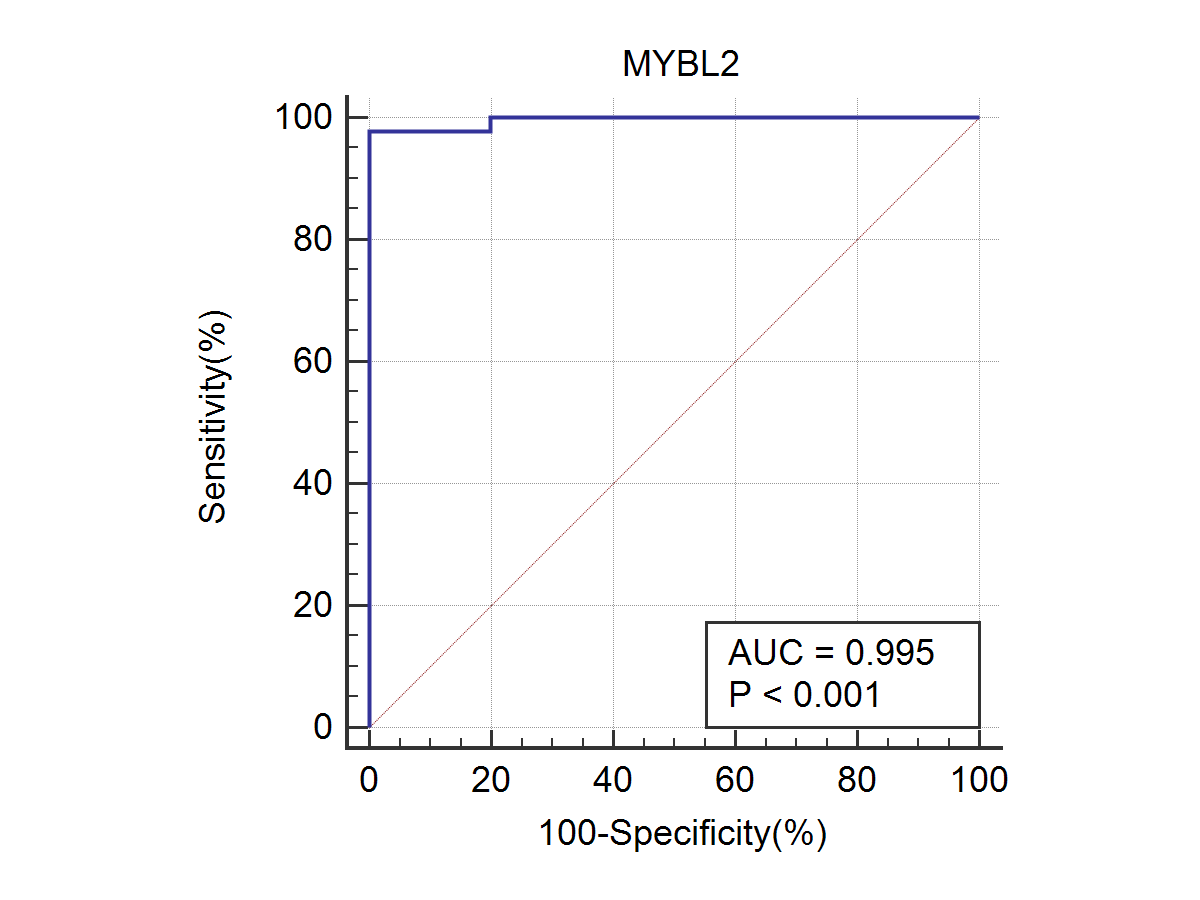

Supplement: Supplementary file 3 [file Data_Sheet_3.ZIP › raw data4/22.ROC/171110roc/MYBL2.png]

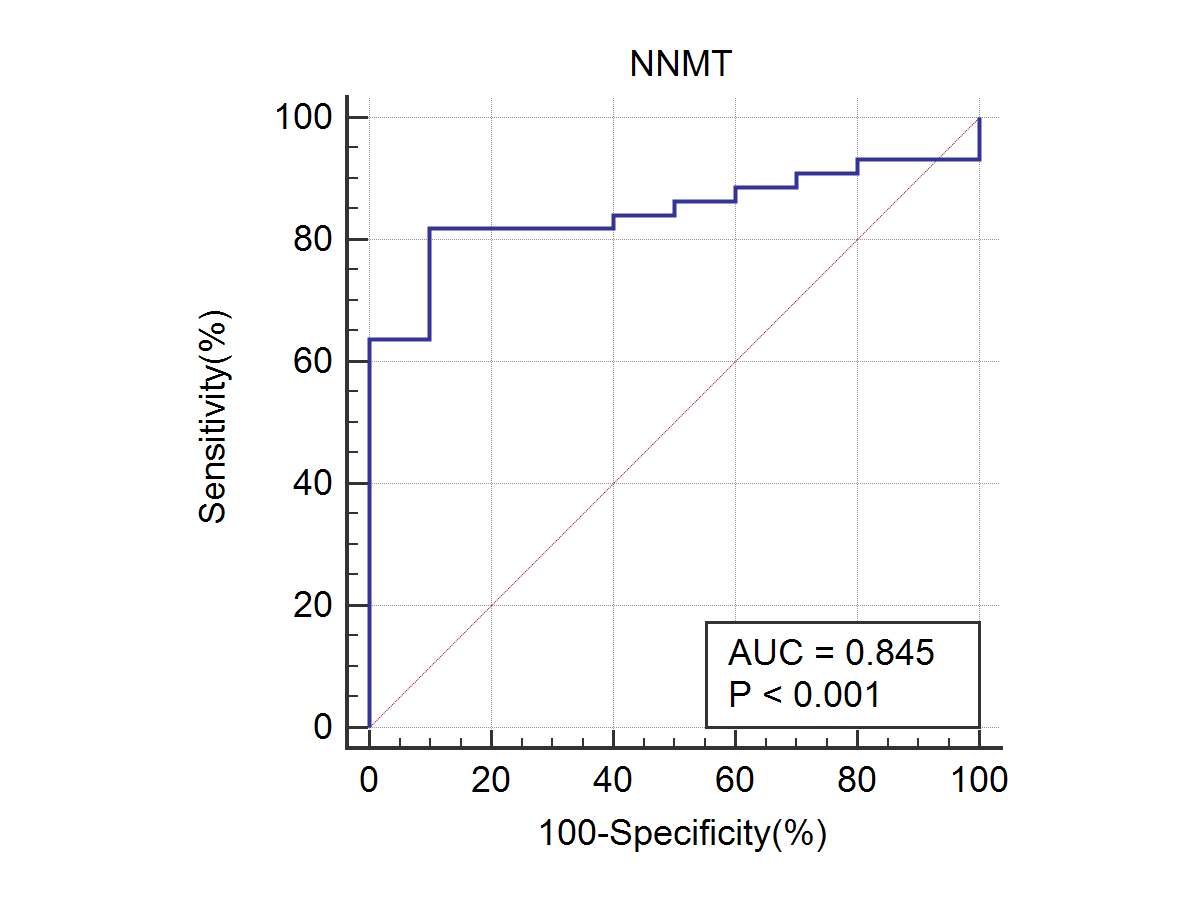

Supplement: Supplementary file 3 [file Data_Sheet_3.ZIP › raw data4/22.ROC/171110roc/NNMT.png]

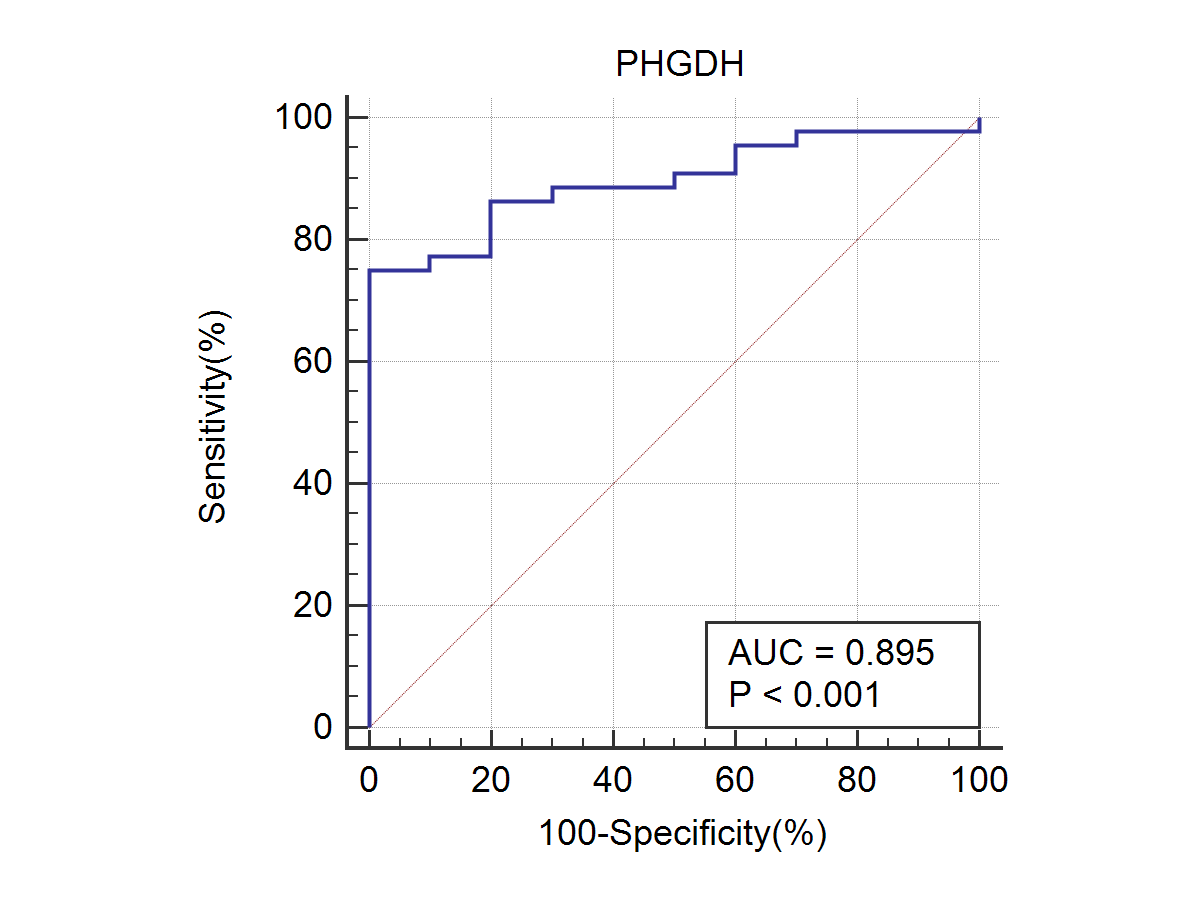

Supplement: Supplementary file 3 [file Data_Sheet_3.ZIP › raw data4/22.ROC/171110roc/PHGDH.png]

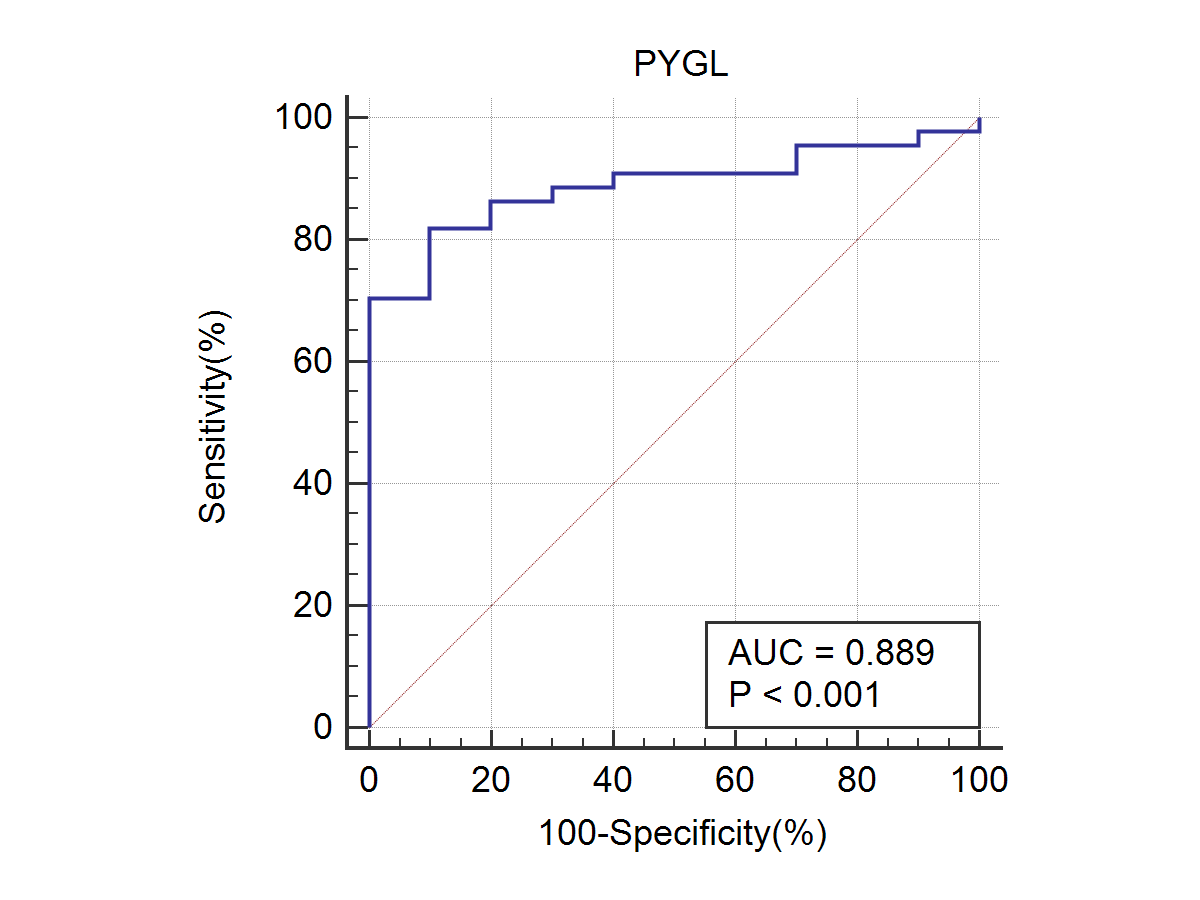

Supplement: Supplementary file 3 [file Data_Sheet_3.ZIP › raw data4/22.ROC/171110roc/PYGL.png]

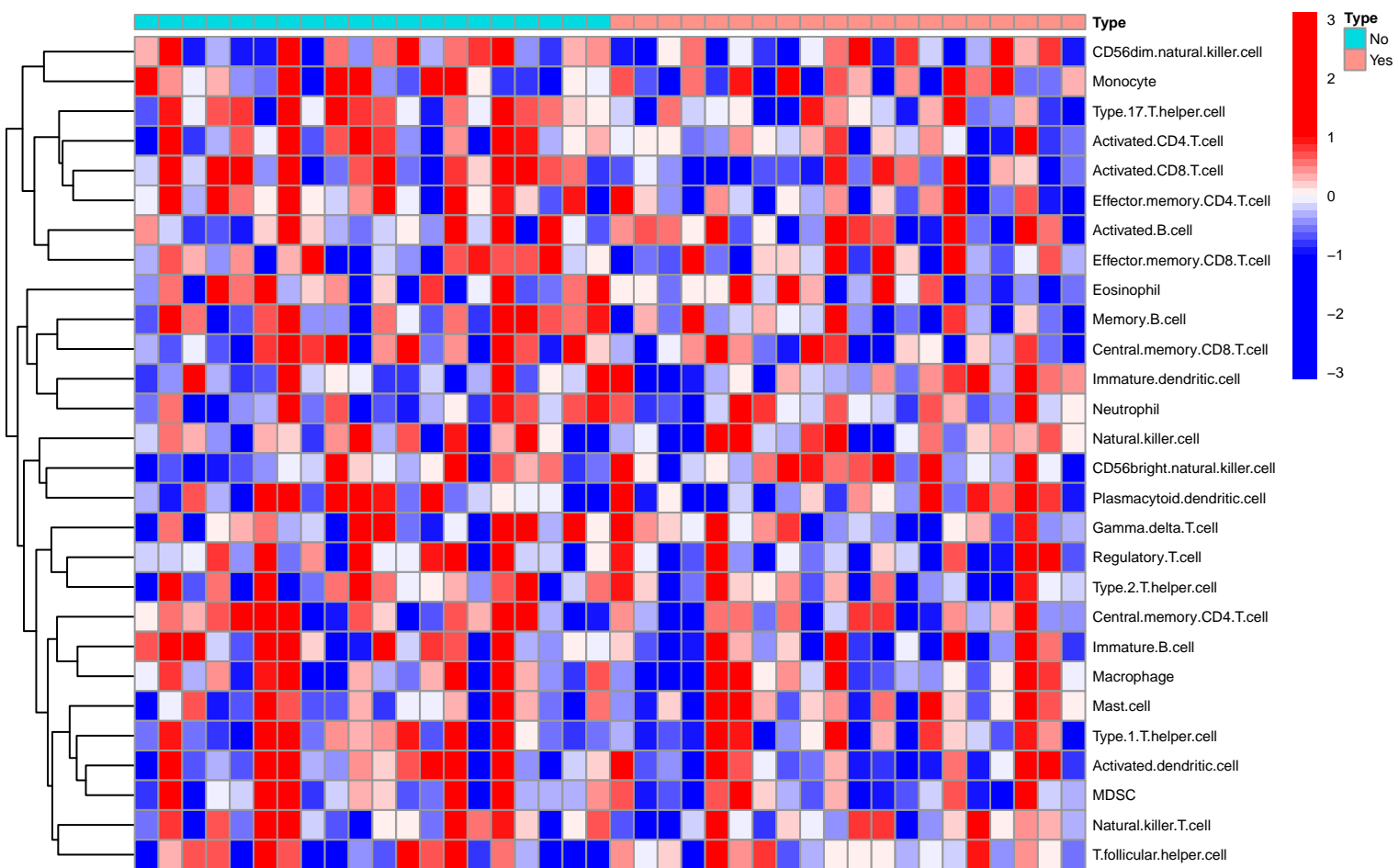

Supplement: Supplementary file 4 [file Data_Sheet_4.ZIP › raw data5/23.immune/GSE111016/2.heatmap/heatmap.pdf]

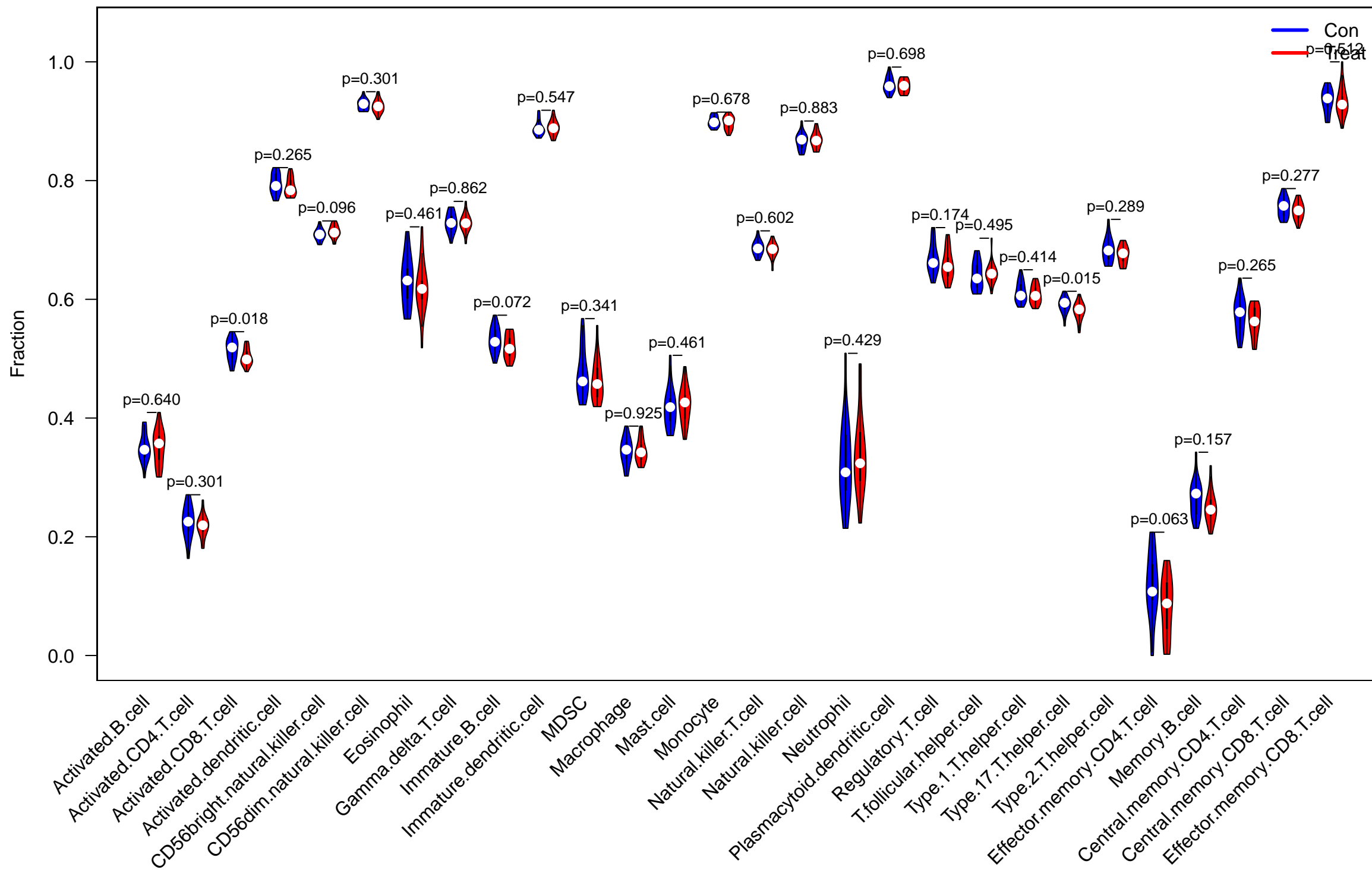

Supplement: Supplementary file 4 [file Data_Sheet_4.ZIP › raw data5/23.immune/GSE111016/3.vioplot/vioplot.pdf]

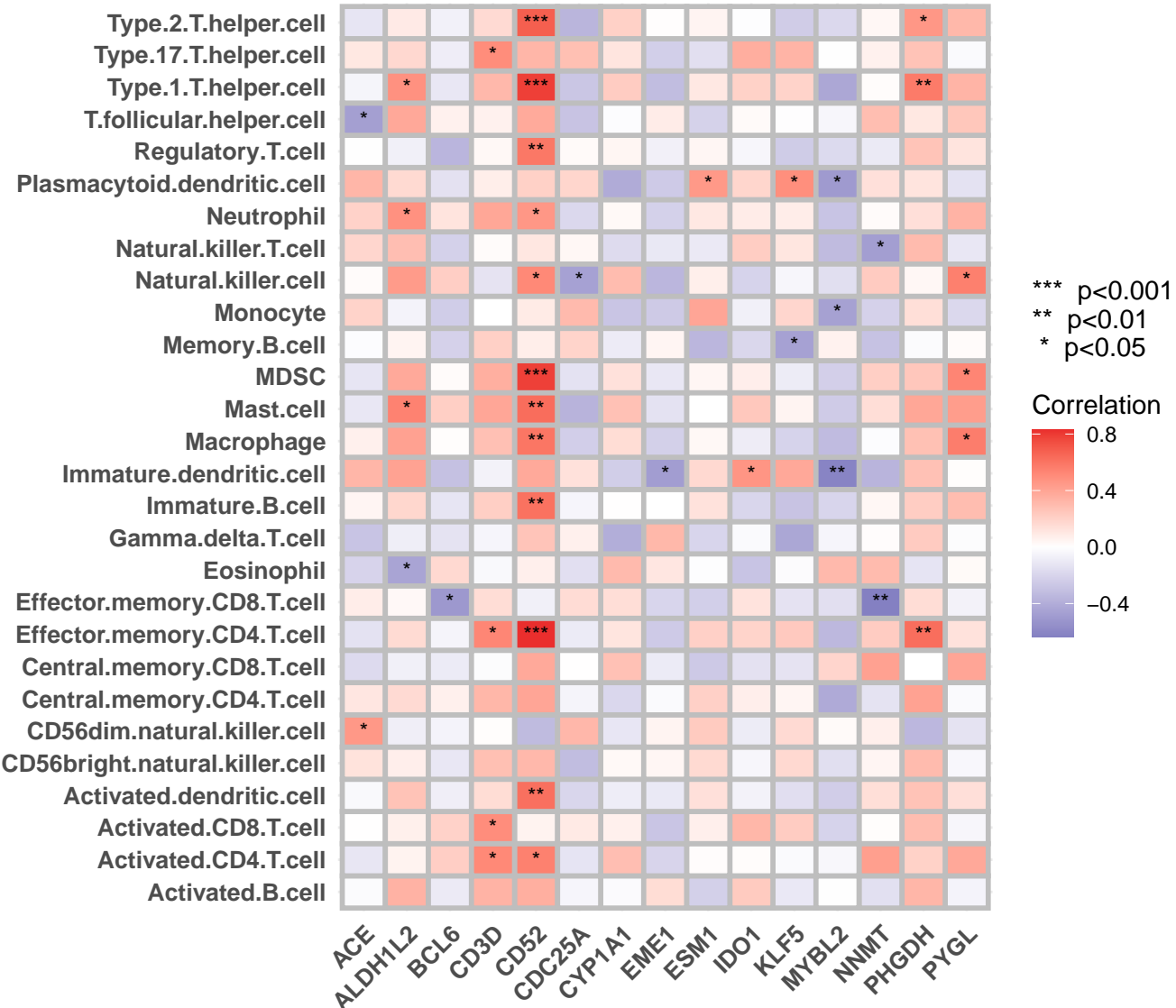

Supplement: Supplementary file 4 [file Data_Sheet_4.ZIP › raw data5/23.immune/GSE111016/4.immuneCor/cor.pdf]

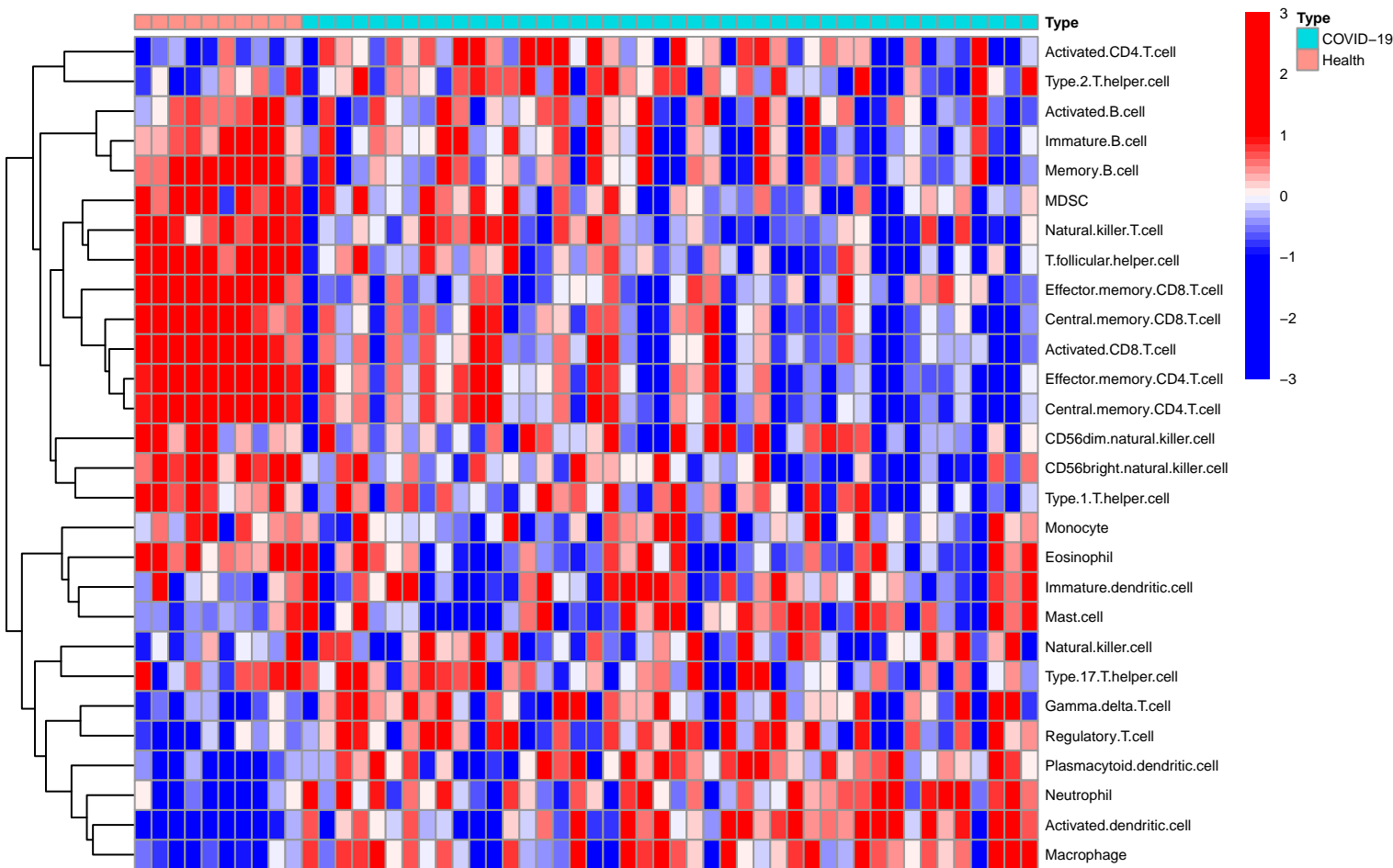

Supplement: Supplementary file 4 [file Data_Sheet_4.ZIP › raw data5/23.immune/GSE171110/2.heatmap/heatmap.pdf]

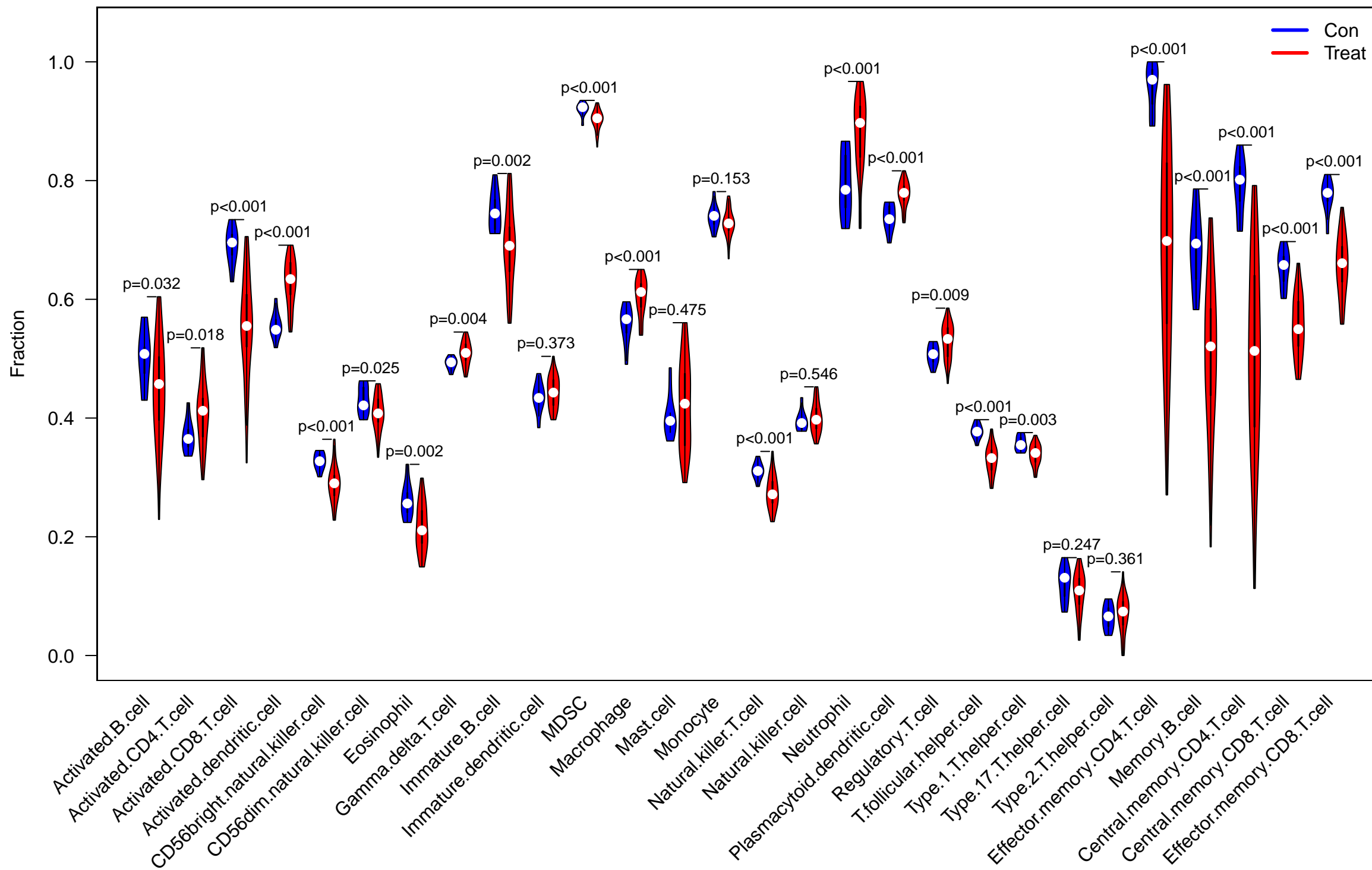

Supplement: Supplementary file 4 [file Data_Sheet_4.ZIP › raw data5/23.immune/GSE171110/3.vioplot/vioplot.pdf]

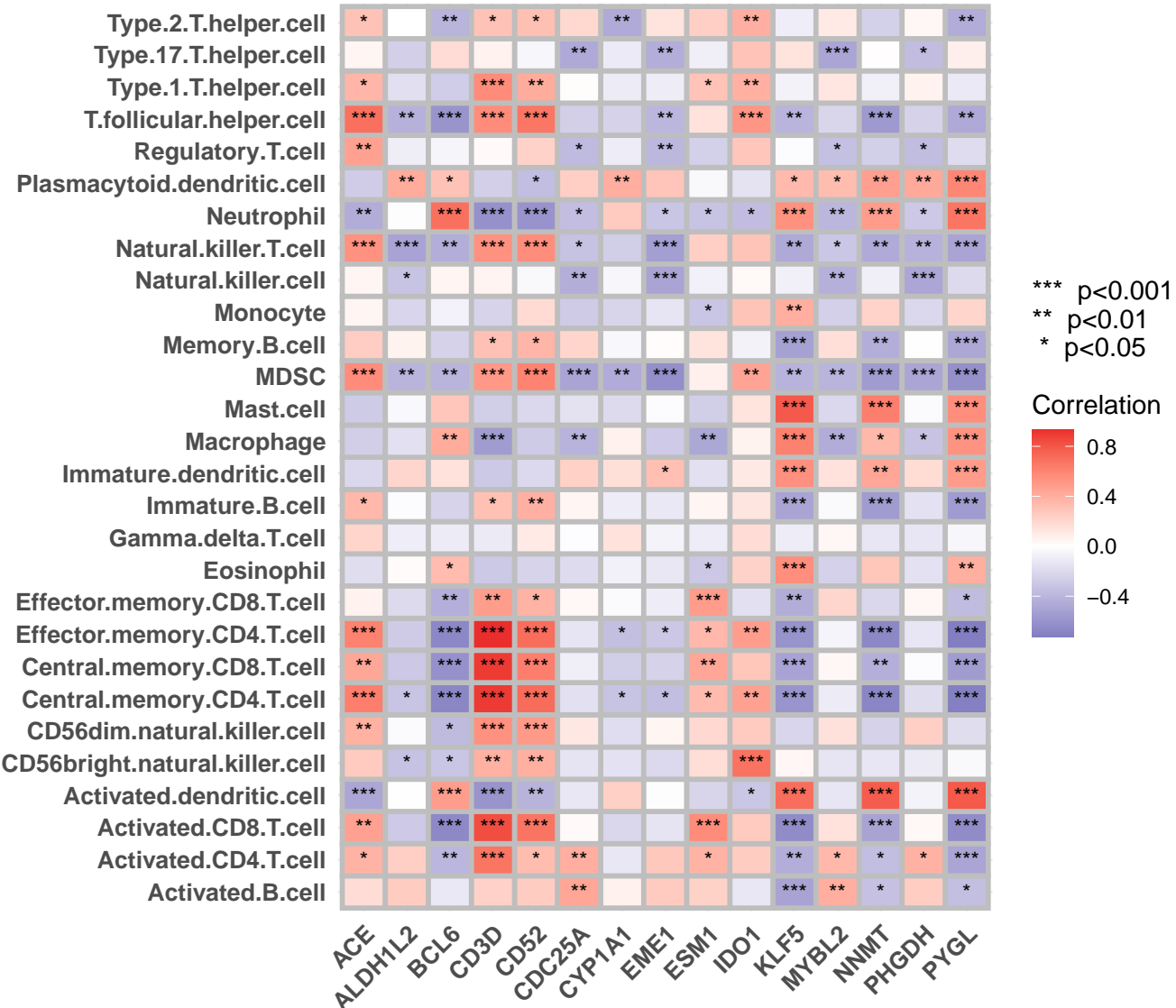

Supplement: Supplementary file 4 [file Data_Sheet_4.ZIP › raw data5/23.immune/GSE171110/4.immuneCor/cor.pdf]

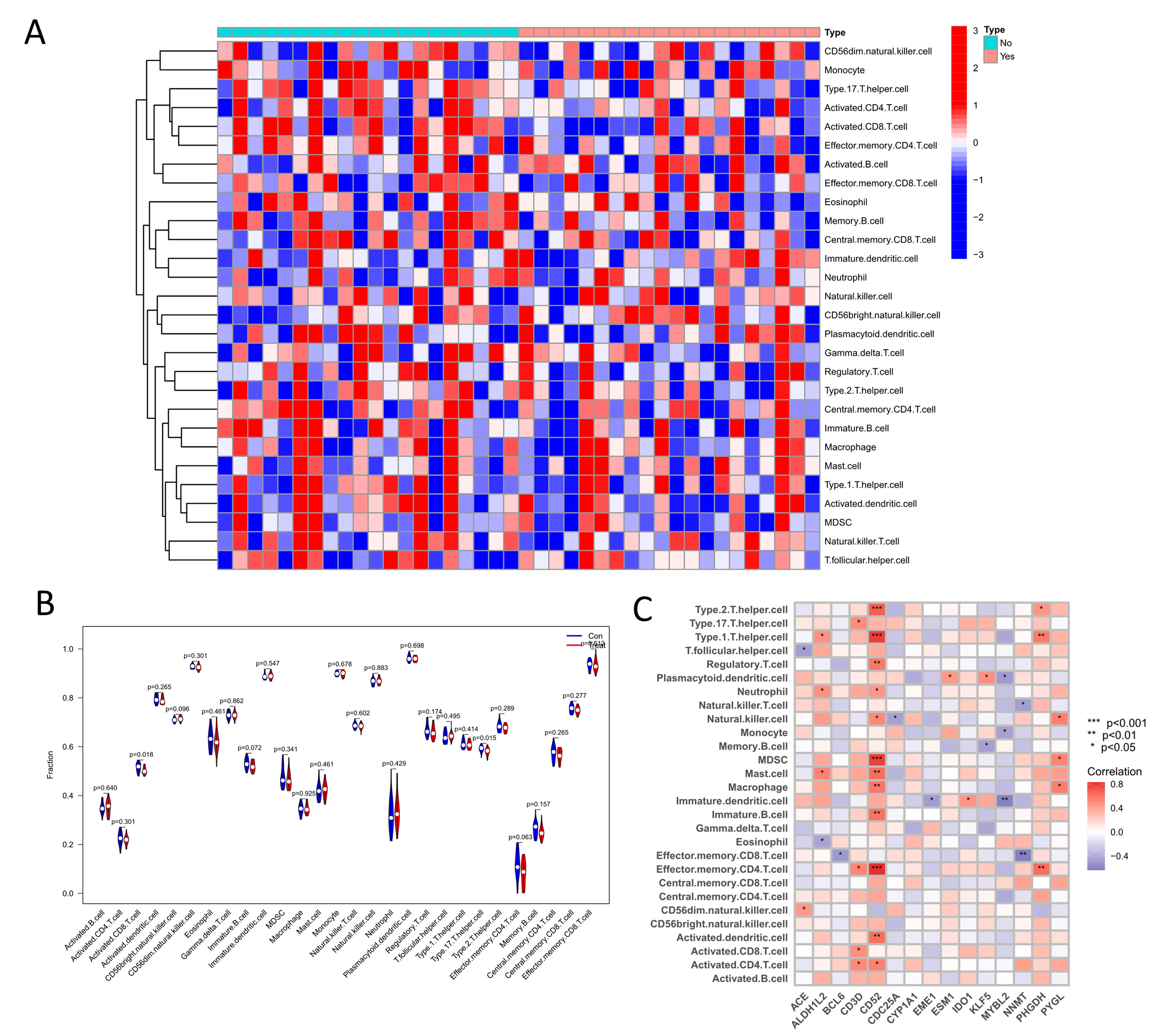

Supplement: Supplementary file 6 [file Image_1.TIF]

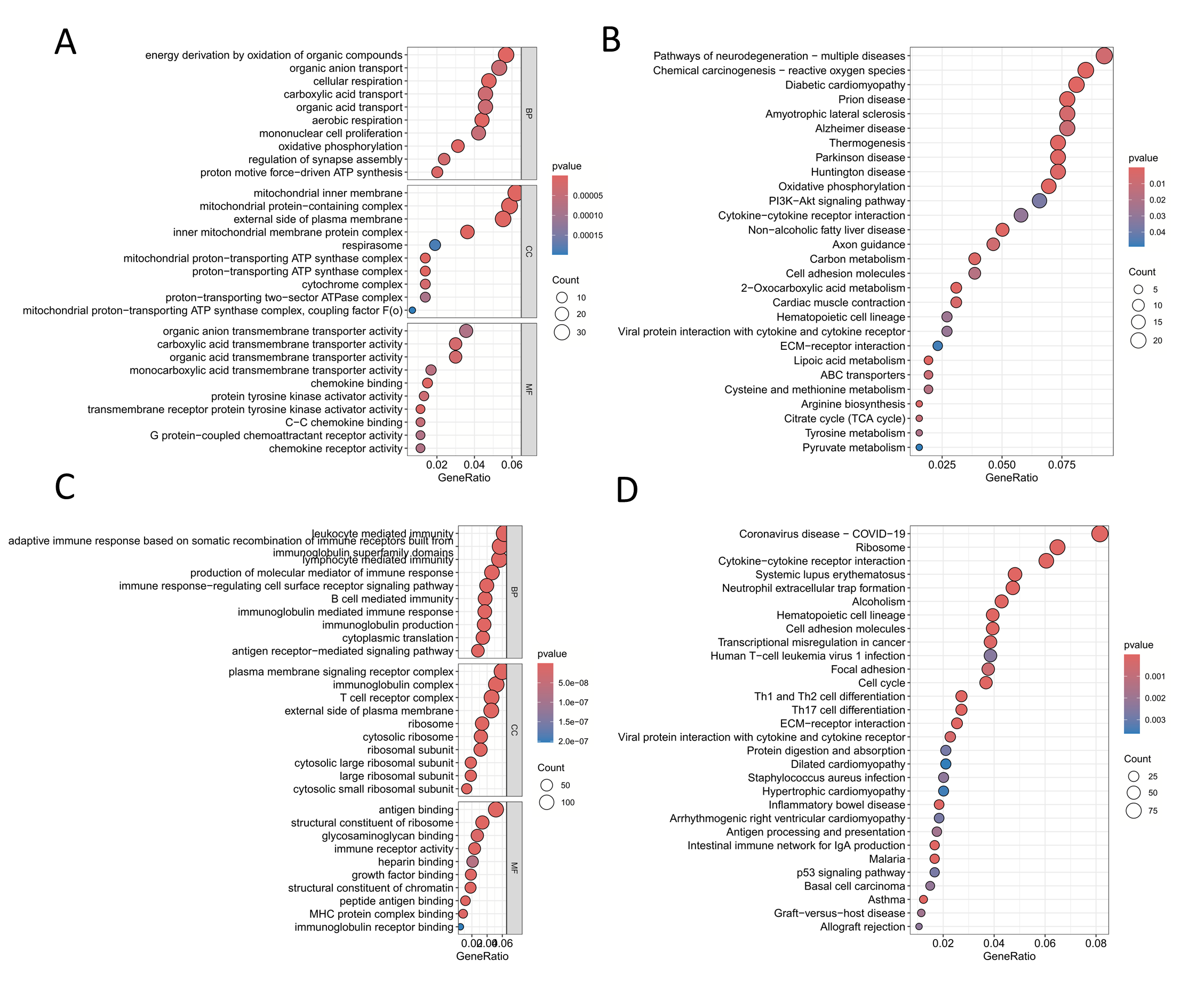

Supplement: Supplementary file 7 [file Image_2.TIF]

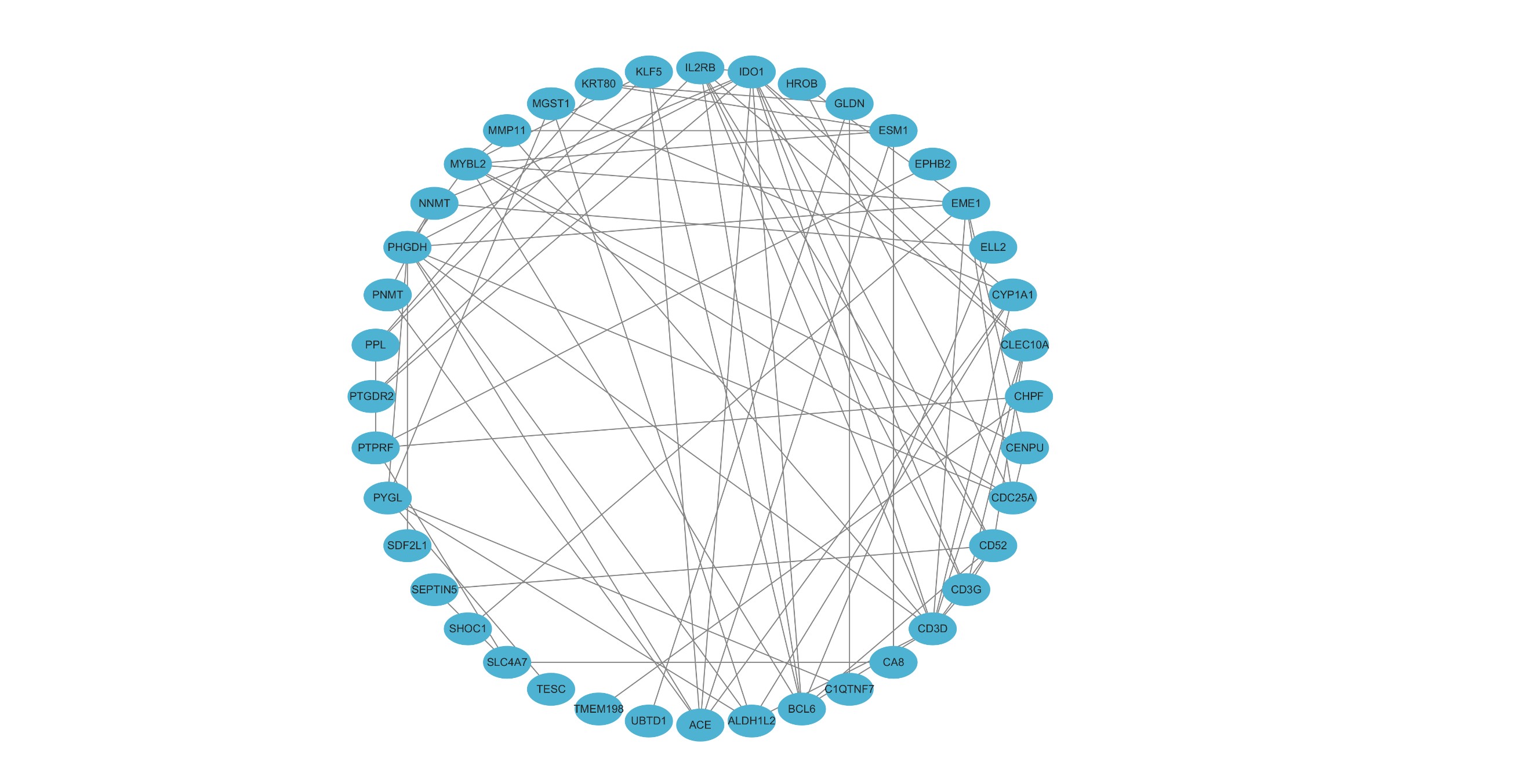

Supplement: Supplementary file 8 [file Image_3.JPEG]
