# Supplementary material for: Bioinformatics and system biology approach to identify potential common pathogenesis for COVID-19 infection and sarcopenia
Source: Front Med (Lausanne). 2024 Jun 24;11:1378846. doi: 10.3389/fmed.2024.1378846 (PMC11228343; doi:10.3389/fmed.2024.1378846)
Supplement: Supplementary file 9 [file Table_1.DOCX]

Supplementary Material

## Supplementary Figure legends

**Supplementary Figure S1. Infiltration analysis and correlation analysis of immune cells in the COVID-19 group and healthy controls group.** (A) Heat map of immune cell subsets in the COVID-19 dataset. (B) Violin diagram of immune cell subsets in the COVID-19 dataset. (C) Correlation of immune cell subsets with common key genes. p< 0.05 indicates statistical difference.

**Supplementary Figure S2. PPI network of common DEG between COVID-19 and sarcopenia.**
